# Polymeric Nanocomposite Membranes for Next Generation Pervaporation Process: Strategies, Challenges and Future Prospects

**DOI:** 10.3390/membranes7030053

**Published:** 2017-09-08

**Authors:** Sagar Roy, Nayan Ranjan Singha

**Affiliations:** 1 Department of Chemistry & Environmental Science, New Jersey Institute of Technology, Newark, NJ 07102, USA; 2 Advanced Polymer Laboratory, Department of Polymer Science and Technology, Government College of Engineering and Leather Technology (Post-Graduate), Kolkata-700106, West Bengal, India; drs.nrs@gmail.com

**Keywords:** pervaporation, nanocomposite membrane, polymer, fabrication, separation

## Abstract

Pervaporation (PV) has been considered as one of the most active and promising areas in membrane technologies in separating close boiling or azeotropic liquid mixtures, heat sensitive biomaterials, water or organics from its mixtures that are indispensable constituents for various important chemical and bio-separations. In the PV process, the membrane plays the most pivotal role and is of paramount importance in governing the overall efficiency. This article evaluates and collaborates the current research towards the development of next generation nanomaterials (NMs) and embedded polymeric membranes with regard to its synthesis, fabrication and application strategies, challenges and future prospects.

## 1. Introduction

The development in current separation technologies has envisioned a green and invincible future that impelled the interest of energy preservation, along with waste minimization and zero discharge all over the world [1]. The concept of a clean and sustainable future has emphasized the integration of energy resources and various processes, as well as the retrieval and reutilization of valuable products from waste streams [2]. To achieve this goal, membrane-based separations may be considered as an auspicious substitution over traditional separation processes.

A membrane is a permselective barrier that allows particular species to pass through it while posing a partition for non-selective species. The membrane technologies are fast-developing and cutting-edge separation technologies that could be extensively employed in environmental remediation, green energy, food, chemical and pharmaceutical sectors [3,4,5,6,7,8]. In general, six major membrane processes, including microfiltration (MF), ultrafiltration (UF), reverse osmosis (RO), electrodialysis (ED), gas separation (GS) and PV have found use in such applications.

**Scheme 1:**
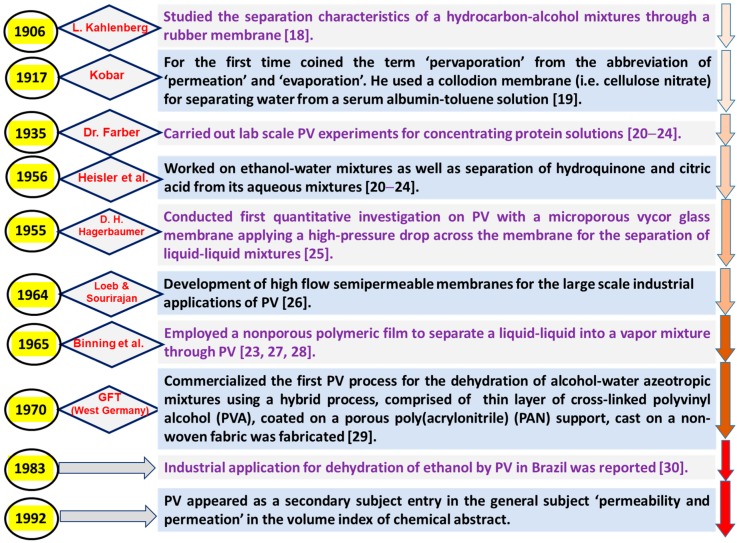
Tree diagram for showing the historical development of pervaporation (PV).

PV is a membrane process involving separation of liquid mixture through a dense selective layer of an asymmetric membrane. PV is being tried extensively on systems which are difficult to separate by the existing separation processes, like distillation, adsorption and extraction. In fact, PV is an effective candidate for separating azeotropic and close boiling liquids, heat sensitive materials, organic mixtures along with removal of dilute volatile organic compounds (VOCs) from wastewater and recovery of volatile aroma compounds from fruit juices [9,10,11,12,13,14,15,16,17]. PV permits the separation of azeotropic mixtures without using a third component that may steer towards undesired side effects, like hydrolysis. Although research and development on PV membranes and process have recently attracted utmost attention of the scientific community, Kahlenberg qualitatively studied the separation characteristic of a hydrocarbon from its alcohol mixture through a rubber membrane as early as 1906 [18]. In fact, Kobar, in 1917, first coined the term ‘pervaporation’ from the abbreviation of ‘permeation’ and ‘evaporation’, where he used a collodion membrane (cellulose nitrate) for separating water from a serum albumin-toluene solution (300:25 *v*/*v*) [19]. After that, PV has comprehensively been studied by several researchers, as well as industries owing to its eco-/cost-friendly performance potential and simple instrumental design. Although few review papers have summarized the historical development and commercialization of PV [20,21], this has been schematically presented in the present review (Scheme 1) [18,19,20,21,22,23,24,25,26,27,28,29,30].

So far separation by PV has been tested for the following three categories, (i) dehydration of organics; (ii) removal of traces organics from aqueous solutions, (iii) organic-organic separation, etc. With the availability of novel efficient membranes, PV has been increasingly developed for large scale processes and until 1999, more than 90 industrial PV units were established throughout the world [31].

Afterward, around 300 US and European patents on PV were published and more than 100 PV units, mainly dealing with the dehydration of organic solvents, were set up [31,32]. In most of the cases, the optimized solution turns out to be a hybrid process integrating the PV with one or more other separation technologies, such as distillation, or with a chemical reactor in the final stage of separation [33]. As an example, for the dehydration of ethanol, it is first concentrated to 90 wt % by distillation and then followed by its further concentration to 99.95 wt % ethanol by PV. The development and application of industrial scale PV system for organic-organic separation continued challenging, mainly due to the unavailability of appropriate membranes and modules that can handle harsh organic separation environment [34]. However, pervaporative recovery of specialty organic compounds, such as retrieval of heat-sensitive aroma in the food industry, dairy flavor compounds, aromatics removal from gasoline, separation of bio-butanol, has been explored [16]. These compounds are often present at very low concentrations in the mixtures and their recovery with traditional techniques, such as distillation and partial condensation, are not very simple [16]. Recently, the importance of PV separation technology increases noticeably as the requirements and awareness for the green environment increases, and the demand for the supply of more fresh water and reutilization/recycling of wastes multiplies. Being a promising technology, PV has the potential to efficiently solve such burning issues and will scientifically and industrially be viable to address these.

In PV, the membrane itself is the key factor, and hence, various materials including polymeric, inorganic and hybrid have been tried. Among them, polymers are primarily and the most extensively used PV membrane materials owing to their easy processing, satisfactory mechanical stability, controlled and tunable transport properties and low cost. However, the low chemical and thermal stability, poor resistant towards hazardous environment, and especially the inherent trade-off relation between permeability and selectivity limit the applicability of such polymeric membranes. On the other hand, a unique type of membrane, known as mixed matrix membrane (MMM), can be fabricated via employing two or more different materials of diversified physicochemical nature and such membranes possess a continuous phase, usually a polymer, embedded through a second dispersed phase [35]. In MMMs, appropriate selection of different phases is important and in many cases, addition of a small amount of dispersed phase may improve the physicochemical properties and separation proficiencies.

Recently, nanotechnology has been considered as one of the highest potential areas for resolving the technical challenges coupled with the separation and purification technologies. The development of distinct nanostructured materials reformed the conventional perception of separation media, streaming new separation methods that outstrip the contemporary accomplishments with their unique properties. The development of superior membrane materials in terms of both permeability and selectivity, fabricated from novel polymers and other NMs, and their commercialization into long-life stable modular configurations open the paths towards real time application of PV separations. Further, improved cost effective systems and module design, optimized collaboration and integration with existent technologies for enhanced recovery from fermentation, esterification and other bio-separation, reduce the overall capital investment. In this review article, the current research and development of polymeric membranes embedded with novel NMs for next generation pervaporative separation and purification process, concerning its synthesis, fabrication and application strategies, challenges and future prospects has been explored.

## 2. Fundamental Theories of Pervaporation

Studies on the separation mechanism proposed a number of opinions on how the transport of a particular component takes place through the membrane. Initially, it was proposed that the selectivity of the membrane raised in the boundary layer between liquid and gaseous zones in the membrane [23] and may be a result of selective permeation through the polymer crystal [36]. The importance of the specific interactions like, H-bonding, between polymeric chains and permeate, was rationally understood/identified [37]. However, previously, diffusion and concentration gradients, in the different solvent components, were believed to be the key driving force for the PV process [38]. However, among these approaches, majority of the researchers believe that PV takes place through a dense permselective membrane, where the liquid feed mixture in contact with one side of the membrane being selectively absorbed and diffused through the membrane, followed by removal from the downstream side.

Transportation of a component across membrane takes place when a driving force, i.e., a potential difference acts on the individual component in the system. The potential difference that arises may be due to differences either in pressure, concentration, temperature or electrical potential. Depending on the nature of the membrane and the species to be separated, the mode of transport through a membrane can be categorized as passive, active or facilitated type. In passive transport, the membrane acts as a barrier and the permeation of components is determined by their diffusivity and concentration in the membrane or simply characterized by their size. The driving force in this type of transport is the gradient in potential. Another form of passive transport is ‘facilitated’ transport or ‘carrier-mediated’ transport. In this case, the transport of a component across a membrane is enhanced by the presence of a carrier, which is mobile in nature. In active transport, the driving force for transport is achieved by a chemical reaction in the membrane phase.

### 2.1. Parameters in Membrane Performance

The PV membrane is mainly characterized by two parameters, flux (*J_i_*) and the separation factor (SF) ($\alpha_{j}^{i}$). Now, *J_i_* can be determined by measuring the permeant mass/volume per unit membrane area per unit time using Equation (1).

(1) $$J_{i}=\frac{Q_{i}}{A\times \Delta t}$$

Here, *Q_i_* and *A* represent the quantity (in gram or mole) of the permeate collected in time interval Δ*t* and the effective membrane area, respectively. Again, $\alpha_{j}^{i}$ can be defined using Equation (2).

(2) $$\alpha_{j}^{i}=\frac{Y_{i}/Y_{j}}{X_{i}/X_{j}}$$

Here, *X_i_*/*X_j_* and *Y_i_*/*Y_j_* represent the fractions of the components *i*/*j* in feed and permeate, respectively. In fact, the membrane SF is often called membrane selectivity (MS). A group of authors occasionally report their results in terms of enrichment factor (β), which is the ratio of concentrations in the permeate to the feed. However, both *J_i_* and $\alpha_{j}^{i}$ not only depend on the intrinsic properties of the membrane used, but they are also a function of the experimental conditions, from which the problem of reporting the data in terms of fluxes and SFs originates [39,40]. In fact, a small change in the operating conditions or slight modification in the membrane could change the results significantly. It is therefore very difficult to compare the PV results between sets of data obtained under different operating conditions. Considering these factors, a better way to represent the PV data is membrane permeability (*P_i_*), permeance (*P_i_*/*l*) and selectivity (α*_ij_*) [39].

In PV, the overall performance of the membrane is often evaluated in terms of permeation separation index (*PSI*) [41], which can be expressed by Equation (3).

(3) $$PSI=J_{i}(\alpha -1)$$

However, from the Equation (3), it is clear that when α = 1, no separation takes place, and zero *PSI* indicates either zero flux or zero separation.

### 2.2. Transport in PV

In PV process, transport through a membrane may be described by the two main mechanisms, which are discussed below.

#### 2.2.1. Preferential Sorption-Capillary Flow (PSCF) Mechanism

In 1987, Sourirajan and Shiyao proposed this mechanism as a combination of RO separation followed by evaporation and gas/vapor transport through capillary pores on the surface layer of the membrane [42,43]. In fact, in this mechanism, effective molecular size of the permeants, pore size and its distribution in the membrane and the specific interaction between the permeant and the membrane material regulates the separation. The liquid feed flows through the tiny cylindrical pores in the dense membrane and evaporates as vapor from the pore outlet in the downstream side under low pressure. However, the assumption of cylindrical pores, effect of the size and distribution limits its acceptability in explaining the separation characteristics. The model is also unable to justify the inverse relation of flux and membrane thickness, membrane swelling, and trade off relationship of flux and SF.

#### 2.2.2. Solution-Diffusion (SD) Mechanism

SD mechanism is the most widely accepted transport mechanism for PV, which mainly consists of three steps: first, sorption of the liquid feed mixture into the membrane at the upstream side; second, diffusion of the sorbed component through the membrane due to the presence of concentration gradient, and finally desorption of the permeants at the downstream side under vacuum as vapor [44]. Thus, the separation is achieved via the differentiation in solubility and diffusivity of various species into the membrane matrix. Desorption at the downstream side under low pressure is very fast and does not contribute any effect in separation performances. In the SD model, it is assumed that the pressure within the membrane is quite uniform and the concentration gradients present across the membrane are expressed in terms of the chemical potential gradient [44].

##### Sorption of the Permeants

The sorption of the permeants occurs as a result of the activity gradient when the liquid mixtures come into contact with the dry polymer membrane surface. Sorption is a thermodynamic phenomenon that reaches equilibrium as soon as the activity of the sorbed species, into the polymer matrix, becomes equal to the activity of the bulk liquid. The relative sorption of the permeants depends on the relative solubility of the individual species into the membrane matrix. The solubility, which is a measure of the amount of permeant sorbed by the membrane, varies with different species due to the presence of specific interaction between the permeants and the membrane material. The separation is obtained because of this preferential sorption among liquids mixture into the membrane matrix. The solubility parameter (SP) theory, based on free energy of mixing (Δ*G_M_*), implies that the preferential sorption takes place when the SPs of both polymer and the permeant species are very close. Another important factor is the interaction parameter that determines the affinity of a polymer for a particular species.

##### Diffusion through the Membrane

The variation in the rate of diffusion for different sorbed species gives the desired separation of that particular species. The rate of diffusion depends on several physical and chemical factors including, size and shape of the permeant molecules, mutual interaction between the polymer and the diffused component. In general, low molecular weight (MW) and molecules with smaller cross-section move faster. Movement of a large molecule requires breaking of higher number of secondary bonds to accommodate the molecule into a vacant space or hole [45]. Another theoretical model was proposed for the diffusion of spherical and quasi-spherical molecules by Peppas and Reinhart that accounts the effect of plasticization of polymer chains during sorption. This model demonstrates that the diffusion coefficient (DC) not only depends on the size or MW, but also on the other structural characteristics of the polymers, such as degree of swelling, crosslink density (CD), etc. [46].

##### Transport Equation through the Membrane

The transport of the *i*th component through any membrane can be described by Fick’s first law as expressed in Equation (4).

(4) $$J_{i}=-D_{i}\frac{dc_{i}}{dx}$$

Here, *J_i_* is operating through a plane perpendicular to the direction of diffusion, and is proportional to the concentration gradient (d*c_i_*/d*x*). The proportionality constant, i.e., *D_i_*, is known as the diffusivity. As *D_i_* is a function of membrane phase concentration of the permeants, hence the above equation may be modified as Equation (5).

(5) $$D_{i}=-D_{i}^{0}f(C_{i})\frac{dc_{i}}{dx}$$

Here, $D_{i}^{0}$ is the DC of *i*th component at infinite dilution.

Now, integrating Equation (5) over the entire membrane thickness *l*, the above equation is modified to Equation (6).

(6) $$J_{i}\int_{0}^{l}dx=-D_{i}^{0}\int_{C_{if}}^{C_{ii}}f\left(C_{i}\right)C_{i}dc_{i}$$

Here, *C_ii_* is the membrane phase concentration of *i*th component that can be calculated from its bulk concentration by Henry’s equation, when it is present in trace amounts. However, *C_ii_* may also be obtained from the sorption data. Again, *C_if_* is the membrane phase concentration on the permeate side of the *i*th component, and may be neglected as the activity of the component in the downstream side is very low due to the low pressure or vacuum. Thus, the equation can be easily solved to calculate the theoretical flux and DC of the components employing any of the above equations of DC and concentration.

The rate of permeation through a polymer membrane depends on both the sorption and diffusion parameters and can be expressed by Equation (7).

(7) $$J_{i}=-P_{i}\frac{dc_{i}}{dx}$$

Here, *P_i_* is the membrane permeabilities, which is the product of solubility (*S_i_*) and diffusivity (*D_i_*). Again, Equation (7) is often reported in terms of *P_i_* or permeances (*P_i_*/*l*) using Equation (8).

(8) $$J_{i}=-\frac{D_{i}K_{i}^{G}}{l}(P_{i_{0}}-P_{il})$$

Here, *l*, *D_i_*, *P_i_*_0_/*P_il_* and $K_{i}^{G}$ are membrane thickness and membrane DC, partial pressures on either side of the membrane and sorption coefficient of *i*th component, respectively. In fact, $K_{i}^{G}$ correlates partial pressure of the gas (*P_i_*) to the concentration (*C_i_*) in the membrane phase. In general, sorption bears a strong relationship with diffusion in polymer matrix. An increase in sorption of solvent molecules in polymer matrix swells the membrane, which promotes the free segmental movement of the polymer chains and lowers the activation energy for diffusion. The permeation of the solvent molecules through the ‘liquid zone’ is much faster in comparison with dry polymer.

In general, the classic SD theory successfully describes the permeation phenomenon through non-swollen membranes, such as traces of selective permeants (VOCs/water) present in the mixtures [47,48]. Mathematical modeling of the pervaporative separation of methanol-methyltertbutyl ether (MTBE) mixtures, based on the generalized Fick’s law and the assumption that transport through the membrane is the rate-limiting step, has been studied using a commercial membrane, Pervap 2256 [49]. This work helps describe the PV mechanisms of azeotropic mixtures. However, when a large proportion of the permeant has to be separated from the mixture, such as the separation of organic-organic mixtures or dehydration, substantial membrane swelling may occur, and both the sorption and DCs become concentration dependent. In such cases, permeation fluxes of the *i*th and *j*th components, through the membrane, can generally be expressed by the Equations (9) and (10) [50].

(9) $$J_{i}=-D_{i0}\exp(\alpha_{ii}C_{i}+\beta_{ij}C_{j})\frac{dc_{i}}{dx}$$

(10) $$J_{i}=-D_{j0}\exp(\alpha_{ij}C_{i}+\beta_{jj}C_{j})\frac{dc_{i}}{dx}$$

Here, *D_i_*_0_/*D_j_*_0_, *C_i_*/*C_j_* and α/β represent DCs of at infinite dilution, local concentrations in the membrane, and plasticization coefficients for the membrane, respectively, for *i*th and *j*th components.

### 2.3. Transport in Nanocomposite Membrane (NCM)

The term NCM usually describes the membrane utilizing the nanoparticles (NPs) dispersed within a continuous phase of binder matrix. The presence of NPs into the membrane matrix affects the transportation of the permeants effectively by creating a preferential permeation trails for the selective species and providing a barrier for non-selective species at the same time.

Casado et al. studied the pervaporative dehydration of organic mixtures using a commercial silica membrane and determined the corresponding kinetic parameters [51]. A semi-empirical correlation was used to fit the water flux data that expressed water flux as an exponential function of the water activity in the feed mixture by Equation (11).

(11) $$\ln(J_{w,mass})=\ln\left(J_{0,w}(T)\right)+\zeta a_{w}^{f}$$

Here, ζ and $a_{w}^{f}$ represent model parameter and water activity of feed solution, respectively. However, the term *J*_0,*w*_*(T)* can be defined by Equation (12).

(12) $$J_{0,w}(T)=\frac{\rho^{m}D_{w,0}}{\delta \tau }$$

Here, ρ*^m^*, δ and τ represent mass density at the membrane phase, selective layer thickness and the exponential parameter of diffusivity in the membrane, respectively, and it follows an Arrhenius type Equation (13).

(13) $$\ln\left(J_{0,w}(T)\right)=\ln J_{00,w}-\frac{E_{act}}{RT}$$

The semi-empirical equation was used to predict the membrane performance and validate the applicability of the equation. It was observed that the water flux through the membrane depends on the water activity in the feed liquid mixture and the water flux provided by the commercial silica membrane are larger than that of the reported Pervap SMS membrane for the dehydration of industrial acetone mixtures [51].

In another approach, the models proposed by Maxwell and Bruggeman describe the overall permeability as a function of the permeabilities of the polymer matrix phase and the filler phase, and the amount of the filler. These models successfully predict the permeability through polymer-inorganic NCMs. The Maxwell model suggested that addition of inorganic nanofillers (NFs) to a polymer matrix typically reduces the permeability of the permeant. In fact, such NFs not only reduce the membrane solubility, via decreasing the available volume for sorption, but also decrease the diffusivity due to the expansion of the penetrant diffusion pathway length resulting from an increase in tortuosity [44]. In succession, Barrer et al. simplified the Maxwell model (Equation (14)), particularly suitable for the composite membranes containing impermeable spherical particles [52].

(14) $$P_{eff}=P_{c}\left(\frac{1-\phi }{1+0.5\phi }\right)$$

Here, *P_eff_*, *P_c_* and φ are permeability of NCM, permeability of permeant in the pure polymer matrix and the volume fraction (VC) of NFs, respectively. This model explained the reduction in permeability of some NCMs [53,54]. However, further researches on the transport phenomenon of the NCMs indicate the presence of other factors that were not considered in the Maxwell model [55,56,57,58]. The studies on gas transport behavior with polyimide-silica NCM reveled an increment in permeability with the increase in nanosilica content in the matrix [59]. The Maxwell model did not consider the interactions between the NFs and the polymer chains, and between the NFs and the penetrants. The alteration in polymer or NF chemistry via physicochemical modification enhances the interfacial interactions that change the solubility and diffusivity of penetrants significantly. The Maxwell equation is also limited to the membranes with low filler content and it did not consider the distribution of the filler in the matrix.

The Bruggeman model, originally developed to analyze the dielectric constant of particulate composites [60], was recommended by Bouma et al. to model the permeability in NCMs [61]. This model includes the effect of incorporation of additional spherical particles to a random dilute suspension by an integration technique by using Equation (15).

(15) $$\left(\frac{P_{eff}-P_{d}}{P_{C}-P_{d}}\right){\left(\frac{P_{eff}}{P_{C}}\right)}^{-1/3}=1-\phi_{d}$$

Here, *P_d_* and φ*_d_* represent permeability of a permeant in a dispersed phase and VC of the second phase in the total membrane, respectively. However, the prediction of permeability using both the Maxwell and Bruggeman models is acceptable only up to φ*_d_* = 0.20, and beyond this, the presence of surrounding particles may affect the flow patterns [62] and Bruggeman model behaves better since it accounts for this behavior. In fact, this model can further be reduced to the following equations depending on the nature and amount of the dispersed phase. If the composite phase comprises an impermeable dispersed phase (i.e., *P_d_* = 0), this model can be written as the Equation (16).

(16) $$\frac{P_{eff}}{P_{C}}={\left(1-\phi \right)}^{3/2}$$

However, the formation of a significant amount of voids in the membrane is observed when NPs are highly incompatible with the polymer matrices. Such void spaces are considered as the dispersed phase and much more permeable than the polymeric phase. Thus, if the permeability of the dispersed phase is higher than the matrix, i.e., *P_d_* > *P_C_*, the model can be rewritten as Equation (17).

(17) $$\frac{P_{eff}}{P_{C}}=\frac{1}{{\left(1-\phi \right)}^{3}}$$

This equation was observed to be well matched with the experimental data and the change in permeability can also be predicted satisfactorily [63].

### 2.4. Effect of Process Conditions

#### 2.4.1. FEED Concentration

The permeation of a component in PV occurs via SD mechanism, which is highly dependent on both solubility and diffusivity of the corresponding species into the dense polymer matrix. Concentration of a particular species in the feed side regulates both the solubility and diffusivity, hence the separation performances. In general, it is observed that the flux increases with increase in concentration of the component in the feed, but exhibits a reverse trend for SF.

#### 2.4.2. Feed and Permeate Pressure

The driving force in PV is the activity gradient of the components across the membrane, which in turn is dependent on the partial pressure of the species. The feed side pressure influences the PV performances notably only when the permeate side pressure is higher. The permeate side pressure affects the PV characteristics significantly as the activity of the species is directly related to the permeate pressure. Indeed, the lowering in the permeate side pressure from the saturation pressure of permeates allows the components to pass through the membrane and flux reaches the maximum as the permeate pressure becomes zero. The change in permeate pressure can also affect the SF. In fact, at higher permeate pressure, the rate of desorption slowed down the influence on the overall selectivity. However, the nature of the change completely depends on the relative volatility of competitive species at the downstream side. In general, reduction in permeate pressure results in higher flux and good SF.

#### 2.4.3. Effect of Temperature

In view of the fact that both of solubility and diffusivity of the components are strongly temperature dependent, it is expected that the transport of permeant through PV membranes are also reliant on the temperature. In fact, the flux generally increases with the increase in feed temperature, following an Arrhenius-type Equation (18).

(18) $$J=J_{0}\exp\left(\frac{E_{p}}{RT}\right),\ln J=-\frac{E_{p}}{RT}+\ln A$$

However, activation energy (i.e., *E_p_*) can be obtained from the slop of the linear plot of ln*J* vs. 1/*T*. Since, the diffusivity of the permeants increases with increase in temperature, the permeation rate also increases. In fact, at higher temperature, the enhanced movement of the polymer chains, assisted by the thermal energy, also facilitates the easy permeation of the sorbed molecules through the membrane. The SF usually decreases with increase in temperature for most of the components.

#### 2.4.4. Effect of Membrane Thickness

The SD model suggests that the permeability of a component remains unchanged with the thickness of the membrane. However, flux is observed to vary inversely with the membrane thickness [64], since an increase in the membrane thickness enhances the permeation resistance.

#### 2.4.5. Concentration Polarization (CP) and Mass Transfer Coefficient (MTC)

In PV, as the components in the feed mixture permeate through the membrane at different rates, a variation in the concentration of the permeating and non-permeating species near the membrane surface builds up gradually. Consequently, the concentration of the preferred molecules in the solution, adjacent to the membrane surface, becomes lower than that in the bulk fluid. Meanwhile, the solution becomes strengthened in the non-permeating or less-permeating molecules. There is also a decrease in fluid velocity from the bulk feed to the stationary membrane, which boosts the concentration gradient. The formation of concentration gradient is known as the CP, which lowers the flux and MS by reducing the driving force across the membrane. In PV, CP is not a severe problem as the permeation rate is much lower compared to the other pressure driven separation process and the permeants usually do not retain on the membrane surface. However, for highly selective and highly permeable thin membrane the CP becomes important. The mass transfer resistance in the boundary layer can be represented by Equation (19).

(19) $$\frac{1}{Q}=\frac{1}{KL}+\frac{1}{DS}$$

Here, *Q*, *K* and *L* represent flux, MTC for boundary layer and membrane thickness, respectively. Usually, flux and SF decreases with CP and the effect of CP is more pronounced with decreasing MTC and concentration of the preferentially permeating species. It was observed that the experimental MTC also depends on the conditioning of the PV system. The steady-state regime normally attains a certain delay after starting the PV operation. In this context, Rautenbach and Hommerich studied the dynamic mass transfer effect on PV system that includes both the models on a macroscopic level for time dependent membrane separation performance, and the models for the technical PV unit itself [65].

## 3. Membranes for PV Applications

A membrane can be defined as a permselective barrier interposed between two phases. It imparts separation through controlled and selective mass transfer of one of the components to be separated from one bulk phase to other. In fact, the membrane can be classified as porous or non-porous, thick or thin, and its structure can be homogeneous or heterogeneous. Based on the origin, membranes can be classified as natural or synthetic, neutral or charged. The membranes show a wide range of variation in the physical structure and separation characteristics [66]. In fact, depending on morphology, membranes can be classified into two categories, viz. symmetric or asymmetric. The symmetric membranes refer to the membranes, which possess featureless uniform morphology throughout the entire membrane thickness. Such membranes are fabricated from single or a blend of polymers and are usually found to exhibit higher selectivity, but poor permeate flux. Conversely, asymmetric membranes have a gradient in structure. The asymmetric membranes can be characterized into two major categories: one where the top selective layer is integrated with the support made from the same material and the other is composite membranes where more than one distinct phase is present. The presence of different polymers and other materials in the composite membrane offers enhanced membrane properties that may not be obtained in symmetric membranes. The incorporation of a small amount of NPs not only drastically changes the physical and thermal properties, but also influence the separation characteristics significantly. However, the interface differentiation, between the polymer and the NPs, and the presence of a strong agglomeration tendency of these NFs make the fabrication of a suitable NCM highly critical.

The membranes synthesized through dispersion of nanosized particles in the matrix of a single polymer, copolymer or polymer blends exhibit more interesting properties. Although the symmetric dense membrane can provide high selectivity, overall flux is usually low owing to higher mass transfer resistance. Thus, the membranes fabricated by using an asymmetric structure consist of a thin dense selective skin layer over a thick porous substrate that provides the mechanical strength of the thin selective barrier layer are of special interest. The mass transfer rate can be substantially increased by using the asymmetric membrane structures.

In conventional PV membranes, permeability and selectivity trends show typical “trade-off” relationship, which means that a highly permeable membrane with excellent selectivity is rather difficult to attain. It is thus important to develop advanced membranes for more challenging future applications. The initial approach was to fabricate MMMs with polymer-inorganic fillers, such as, zeolites, silica and carbon molecular sieves etc. where, fillers or NFs are dispersed in a polymer matrix [67,68,69,70,71,72,73]. Recently, Albo et al. studied the structural characteristics and gas transport properties of interfacially polymerized in-homogeneous top layer of thin-film composite membranes [74,75]. The nano ordered free-volume pore size of the prepared polyamide (PA) membranes were pretreated by various methods and evaluated by nanopermporometry (NPP). The membrane was quantitatively compared with the free volume pore, estimated from the normalized Knudsen based permeance (NKP) and with positron annihilation characterization (PALS). Those studies revealed the bi-modal structure of material and the transport mechanisms occurring. It was also observed that the membrane structure has been influenced significantly by the drying procedure and is therefore crucial in controlling the separation performances.

Nanocarbon based MMMs have recently generated great interest because of their unique properties. Carbon nanotubes (CNTs), nanodiamonds (NDs) and graphene oxides (GO) have specifically attracted significant attention due to their superior separation performances. Structurally, CNTs are the tubular graphite sheets rolled-up along a central axis with a diameter within nanometer range. Due to strong van der Waals forces present between the individual CNTs, these tend to agglomerate into bundles [76]. This is a very common problem working with NMs. The differentiation in their physical and chemical properties, especially the difference in density between the NMs and the polymers leads to create non-uniform distribution of the NMs in the polymer phase [71]. This may eventually trigger the formation of non-selective defects, such as pinholes in the membranes. Thus, the efficiency of NMs depends on the effective dispersion into the polymer matrix. Modification of the NMs surface via chemical treatment or functionalization is believed to be an effectual way to restrict the agglomeration tendency that facilitates uniform dispersion of NMs in the membrane matrix [77,78]. Due to the enhanced sorption-desorption phenomenon and faster transport over the CNTs surface makes it perfectly fit for the PV applications [79,80,81,82,83,84,85,86]. Among other NMs, silica, zeolite and titanium oxide (TiO_2_), has been used widely by various researchers [1,87,88,89]. These PV membranes have been applied extensively in three major fields of separations as described below.

### 3.1. Dehydration of Organics

Membranes, prepared from hydrophilic polymers, are generally employed for the dehydration of organic solvents. Various polymeric membranes have been reported for this separation process [90,91]. Polyvinyl alcohol (PVA) is one of the most extensively used polymers from where hydrophilic membranes with good mechanical strength can be obtained. PVA is completely soluble in hot water and needs to be crosslinked to maintain the structural integrity during the PV process. It may be crosslinked with a number of aldehyde/ketone at a certain pH or any mono-/poly-carboxylic acid to produce a water insoluble membrane having water permeability [92,93,94,95,96]. In succession, Kang et al. reported the fabrication of PVA/poly(styrene sulfonic acid-co-maleic acid) (PSSA-MA) water soluble membrane [92]. Moreover, Huang and Yeom used polyamic acid as a crosslinker for PVA for ethanol dehydration [93]. Again, hydrophilicity of PVA was further increased by grafting maleic anhydride/methyl methacrylate and used for ethanol dehydration [94]. In another study, Kang et al. modified the membrane surface of crosslinked PVA by chemical reaction with monocholoro acetic acid [95]. This modification increased water selectivity by a factor of 2 compared to the ordinary crosslinked PVA membrane when applied for dehydration of ethanol. The major drawback of PVA membrane is that it cannot be used for dehydration of corrosive liquids, i.e., for dehydration of any acid or base, which is interesting for pervaporative separation as relatively lower volatility of such aqueous acid or base restricts conventional distillation. A new alcohol dehydration membrane, PVA-chitosan (CS) blended composite membrane (PVA-CS), possessing promising selectivity (~500) and permeability (200 g m^−2^ h^−1^), especially in separating ethanol-water near the azeotropic region at 70 °C, has also been reported [96]. Again, Zhang et al. studied PVA-CS membrane for pervaporative dehydration of n-butyl acetate-water and n-butyl acetate-n-butanol-water type binary and ternary systems, respectively [97]. In fact, with increase in CS fraction, degree of swelling in water was found to reduce significantly. A very high SF of 27,000 with a total flux of 402 g m^−2^ h^−1^ was obtained using the blend membrane containing 25 wt % CS at 40 °C for the binary system. Dense membranes prepared by blending PVA with poly(acrylic acid) (PAA) were tested for dehydrating fusel oil and by-product issued from Brazilian ethanol distilleries [98]. Sorption and permeation of aqueous alcohol (C1–C4) through PVA membrane crosslinked with a multifunctional crosslinker has also been reported [99]. Again, Alginate composite membranes, cross-linked with 1,6-hexanediamine (HDM) or PVA, were prepared by casting an aqueous solution of alginate and HDM or PVA on a hydrolyzed microporous polyacrylonitrile (PAN) membrane and characterized by PV separation of acetic acid-water mixtures [100]. Chung et al. have developed multilayer MMMs consisting of a selective MMM top layer, a porous poly (acrylonitrile-*co*-methyl acrylate) [poly (AN-*co*-MA)] intermediate layer and a polyphenylene sulfide (PPS) nonwoven fabrics substrate. The selective MMM layer was formed by incorporating KA zeolite in PVA matrix followed by crosslinking reaction of PVA with fumaric acid [101]. Thin, high flux and highly selective crosslinked PVA water selective layers have been prepared on top of hollow-fiber ceramic supports [102]. A hollow-fiber composite membrane, PVA-sodium alginate (SA) blend, supported by a polysulfone (PS) hollow-fiber UF membrane, was reported for pervaporative dehydration from binary aqueous solutions of isopropanol, n-butanol, tertiary butanol and ethanol [33].

Polyvinylamine (PVAm) is another variety of water soluble polymer possessing a number of primary amines in its backbone. However, the presence of intramolecular H-bonds generates high crystallinity, which makes the membranes brittle and less permeable [103]. A thin-film hydrogel composite was graft-polymerized on a polyethersulfone (PES) UF support employing the UV photo-initiation method with vinyl sulfonic acid (VSA) as the monomer and *N*,*N*′-methylenbisacrylamide (MBA) as the cross-linker monomer [104]. The optimal membrane exhibited a very high flux of 7.5 kg m^−2^ h^−1^ and SF of 313. However, the performances of the membrane decreased after 1–2 days of operation.

The limitation of PVA membranes in dehydration of corrosive liquids by PV was overcome by the development of membranes based on polycarboxylic acids [105,106,107,108]. The membrane made from polycarboxylic acids alone, like PAA, is too hygroscopic to impart the required mechanical integrity. Blend of inert polymers with polycarboxylic acids yields a membrane with good separation performances and mechanical strength. Thus, Huang et al. studied the dehydration of acetic acid using a membrane comprising of nylon-6-PAA crosslinked with a metal (Al(III)) ion [105]. However, such metal crosslinker is not stable and collapses with time. Hence, inert yet stable film forming polymers, like polypropylene and Teflon, were grafted into PAA, vinyl pyrrolidone, 2-hydroxy ethyl methacrylate (HEMA) by plasma grafting [106,107]. These membranes showed excellent mechanical stability with high selectivity for water, yet suffered from very poor flux. Copolymerization of polymerizable acid, ester or imide monomers with a monomer of an inert polymer gives rise to a membrane with consistent performances. On the basis of this fact, a number of copolymers, like poly(AN-*co*-AA), poly(AN-*co*-maleic anhydride) and poly(AN-*co*-HEMA) were synthesized and studied for dehydration for ethanol and acetic acid [108,109].

Bhat and Aminabhavi have done a thorough review on PV separation of SA and its modified membranes [110]. The survey indicated that SA based membranes have been reported to perform outstanding separation characteristics in dehydrating the aqueous-organic mixtures. PV membranes were prepared from SA-polyaniline (SA-PANi) polymer casted on ultra-porous PAN and PES supports for acetic acid dehydration [111]. In fact, PAN and PES supported SA-PANi composite membrane showed fluxes/selectivities of 0.07/441 and 0.04 kg m^−2^ h^−1^/359.3, respectively, at 2 wt % of water in feed mixture.

PANi, synthesized by oxidative polymerization and doped with PAA, was used for PV of aqueous isopropanol [112]. Polyimide membrane, crosslinked with tricarbohydrazide-1,3,5-benzenetricarboxylic acid trihydrazide (BTCH), was used for isopropanol dehydration [113]. Results showed that the highest SF of 3452 could be achieved with an optimal membrane forming temperature of 80 °C. A crosslinked polybenzoxazine membrane (CR-PBz-M) for dehydration of isopropanol has been studied [114]. When the membrane was swollen by isopropyl alcohol (IPA) solution, a change in micropore size distribution from a single distribution to a bimodal pattern was observed, which demonstrated an in situ self-promoted characteristic for pervaporative dehydration. The permeation flux of 330 kg m^−2^ h^−1^ and 100% of water in the permeate side has been recorded at 70 wt % IPA concentration. In this context, Kursun et al. developed a thermo-responsive poly(*N*-isopropyl acrylamide) (PNIPAAM) grafted PVA (i.e., PVA-*g*-PNIPAAM) membranes, suitable for separation of IPA-water mixtures [115]. At 87.40 wt % IPA in feed, the water flux and SF of such membrane was obtained to be 11 gm m^−2^ h^−1^ and 95, respectively.

The dehydration of corrosive organic solvents is always challenging. PV performance of crosslinked blend membranes of PVA and PAA had also been attempted for the dehydration of dimethylformamide (DMF) [116]. In another study, copolymers of acrylamide (AM) with increasing amounts of HEMA were synthesized and the crosslinked (gelled) copolymer membranes that were made from these sol copolymer solutions (uncrosslinked) were used for pervaporative dehydration of DMF within 0–13.07 wt % water in feed [117]. These hydrophilic gel copolymer membranes were found to be highly water selective in both sorption and diffusion through the membranes. Roy et al. studied the pervaporative dehydration of highly oxidizing hydrogen peroxide (H_2_O_2_). As the choice of membrane material is potentially limited for this system, perfluorodimethyldioxole–tetrafluoroethylene (PDD–TFE) copolymer membranes (CMS-3 and CMS-7) were used to concentrate H_2_O_2_ within 4–40 wt % of H_2_O_2_ at different temperature. In fact, the highest H_2_O_2_ selectivity of ~12 was observed for CMS-3 membrane at an H_2_O_2_ concentration of 43 wt % with a total flux of 6.15 × 10^−3^ gm cm^−2^ h^−1^ whereas for CMS-7, selectivity and total flux were found to be 9.2 and 9.6 × 10^−3^ gm cm^−2^ h^−1^, respectively, at the same H_2_O_2_ concentration. However, both the membranes showed long-term stability at 35 wt % H_2_O_2_ concentration [118]. Again, an aqueous pyridine solution was dehydrated using poly(AN-*co*-AA) membranes for separation of water-pyridine mixture [119].

A novel PV membrane material, synthesized from hyperbranched polyglycidol (HPG) and hyperbranched poly (amine-ester) (HPAE) followed by crosslinking terminal –OH with 4,4′-oxydiphthalic anhydride (ODPA) and GA, respectively, has been explored for pervaporative dehydration owing to the hydrophilicity [120]. Again, polybenzimidazole-polyetherimide (PBI-PEI) dual-layer hollow fiber membranes (HFMs) were used for pervaporative dehydration of ethylene glycol (EG) [121]. It was observed that an increase in operating temperature lowered the EG-water clusters and lower membrane-EG affinity of the membrane that improved the flux and selectivity. The water flux and SF was found to be as high as 186 g m^−2^ h^−1^ and 4500, respectively. In this context, Kujawski et al. studied the commercialized hydrophilic Pervap™ membranes (Pervap™ 2200, 2201, 2216, 2255, and 2510, supplied by Sulzer Chemtech AG, Winterthur, Switzerland) for dehydration of tetrafluoropropanol (TFP) aqueous mixtures [122]. It was observed that the *PSI* for Pervap™ 2200 and 2216 was ~5000 kg m^−2^ h^−1^, implying that both of the membranes can successfully be employed for the removal of water from TFP solutions.

The PV membranes were further modified with the incorporation of NMs into the polymer matrix. It was observed that introduction of NMs not only increased the mechanical strength of the membranes, but also influenced the separation characteristics significantly [123,124,125,126,127]. The favorable interactions between the NMs and the pristine polymer influence the membrane performances effectively. The NFs present in the membrane matrix may work as “spacers” to provide free spaces for water permeation through the polymer chains (e.g., silica, TiO_2_), or the molecular sieving effect (e.g., zeolite) stimulates water transport. CS based NCMs, synthesized by incorporating Preyssler type heteropolyacid, namely, H_14_[NaP_5_W_30_O_110_], NPs by solution casting and the solvent evaporation method exhibit a remarkable increase of SF of 35,991 from 96 base value for NCMs in comparison with unmodified membrane for the separation of ethanol-water mixtures [128]. The dehydration performances of CS and microporous titanosilicate ETS-10-CS MMMs were studied in the range 85–96 wt % ethanol [129]. It was observed that the permeate flux was increased from 0.45 to 0.55 kg m^−2^ h^−1^ at 50 °C for the ETS-10-CS MMM with respect to the pure CS membranes. García-Cruz et al. synthesized various CS based MMMs with different fillers, like room temperature ionic liquid [emim][OAc] (IL), metallic Sn powder, layered titanosilicate AM-4 and layered stannosilicate UZAR-S3, by solution casting [130]. The electrical conductivity and electrochemical response of the membrane, in strong alkaline medium, were measured. These thin CS-based MMMs (40–139 μm) were found to be highly alkaline resistant and exhibited higher conductivity than pure CS membranes. In fact, MMMs obtained via incorporation of Al-rich zeolite beta NPs into the SA matrix, followed by crosslinked with GA, has been tested for pervaporative dehydration and esterification of ethanol and acetic acid [131]. The hydrophilic nature of the Al-rich zeolite beta, along with its molecular sieving effect and favorable interaction with the polymer matrix, was found to be responsible for the relative enhancement of pervaporative dehydration.

Titanium oxide (TiO_2_) is one of the most common inorganic NFs that has been used widely in separation [132,133]. The incorporation of a small amount of TiO_2_ (0.25–1 wt %) modified with PANi into the GA crosslinked SA for the dehydration of 1,4-dioxane showed enhanced SF [134]. The surface modification of the NPs not only improved the dispersibility, but also increased its hydrophilicity. However, the reduction in membrane swelling decreased the flux significantly. Tancharernrat et al. fabricated styrene butadiene copolymer (SBR)–SiO_2_ composite membrane, where the obtained SBR–SiO_2_ NPs exhibited spherical morphology with SiO_2_ as the core and SBR as the shell via differential microemulsion polymerization for dehydration of alcohol [88]. In NCMs, the NPs offered extra free spaces to the water molecules, showed high water permeability, and increased permselectivity from its ethanol mixtures. Incorporation of microporous hydrophilic zeoliteT and aluminosilicate into crosslinked PVA and charcoal NPs into SA endorsed both water flux and SF for 1,4-dioxane dehydration [135,136,137]. The CS membrane was chemically modified by introducing an aromatic ring grafted with acidic –COOH (*N*-*p*-carboxy benzyl chitosan (NCBC)) and the cross-linked nanostructured NCBC-silica composite membranes were prepared for PV dehydration of alcohol mixtures [138].

In recent times, a novel technique has been applied to overcome the limitation of trade-off effects observed in PV by constructing a super-hydrophilic water uptake layer by spray-assisted bio-mineralization of calcium carbonate (CaCO_3_) onto a (poly(acrylic acid)/poly(ethyleneimine))n/polyacrylonitrile ((PAA/PEI)n/PAN) membrane [139]. The bio-mineralization dramatically increased the hydrophilicity (water contact angle decreased from 74° to 4.2°) and the membrane showed an enrichment of water content from 5 to 98.8 wt % while the permeate flux reached 1.3 kg m^−2^ h^−1^, which is almost five times that of the pristine membrane. Another technique was used to form a double network PVA (PVA-DN) NCM from interpenetration of two PVA networks of varying MW for PV dehydration. At first, the PVA network was created by crosslinking the high MW PVA (HPVA) in presence of silica nanospheres (SNSs) prior to a formation of the second network by the thermal crosslinking of low MW PVA (LPVA) [140].

The metal organic frameworks (MOFs), which are comprised of metal or metal clusters joined through organic linkages, have attracted significant attraction in separation fields [141]. The organic linkages present in MOFs provide higher compatibility of inorganic NMs in polymer matrices. The PV membranes fabricated from zeolite-type MOF NF has shown superior performances for dehydration of organics [142,143,144]. Zeolitic imidazolate frameworks (ZIFs), a sub-class of MOFs, display outstanding stability and compatibility within polymer matrix and exhibit desirable properties for various PV based applications [145,146,147,148,149,150,151]. The ZIF-7 crystal particles exhibit good interfacial adhesion when incorporated into the CS and the MMMs demonstrate higher flux and SFs at 2.5 wt % loadings [145]. However, higher loading of MOFs could lead to a crosslinking reaction between Zn atom of ZIF-7 and the amino groups of CS polymer, which eventually reduced the flux. The dehydration of ethanol has been studied by Amnuaypanich et al. using a semi-interpenetrating polymer network of natural rubber and crosslinked PVA incorporated with zeolite 4A [152]. The uniform porous structure of Zeolite 4A provided an alternative pathway to the water molecules that enhanced the selectivity and permeability. Silica NPs with –SO_3_H functionalization has been used for in situ of crosslinking CS to prepare CS-silica NCMs by Liu et al. [153]. The Hydrophilic 4A zeolite was also incorporated into the poly(ether-block-amide) (PEBA-2533) composite membranes up to the extent of 10–40 wt % of polymer weight to enhance separation properties for NMP-water mixtures. A high selectivity of 122 was observed at a reasonable flux of 55 g m^−2^ h^−1^ at 2.87 wt % of water [154]. The enhanced performance of hydrophilic zeolite 4A may be due to the presence of optimized average pore size of 0.4 nm, which is slightly bigger than the size of water molecule (i.e., 0.26 nm) [155].

Dudek et al. reported the PV dehydration from its alcohol mixture using CS and iron oxide NPs [156]. The in situ synthesized PVA-*g*-AN/HEMA-Fe_3_O_4_ NCMs has been employed for acetone-water system [157]. The addition of magnetite (Fe_3_O_4_) NMs effectively enhanced the water SF. The permeation fluxes and SFs were found to be within 0.015–0.091 kg m^−2^ h^−1^ and 29.1–14,000, respectively.

Polybenzimidazole (PBI) and PBI/ZIF-8 NCMs for pervaporative dehydration of alcohols have been studied [158]. The sorption and swelling studies showed that the high PV permeability of PBI/ZIF-8 NCMs is attributed to the high fractional free volume (FFV) created by large cavities of ZIF-8 particles. The water permeability of such membrane was found one order of magnitude higher than the original PBI membrane (14,000–22,000 vs. 1200–2300 Barrer) and there was a 29.2% reduction of energy barrier for penetrant transports across PBI membranes consisting of 33.7 wt % ZIF-8 NPs [146]. Liu et al. fabricated SA based MMMs via incorporation of kinds of ZIF (two-dimensional ZIF-L nanosheets and zero-dimensional ZIF-8 NPs) for PV dehydration of ethanol [159]. The water flux and SFs for ZIF-L-/ZIF-8-filled membranes were obtained of 1218 g m^−2^ h^−1^/1840 and 879 g m^−2^ h^−1^/678, respectively. The appropriate openings of ZIF-L nanosheets provided the desirable molecular sieving effect and rendered ordered water channels for rapid transport of water molecules.

Hua et al. utilized ZIF-90 NPs to fabricate NCMs with Matrimid^®^ polymer for IPA dehydration [148]. NCMs composed of phosphotungstic acid in SA for dehydration of alcohol by PV has been studied [160]. The incorporation of nanosized SA zeolite materials into the PVA matrix exhibited a fairly high water flux and reasonable SF for n-butanol-water system. Zeolites are microporous, crystalline alumino silicates that absorbs water molecules preferentially within its pores when the pore size matches the size of the water molecules [161]. The membrane filled with zeolite NPs offers higher water flux and selectivity compared to the pristine polymer membranes [162].

Recently, another group of material called polyhedral loigosilsesquioxane (POSS) has shown substantial potential as NFs in the NCMs due to its enhanced mechanical, thermal and oxidation resistance and compatibility with the membrane materials. NCMs, fabricated with several types of POSS including octa-anion (OA), octa-nitrophenyl (ONPS), octa-aminophenyl (OAPS) and octa-ammonium (OAS) into the CS matrix for ethanol dehydration, has been reported [163]. It was observed that the performances of these membranes are highly dependent on the specific interactions between the POSS and the membrane materials. The nano-size of the POSS materials (~1–3 nm) helps develop a dual-layer HFMs, where the POSS were embedded into the mixed matrix layer as a selective barrier for ethanol dehydration [164]. The incorporation of small amount of POSS NMs (1–2 wt %) into the selective layer of MMMs enhanced both permeation rate and SF, which may be due to the increased free volume and the diffusion selectivity. The highly hydrophilic crosslinked PVA-fullerenol membranes has been fabricated for dehydration of ethanol [165]. The incorporation of 5 wt % low-hydroxylated fullerenol C_60_(OH)_12_ and cross-linking with maleic acid helped for distributing the NPs uniformly into the amorphous PVA phase. Friebe et al. [166] synthesized 3D MOF structure, based on UiO-66 [Zr_6_O_4_(OH)_4_(bdc)_6_], featuring triangular pores of approximately 6 Å, as a thin supported membrane layer with high crystallographic orientation on ceramic α-Al_2_O_3_ for separation of H_2_ from different binary mixtures at room temperature having separation factors of H_2_/CO_2_ = 5.1, H_2_/N_2_ = 4.7, H_2_/CH_4_ = 12.9, H_2_/C_2_H_6_ = 22.4 and H_2_/C_3_H_8_ = 28.5. Again, Sun et al. [167] grafted PSBMA onto the surface of UiO-66-NH_2_ (UiO-66-PSBMA) via atom transfer radical polymerization (ATRP) procedure to suppress the aggregation tendency of raw MOFs. Moreover, Armstrong et al. [168] prepared UiO-66 impregnated PVCi to form cross-linked UiO-66 crystals, which was analyzed through powder X-ray diffraction, scanning electron microscopy, and Fourier transform infrared spectroscopy, and further characterized for potential uses as a gas membrane through inert gas permeation studies and nitrogen porosimetry.

As a new type of NFs, CNTs have also drawn substantial attention due to their unique structures and properties. The tubular structure of CNTs are made up of cylinders of graphite sheets of nanometer diameter. The pristine CNTs tend to agglomerate into bundles due to strong van der Waals attraction between the tubes. It was observed that the modification of CNTs led to better dispersion into the polymeric matrix and improved the separation performance of the multilayer MMMs. The NCMs, fabricated with CNTs, are extensively studied in PV for alcohol dehydration. The PVA-multi-walled CNT membrane showed an increase in water flux with the addition of CNTs loadings with unchanged SF up to 1 wt % [84]. The results revealed that the addition of CNTs alleviated the crystallinity present in the pristine membrane and stimulated the micro-orientation that eventually decrease the free volume of PVA membrane matrix. Although the presence of CNTs reduces the free volume, the overall flux increases as the CNTs offers an alternating faster diffusion path to the permeating molecules. The MMMs, comprising of CNTs and polymer polyelectrolyte complexes (PECs) as matrices, showed a uniform dispersion of CNTs in the membrane matrix. However, the cross-sectional SEM image showed significant damage of the CNTs probably as a result of the load transfer from PEC to CNTs under stretching [169]. In another study, multiwalled CNTs (MWCNTs) were functionalized with poly(3-hydroxybutyrate) (PHB) and then aligned into the CS matrix [170]. The presence of PHB improves the compatibility of the CNTs that helped ensure a uniform distribution into the matrix. The NCMs exhibited a relatively high flux and selectivity for water when employed for dehydration of 1,4-dioxane. The CS membrane was further modified with PVA-modified MWCNTs that employed in acetone dehydration. Further, the PVA functionalized MWCNT was bulk aligned on the poly(vinylidene fluoride) (PVDF) membrane by a simple filtration method and then coated with CS to form a novel three-layer NCM [171]. The novel three-layer CS-thin PVA-MWCNT-PVDF NCM exhibited a significant improvement in water flux with only a slight decrease in SF. In another work by Panahian et al., fabricated multilayer MMMs containing CNTs, PVA, PES and polyester as inorganic filler and selective top, intermediate and support layers, respectively, for dehydration of ethanol-water mixtures [172]. The incorporation of functionalized MWCNTs by diisobutyryl peroxide into CS membrane has been utilized for alcohol dehydration [173]. The NCMs, fabricated through CNTs incorporated into a separating layer of PVAm-PVA supported on a microporous PS substrate were used for dehydration of EG by PV [174]. The membrane containing 2 wt % CNTs exhibited a permeation flux of 146 g m^−2^ h^−1^ and a SF of 1160 at 1 wt % feed water concentration.

Recently, there has been much interest in graphene oxide (GO) as a material with unique electrical and optical properties. The distinctive structural features, high mechanical strength [175] and the atomic-level thickness of GO have been exploited to fabricate an extremely thin membrane with controlled pore size and high flux [176,177]. Potential applications of GO in water desalination and purification has also been explored [177,178]. The membrane fabricated by incorporating GO into the PVA matrix has been applied in the recovery of water from vinegar waste water. The presence of GO offered better hydrophilicity and showed a reasonably high water flux and SF [179].

In PV, a novel NCM, consisting of a cross-linkable 6FDA polyimide matrix and NH_3_ functionalized GO (i.e., NHGO) particles, has been studied for dehydration of alcohol. The membrane demonstrated a water permeability of 0.198 mg m^−1^ h^−1^ KPa^−1^ and a water-IPA molar selectivity of 6726 which was 35 times higher than that of the pristine-*co*-polyimide [180]. The Zwitterionic graphene oxide was incorporated into SA for efficient water-alcohol separation [181]. The GO surface was further modified with methylnicotinamide chloride (MNA) for oil-water separation [182] that improved the interaction between the nanosheets and the sulfonated polyphenylenesulfone (sPPSU) polymer. The aldehyde-functionalization of GO also improved the adhesion between the GO nanosheets [183]. The newly synthesized GO-framework (GOF) membranes possessed GO-aldehyde covalent bonds that helps to adjust microstructural properties. The alcohol dehydration performances of GOF membrane was much improved compared to pristine GO membrane. The water flux was obtained 2.59 kg m^−2^ h^−1^ for water-butanol dehydration and 99.7 wt % of water in permeate.

### 3.2. Removal of Organics from Aqueous Solution

Membranes prepared from organophilic polymers are generally employed for the removal of traces of organic from aqueous solution. The following polymeric membranes have been reported for this separation process.

Silicone containing polymers generally exhibit good organophilicity. Indeed, silicone based rubber membranes, mainly poly dimethyl siloxane (PDMS), have been the most investigated for separating many organic-water mixtures, such as alcohols, ketones, phenols, hydrocarbons and chloro-hydrocarbons, due to their exceptional wide-ranging performance in permselectivity, stability and production cost. Watson and Payne and Blumke et al. reported the sorption and permeation properties of organic compounds through PDMS membranes [184,185]. Netke et al. used a PDMS membrane for removal of isomeric picolines from its aqueous mixture [186]. To improve the organic selectivity of the PDMS membrane, hydrophobic adsorbent, like a molecular sieve, had been incorporated in the membrane and this filled membrane showed a higher SF than the unfilled one when applied for pervaporative removal of an organic from its aqueous solution [187,188]. Netke et al. filled PDMS membrane with hydrophobic silicalite filler but selectivity for acetic acid for aqueous acetic acid mixture was poor for such membrane [189]. The polyoctylmethyl siloxane (POMS) membrane has been considered to separate acetone-butanol-ethanol from aqueous solutions [190]. The effect of multicomponent feed systems, such as addition of acetone and ethanol in feed, on the PV performance of the membrane were analyzed. When applied in a real fermentation broth, the membrane showed high selectivity of 9.8, 3.8 and 0.9, for butanol, acetone and ethanol, respectively.

A series of unsaturated silicone backbone containing polypropynes, like polytrimethyl-silylpropyne (PTMSP), were tried for selective alcohol separation from its aqueous solution [191]. These membranes were found to yield very high permeation rate. Slater et al. studied PV performance of a series of alcohols, like ethanol, n-butanol and tert-butanol, through PDMS and PTMSP and polymethoxy siloxane (PMS) membrane [192]. Volkov et al. investigated the PV separation of ethanol and acetone from their aqueous solution through polyvinyltrimethyl silane (PVTMS), PVTMS-PDMS block copolymers, PVTMS-polybutadiene (PB) block copolymers and other modified PTMSP membranes [193]. Ethanol-permselective membranes were prepared from various silicone and silane monomers under extremely mild polymerization conditions. These membranes have polydimethylsiloxane-like structures and SFs of 1.5–5.2 [194]. Pervaporative separation of aqueous solutions of propionic, butyric and iso-butyric acid has been studied using plain and filled silicone rubber membranes [195]. A novel silicone rubber membrane was prepared by crosslinking silylstyrene-oligomer containing -SiH groups with divinyl-polydimethylsiloxane using Karstedt’s catalyst at room temperature for separation of organics from water [196]. In order to stabilize the production of highly concentrated ethanol, a coupled fermentation/PV process using ethanol permselective silicalite membranes coated with silicone rubber was studied [197].

Non-silicone synthetic membranes have also been reported in recent years. The most investigated is polyetherimide block polymer (PEBA) by Boddeker for extraction of alcohol and hydrocarbon (aromatic) [198]. This membrane showed good selectivity for high boiling bio-products. Matsumoto et al. reported phenol removal from its aqueous solution using the same membrane [199]. It was observed that the diffusion rate of phenol in PV mixtures was not affected by the presence of water. In fact, pervaporative removal of halogenated hydrocarbons, from waste streams, through poly(bis-phenoxy phosphazen) membrane was investigated by Peterson et al. [200]. The membrane showed high SF for these hydrocarbons. Nakagawa et al. studied the PV enrichment of chlorine containing hydrocarbons (1,1,2-trichloroethane and tetrachloroethane) from dilute solutions through poly(acrylate-*co*-AA) composite membranes crosslinked with *N*,*N*,*N*/,*N*/-tetra glycidyl metaxylenediamine [201]. High SF and acceptable permeation rate for the hydrocarbons were obtained. For the removal of aniline, phenol, nitrobenzene and some other high boiling solvents from process water and wastewater, copolyimides have been synthesized and modified in order to obtain organophilic membranes [202]. Removal of VOCs, such as benzene and chloroform, from aqueous mixtures, through poly(methylmethacrylate)-PDMS (PMMA-*g*-PDMS), poly(ethylmethacrylate)-PDMS (PEMA-*g*-PDMS), and poly(n-butylmethacrylate)-PDMS (PBMA-*g*-PDMS) graft copolymer membranes, were investigated by PV [203]. A highly benzene selective membrane has been synthesized by the addition of hydrophobic IL, 1-allyl-3-butylimidazilium bis (trifluoromethane sulfonyl) imide ([ABIM]TFSI) to the poly(methyl methacrylate)-graft-poly(dimethylsiloxane) (PMMA-*g*-PDMS) membranes [204]. The membrane showed high benzene permselectivity when applied for the removal of a trace amount of benzene (0.05 wt %) in water.

A novel asymmetric ceramic-supported polymer (CSP) PV membrane was developed using free-radical graft polymerization of polyvinyl acetate (PVAc) onto a porous tubular silica substrate. The resulting membrane was characterized by PV removal of trichloroethylene (TCE) and chloroform from dilute aqueous solutions [205]. An in-depth investigation of integral asymmetric poly(vinylidene fluoride) (PVDF) membranes has been carried out for the extraction of polar and non-polar organic compounds from dilute organic-in-water feed solutions [206]. The pervaporative separation of ethanol-water mixtures utilizing composite membranes, prepared by coating a thin film of a polystyrenesulfonate across the surface of a microporous alumina support, was also investigated [207]. A novel composite flat-sheet membrane with a dense SBS top-layer, coated on Fluoroplast F-42 support with an intermediate layer of PDMS and PU layer [208] has been utilized for recovery of ethanol from its aqueous mixtures.

Hosseini et al. prepared a hydrophobic composite membrane of PDMS-poly(methyl hydrogen siloxane) (PDMS-PMHS) membrane for the pollution control and solvent recovery of dimethylsulfoxide (DMSO) from its aqueous solution [209]. At 10 wt % feed concentration, SF was found to be 57. The PV performance was also observed to be dependent on operating temperature. In fact, the DMSO flux was found to increase from 0.386 to 0.565 kg m^−2^ h^−1^ with an increase in the temperature from 25 to 70 °C. PV separation of methanol from aqueous solutions with PVA based hybrid membranes by Hu et al. showed a flux of 0.9–4.0 kg m^−2^ h^−1^ and selectivities within 1.04–1.63 [210]. The methanol flux/SF for Sulzer PERVAP™ 4060 and 2211 membranes were 0.9–3.2 kg m^−2^ h^−1^/2.6–7.8 and 4.3–8.9 kg m^−2^ h^−1^/1.1–2.0, respectively, at 0.05–20 wt % of feed methanol concentrations within 50–70 °C [211]. Pervaporative recovery of VOCs from the methanol-containing binary, ternary and quaternary industrial waste water solutions by vinyltriethoxysilane (VTES)-grafted-silicalite-1-PDMS MMMs has been investigated [212]. At 65 °C, the maximum *PSI* of 5346 g m^−2^ h^−1^ with SF of over 10 were obtained at 10.5 wt % feed methanol concentration. Uragami et al. investigated the removal of traces of VOCs, such as chloroform, benzene and toluene, from aqueous solutions, using the poly(styrene)-*b*-PDMS (PSt-*b*-PDMS) membranes containing an IL, 1-allyl-3-butylimidazilium bis (trifluoromethane sulfonyl) imide ([ABIM]TFSI) ([ABIM]TFSI-PSt-*b*-PDMS) by PV [213]. As the VOCs are preferentially attracted by the IL, both the permeability and the selectivity increased with increase in IL content.

The incorporation of suitable NMs was also observed to enhance the separation and permeation performances of the MMMs. Davey et al. thoroughly discussed the fabrication of various novel MMMs for fermentative separations [214]. The review showed that MMMs are currently delivering membranes with enhanced performances for these separations. Vane et al. extensively studied the various factors affecting the alcohol-water PV performance of hydrophobic zeolite-silicone rubber MMMs [215]. The membrane exhibited ethanol-water PV permselectivities up to five times to that of silicone rubber alone and three times higher than simple vapor–liquid equilibrium (VLE). For removal and recovery of organic solvents, ZSM-5 zeolite with a high ratio of Si:Al and silicalite-1 (an aluminum-free ZSM-5) has been used as the mostly common inorganic fillers. The MMMs fabricated by incorporating the high-silica ZSM-5 molecular sieve particles into the silicone rubber matrix showed enhanced ethanol recovery from its aqueous mixtures [216]. It was found that the small particle size, uniform dispersion and loading amounts are the most important parameters to achieve the desired membrane properties. However, the membrane showed a reduction in its performance over time that may be due to the swelling of the membrane. The membranes also showed declined performances when applied for the recovery of the butanol from fermentation broths. The other products present in the broth may be adsorbed within the zeolite particles, reducing the butanol permeation rate and SF. Further, the chemical modification of the ZSM-5 zeolite particles, via hydrofluoric (HF) acid etching, removes the organic impurities and improves the surface hydrophobicity and roughness. The chemically treated NPs showed better alcohol separation than the unmodified NPs when embedded into PDMS membrane [217,218]. Such modification also improved the interfacial adhesion. PEBA membranes fabricated with silicalite NPs showed an improvement in flux and SF for ethanol recovery up to 2 wt % loadings then the performance declined due to the transport resistance of adsorbed molecules [219]. The strong affinity exhibited by the butanol molecules towards the silicalite could be useful for in situ butanol removal from fermentation broths. The silicalite NPs were further modified by silylation with vinyltriethoxysilane (VTES) that improved the interfacial interactions between the PDMS matrices [220] and reduced the void formation and membrane swelling on prolonged use. The modification allowed high loading of NPs up to 67 wt % into the membrane matrix that eventually increased the selectivity of the membrane. Liu et al. fabricated an ultra-thin highly homogeneous nanosilicalite-PDMS active layer on a porous alumina capillary support [221] for the recovery of iso-butanol from aqueous solutions (0.2–3 wt %). The membrane exhibited flux and SF as high as 11.2 kg m^−2^ h^−1^ and 41.6, respectively. For ethanol recovery, Huang et al. synthesized a novel MMMs consisting of a polyphosphazene nanotube (PZSNT) embedded in a PDMS matrix [222]. The enhanced separation performance in the presence of PZSNT is mainly due to the strong affinity towards ethanol, compatibility with polymer matrix and reduced diffusion resistance. The nanotubes with smaller diameter showed better flux and SF as the interface surface increased. The hydrophobic NCMs synthesized by incorporating surface-functionalized fumed silica in polydimethylsiloxane (PDMS) for recovery of 1-butanol from its aqueous solutions [223]. The membrane demonstrated improved butanol permeability and selectivity. Introduction of superhydrophobic surfaces using SiO_2_ and PDMS decreased the affinity of the membrane surface towards water molecules and was found to be useful for separation of ethanol from its aqueous mixtures [224].

The introduction of polyhedral loigosilsesquioxane (POSS) has improved the organic compound recovery from its aqueous mixtures. The –OH present in the POSS materials may interact with the polar functional groups present in the polymer chains. Octa(3-hydroxy-3-methylbutyldimethylsiloxy) POSS (AL0136) and disilanolisobutyl POSS (SO1440) NPs were used with Pebax membrane showed enhanced organic recovery [225]. The MMMs, fabricated using hydrophobic nanosilica into poly(1-(trimethylsilyl)-1-propyne) (PTMSP), showed significant enhancement in permeation flux for pervaporative separation of dilute ethanol and butanol mixtures (5 wt %) from its aqueous solutions [226]. The initial data at 50 °C showed a flux of 9.5 kg m^−2^ h^−1^ with SF of 18.3 for ethanol-water separation and 104 for butanol-water separation, respectively, which may decline with time because of aging of the membrane. The use of ZIF in solvent recovery has been investigated [227] by Liu et al. where ZIF-71 has been incorporated into Pebax membrane. The organic nature of the super-hydrophobic ZIF-71 helps disperse uniformly. The MMMs were successfully employed to remove n-butanol from acetone-butanol-ethanol (ABE) solution. At 20 wt % NMs loadings, the MMMs demonstrated a total flux of 520 g m^−2^ h^−1^ and SF of 18.8 at 37 °C. The membranes were observed to be highly stable up to 100 h of operation with real ABE solution obtained from fermentation broth. Free-standing ZIF-71-PDMS based NCMs have been reported for pervaporative separation of ethanol and 1-butanol from its aqueous mixture [228]. For recovery of the aroma, isopropyl acetate from its aqueous solutions, ZSM-5 filled hydroxyl terminated polybutadiene (HTPB)-based polyurethaneurea (PU) membranes [229] have been studied. A homogeneous ZIF-8-silicone rubber NCM with high particle loading has been synthesized by Liu et al. [230] on a hierarchically ordered stainless-steel-mesh (HOSSM) employing a novel “Plugging–Filling” method. The membrane showed a very high SF of 53.3 and total flux 0.90 kg m^−2^ h^−1^ at 80 °C for recovery of furfural (1.0 wt %) from water. From the literature review, it was observed that the incorporation of NMs not only resolves the problems associated with the mechanical stability of the membranes under prolonged use in an organic environment, but also improves the SF without affecting the overall flux.

### 3.3. Organic-Organic Separation

It is very difficult to find a suitable membrane for potential organic-organic separation. The polymers chosen for membrane preparation are based on their relative SPs with respect to the permeants to be separated. This SP approach plays the main role in imparting the separation efficiency of the membrane when the permeants are of same size and mass resulting in little difference in their relative rate of diffusion through the membrane.

Several attempts have been made for selective pervaporative separation of isomeric xylenes [231] through membranes comprising of polyethylene, poly(vinylidine fluoride) (PVF) along with various Werner complexes, like hydroxy propyl methyl cellulose having α-cyclodextrin cellulose esters and poly(p-xylene) film. McCandless investigated separation of benzene-cyclohexane mixture by PV using PVF membrane and obtained the SF as high as 20 [232]. DMF and DMSO were added in the feed to enhance the permeation rate but SF was found to decrease to 6. Cabasso examined the separation of these mixtures through polymeric alloy membrane of various compositions of polyphosphonates [204]. An SF of 13 for benzene was obtained with this membrane, and DC for benzene was found to depend on its concentration in the polymer matrix. Among the other systems, separation of methanol from anhydrous organic mixtures has been of growing interest, as its removal is required in many situations. In most of these cases, methanol forms azeotropes with the other organic compounds like alkanes, ethers and esters. Several works have reported on the separation of aromatic-alcohol, alkanes-alcohol, alcohol-ether, etc. [233,234,235,236,237,238,239,240]. Separation of methanol or ethanol from its mixture with MTBE, dimethyl carbonates (DMC), etc., have been reported [239,241,242,243,244]. The MTBE, ethyl tertiary butyl ether (ETBE), tertiary amyl methyl ether (TAME) etc. are being extensively used as lead free octane enhancers. These ethers are manufactured by reacting an isoalkene, e.g., isobutene for MTBE, with excess methanol over a sulphonic ion exchange resin. In recent year cellulose acetate membrane has already been commercialized for pervaporative separation of these ethers from methanol [243]. Considering the similar nature of methanol and water, hydrophilic membranes are used for separation of methanol from these ethers. Volkov et al. studied the effects of synthesis conditions, molecular mass characteristics and chain microstructure on the properties of poly[(1-trimethylsilyl)-1-propyne] based membranes for PV recovery of organic products from fermentation broths for the preparation of bioethanol and biobutanol [245]. The membranes exhibited good chemical resistance under the conditions of separation of fermentation broths.

Pervaporative separation of toluene-methanol mixtures over the entire range of concentration were investigated using both the un-grafted poly(ethylene terephthalate) (PET) and the grafted PET-*g*-PST membranes [244]. It was found that PET-*g*-PST membranes exhibited better toluene selectivity than the un-grafted PET membrane while the permeation fluxes of the grafted membranes were lower. Grafted PET-*g*-PST membranes with degrees of grafting up to 35% were found to be better than the un-grafted PET for PV of toluene-methanol system. The separation of benzene-cyclohexane mixtures and xylene isomer mixtures through a fixed-carrier membrane consisting of cellulose acetate (CA) as a base polymer and dinitrophenyl (DNP) group as a selective fixed-carrier were studied by Mitsuyoshi Kameda et al. [246]. Crosslinked oligosilylstyrene–polydimethylsiloxane composite membranes were used to separate traces of 1,2-dichloroethane (1,2-DCE) from its mixtures by PV and studied the effect of flow rates on PV performances [247]. Polyurethane (PU) dense membranes were used in the separation of binary and multicomponent aromatic-aliphatic mixtures by PV [248]. Poly(hexamethylene sebacate) (PHS) which has strong affinity for styrene was selected as membrane material, and the characteristics of permeation and separation for the styrene-ethylbenzene mixtures through these PHS cross-linked with *N*,*N*,*N*′,*N*′-tetraglycidyl m-xylenediamine(TETRA-DX) membrane by PV were also investigated [249]. Conducting polymer composite membranes with a separation layer of polypyrrole doped with hexafluorophosphate (PF_6_^−^) and p-toluenesulfonate (CH_3_C_6_H_4_SO_3_^−^), were examined for the removal of methanol from organic solvents, like toluene, IPA, MTBE and acetonitrile, by PV [250]. The thin plasma polymerized AA films on poly(3-hydroxybutyrate) (PHB) membranes were fabricated and studied for methanol-MTBE separation [251]. The experimental results showed that the fluxes were relatively lower than the pristine membrane. However, *PSI* was always higher. The permeability was found to be within the 660–4045 and 10.5–57.7 Barrer for methanol and MTBE, respectively. PVA membrane was investigated for separation of IPA from its toluene mixtures [252] for feeds composition from 10 to 40 wt % toluene at 35–50 °C and 4–16 psi. The PV experimental results showed that at an optimum 12 psi applied pressure, 40 °C temperature and 10 wt % toluene in feed solution, the toluene content of 78 wt % in the permeate was obtained. Ribeiro et al. fabricated a series of aromatic polyimides and polybenzoxazoles and evaluated them as membrane materials for separation of aromatic-aliphatic mixtures by PV [253]. The high temperature PV experiments at 80 °C showed an enhancement of aromatic hydrocarbon permeability approximately four orders of magnitude depending on the chemical structure of the diamine.

In a PV study of separation of toluene-methanol mixtures, Ray et al. efficiently crosslinked the SBR and NR membrane and compounded with different doses of reinforcing carbon black fillers of high surface area [70]. The improved selectivity was achieved through chemical crosslinking by efficient vulcanization and physical crosslinking through filler incorporation. The incorporation of fillers not only enhanced the aromatic selectivity of the membranes but also improves the swelling resistance and mechanical strength of the soft rubber membranes.

Compared to other two PV separations, separation of organics using NCMs are less common. The research studies have mainly focused on the separation of alcohol from other organic mixtures, or separation of isomers and aromatic mixtures. The NPs present in the matrix mainly serve two major advantages, viz. (i) reduce membrane swelling or plasticization effect and (ii) offers large surface area for sorption and transport. In most cases, the overall flux and the selectivity showed a trade-off relationship. Researches in this field have shown that the incorporation of suitable NMs not only increase the mechanical strength and long-term stability of the membranes, but also enhances the selectivity of particular components. The stability of MFI zeolite-filled PDMS membranes during pervaporative ethanol recovery from aqueous mixtures containing acetic acid has been studied [254]. It was observed that longer-term exposure to acetic acid resulted in an irreversible, steady decline in ethanol-water SF due to decreasing ethanol flux. The decrease in ethanol flux is probably attributable to the interaction of acetic acid with the silanol groups on the external surfaces of the zeolite particles or with the PDMS in the polymer-zeolite interface, which eventually restricts the ethanol transport into the zeolite pores. PV separation of toluene-alcohol mixtures using silicalite zeolite embedded CS MMMs has been studied [255]. The membrane permeated toluene preferentially with the selectivities/fluxes of 264/0.019–0.027 and 301/0.019–0.026 kg m^−2^ h^−1^ for toluene-methanol and toluene-ethanol mixtures, respectively. The overall PV performance was increased with increasing silicalite content in the MMMs. PDMS membrane filled with ZSM-5 zeolite filler has been successfully used for the separation of 1-butanol-2,3-butanediol mixtures [256]. The enhanced separation performance of the filled membranes was ascribed to the positive filler–polymer interactions, and the improved mass transfer contribution of surface flow through the zeolite pores. M. Tamaddondar et al. synthesized self-assembled polyelectrolyte surfactant (PELSC) NCMs for PV separation of MeOH/MTBE [257]. The separation of aromatic-alkanes was carried out using composite PVC membranes containing up to 40 wt % activated carbon (Maxsorb SPD30) [258]. The performances of the NCMs were significantly increased compared to pristine membrane, showing an enhancement of flux seven times higher and slight reduction in selectivity.

CNT-incorporated membranes are also extensively studied in PV for organic-organic separations. The PV separation of benzene-cyclohexane mixtures has been studied by Peng et al. using PVA membranes modified with CS-wrapped MWCNTs [259]. The membrane exhibited an increase in both permeation flux and SF. The incorporation of CNTs could provide the internal nano-channel at the polymer interface for selective permeation. MWCNTs functionalized with isonicotinic acid followed by grafting with Ag^+^ was incorporated into the CS membrane and used for the separation of benzene-cyclohexane mixtures [260]. The modified membrane was observed to absorb more benzene with an increase in the content of the MWNTs-Ag^+^ in the membrane and the benzene content in the feed mixtures. The benzene flux and selectivity was increased up to 357.96 kg m^−2^ h^−1^ and 7.89, respectively, at 1.5 wt % MWNTs-Ag^+^, 50 wt % benzene concentration and 20 °C. The PAm membrane, in the same way, fabricated with 2 wt % CNTs showed high selectivity and permeability for pervaporative separation of MeOH/MTBE mixtures [261].

Researchers often used transition metal ions (M(I/II)), such as Ag(I), Cu(I), and Co(II), as the facilitated transport fillers in the membranes for separating aromatic-aliphatic mixtures [262,263]. Wu et al. fabricated AgCl-poly(GMA-*co*-MMA-*co*-AMPS) copolymer hybrid membranes by in situ microemulsion polymerization for the separation of benzene-cyclohexane mixtures [264]. The pi-complex interaction forms between Ag^+^ and the double bonds in aromatic molecules, enhanced the aromatic compound separation ability. However, the incorporation of Co(II) into PVA matrix showed significantly high permeation and SF of 150 g m^−2^ h^−1^ and 60, respectively [263]. These metal ions interact with the permeating species and facilitate the selective transport of the corresponding species. However, the metal ion facilitated membranes showed lack of stability and loss of M(I/II) in the membrane matrix during the separation process has been observed [265].

On the other hand, the NCMs, fabricated by using MOFs, built by M(I/II) and organic ligands, exhibit higher design ability in composition and improved stability. Co(II)-formate doped into poly(ether-block-amide) (PEBA) membrane has been demonstrated feasible to be used as stable aromatic hydrocarbon transport carrier for facilitating aromatic hydrocarbon transport membranes [266]. The membranes showed a very high permeate flux of 771 g m^−2^ h^−1^ with a SF of 5.1 at 10 wt % toluene-n-heptane mixtures. The IL membrane prepared via coating BMIM^+^BF_4_^−^-Cu nanocomposite dispersions onto a polyester microporous membrane support by Kim et al. for the separation of propylene-propane mixture showed enhanced separation performances compared to pristine membrane [267].

The separation of aromatic compounds from aliphatic mixtures is quite challenging. A novel graphite-filled PVA-CS hybrid membrane for PV of benzene-cyclohexane mixtures has been reported [268]. It was found that both of the permeation flux and SF improved with incorporation of graphite. The presence of specific *σ*- and π-bond interactions between graphite and benzene improved the separation performance. Li et al. used an atmospheric dielectric-barrier-discharge plasma graft-filling technique to graft the copolymers in the sublayer pores and onto the surface of an asymmetric PAN based UF membrane [269]. To enhance the stability of the PV membrane for aromatic-aliphatic separation, Wang et al. fabricated a “pore-filling” membrane by dynamic pressure-driven assembly of a PVA-GO nanohybrid layer onto an asymmetric PAN UF membrane [270]. The pore-filling structure efficiently reduced the membrane swelling and the increased affinity of the membrane to aromatic compounds due to the molecular-level dispersion of GO in PVA led to improved performance in the PV separation of toluene-n-heptane mixtures. For separation of benzene from its cyclohexane mixtures a NCM has been fabricated by incorporating three-dimensional silver-GO into polyimide hybrid membranes [271]. The hybrid polyimide membrane showed highest SF reached to 35 at 15 mass% of Ag-GO loadings. An increase in Ag content in Ag-GO samples formed Ag NPs on GO surface through impregnation reduction reaction, which eventually enhanced the separation performances. It is thus clear that the key points of separation and purification of organic liquid mixtures to be feasible in terms of both technically and economically are the design and construction of both physical and chemical structures of the NCMs for enhanced membrane performances.

## 4. PV membranes Fabrication Techniques

In PV, the membranes play the most important role in determining the technological and economical efficiency of the aforementioned technology; and the improvement in membrane design could greatly affect the performance of current technology. To this point, most of the membrane materials used in PV technique are suitable for small-scale laboratory research purposes, but not in large-scale industrial applications. It is thus important to investigate on the fabrication techniques of more membrane materials to overcome the weaknesses in existing membranes. Different fabrication techniques used for the preparation of PV membranes are discussed below.

### 4.1. Solution Casting Method

**Figure 1:**
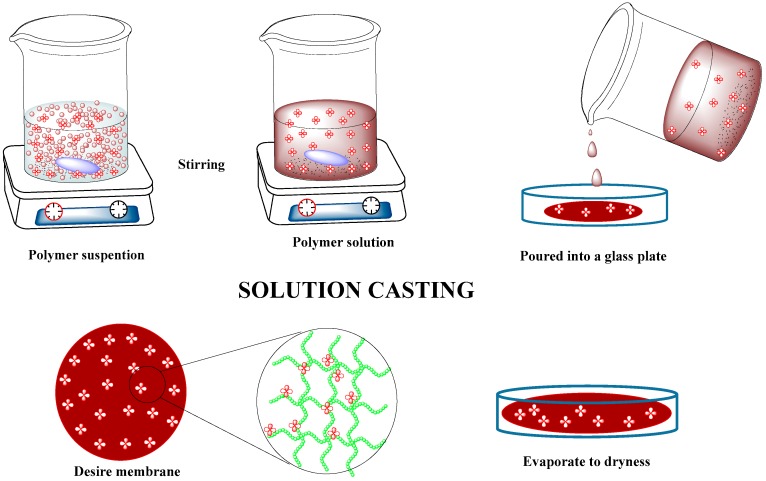
Preparation of Nanocomposite Membrane (NCM) by solution casting method.

Solution casting method is frequently used to synthesize composite PV membranes. In this process, the polymer and all other necessary ingredients are dissolved in a common solvent to obtain a homogeneous solution, followed by the casting of this solution onto a flat glass plate or Petri dish, as shown in Figure 1. After complete evaporation of solvent, the dried membrane is then peeled off from the glass support. Solution casting method is not only suitable for single-layered membrane formation, but also highly useful for the fabrication of multi-layered dense and/or porous membranes through the multi-solution coatings on the glass support. In fact, introduction of highly volatile solvents to the casting solution with an evaporation step prior to the phase inversion in a non-solvent bath can encourage the formation of top dense layer. The incorporation of NMs into the polymer solution requires special attention, as the NPs always tend to agglomerate that result in poor dispersion and inferior membrane performances. The solution prepared by mixing the polymer, solvent and the NMs often stirred for long time (usually 8–12 h) vigorously and/or exposed to ultrasonication. Solution casting method has extensively been studied for fabricating various types of nanocomposite PV membranes [123,146,153,162,272,273,274,275]. Gao et al. fabricate the PV membranes by solution casting technique for dehydration of IPA using the novel PVA NCMs, containing zeolites of several metal ions, like Na^+^, K^+^ and Ca(II) [272]. Additionally, another two new PVA based NCMs were reported by Mali et al., via incorporating phosphomolybdic acid and silicate NPs, using the solution casting method [273]. In succession, Adoor et al. also investigated the pervaporative dehydration of IPA- and ethanol-water mixtures using filler incorporated SA membranes, incorporating pristine and modified pristine NPs [162]. On the other hand, Li et al. and Prasad et al. reported the use of zeolite 13X NPs into the polyimide and sodium carboxymethylcellulose membranes, respectively, to fabricate novel NCMs for pervaporative dehydration of IPA-/ethanol-water mixtures [274,275]. In all of these studies, substantial variation of separation potential, as reflected from the reasonable variation of both flux and SF, has been noted. Weng et al. [276] utilized the knife casting method for synthesizing a multi-walled carbon nanotube/PBNPI nanocomposite membrane, perfectly suitable for H_2_/CH_4_ separation, whereas Bae et al. [277] prepared TiO_2_ nanoparticle incorporated sulfonated polyethersulfone (SPES) membrane through the knife casting method. Again, Vatanpour et al. [278] and Solè et al. [279] used the phase-inversion method for synthesizing polyethersulfon-TiO_2_ nanocomposite membrane and Sorbitan monolaurate [Span20] incorporated polyoxyethylene sorbitan monolaurate [Tween20], polyoxyethylene(4) lauryl ether [C12E4] membrane, respectively via the phase-inversion method.

### 4.2. Solution Coating Method

**Figure 2:**
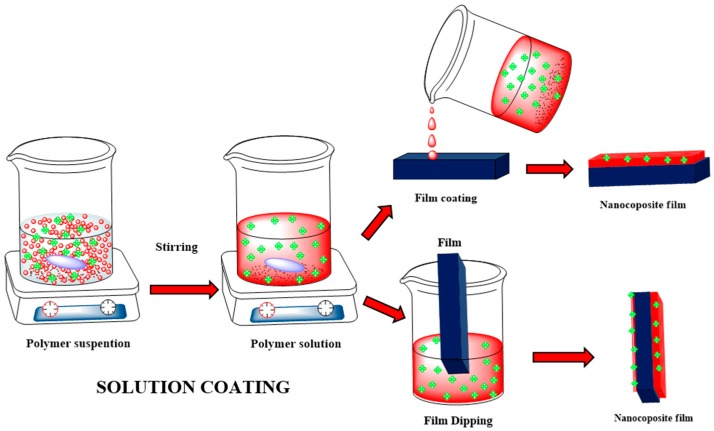
Fabrication of NCM by solution coating method.

Another frequently used technique for fabricating thin composite membranes is solution coating. This technique is normally used to synthesize composite membranes, via depositing a thin selective layer, on top of a microporous substrate or support, which may adopt flat-sheet, hollow fiber or tubular shape (Figure 2). In fact, the degree of porosity of the substrate should be sufficiently high so that the membrane resistance can mainly be controlled by the coated selective layer [280,281]. In this context, the pore size distribution of the substrate surface should preferentially be sharp and free from large defects, so that the intrusion of coating solution can be prevented. In fact, pre-wetting of the substrate with a low boiling point solvent, which is immiscible with the coating solvent, prior to the coating process can minimize such intrusion [280,281]. The pre-wetting solvent is then evaporated to obtain the coated membrane.

Spin coating is another procedure used to fabricate uniform thin films. In this process, a small amount of polymer-NM dispersion is applied on the center of the substrate that is spinning at desirable speed. Several researchers utilized this spin coating technique to fabricate uniform improved NCMs. Ding et al. improved the permselectivity of PV membrane by constructing the active layer through alternative self-assembly and spin-coating [282]. A high PV dehydration performance has been observed for the composite membrane fabricated with an ultrathin alginate/PAA-Fe_3_O_4_ active layer fabricated via spin coating [283]. In this context, Wang et al. [284] prepared a nanocomposite membrane via thin coating of PVA membrane on the multiwalled carbon nanotube. Again, Steele et al. [285] prepared superoleophobic coatings of ZnO nanoparticles blended with a waterborne perfluoroacrylic polymer emulsion membrane.

### 4.3. Blending of Solutions

One of the easiest ways to fabricate nanocomposite hybrid membrane is the direct mixing of inorganic NPs into the previously prepared polymer matrix. Such mixing/blending can be carried out in two ways: (i) in solution blending, where both components (i.e., membrane and NPs) are dispersed in a common solvent and (ii) in melt blending, where both components are dispersed as melt at high temperature. Of these two, melt blending is more common owing to higher efficiency and environmental inhibition. Dispersion of NPs in a polymer matrix usually led to undesirable agglomeration. Surface modification techniques, such as irradiation grafting, have been used to modify the NPs and break down its agglomerates in producing nanostructural composites. In this context, Genne et al. prepared PS-ZrO_2_ NCMs using 18 wt % PSF solution in *N*-methylpyrrolidone (NMP) with different amounts of ZrO_2_ NPs [286]. The membrane permeability increased as the amount of ZrO_2_ weight fraction was increased. Wara et al. reported the fabrication of NCMs of cellulose-Al_2_O_3_ by using the solution blending [287]. Liquid-state blending at the molecular level is widely used to prepare hybrids, which minimize the limitations of melt blending. During the past decade, fabrication of polymer-silica NCMs by solution casting mixtures of nanosilica has received much attention because of their easy and convenient preparation (Table 1). Such membranes were studied for gas separation, PV and PEMs in fuel cell applications [288,289].

Blending polymerization is an easy method to operate and suitable for all kinds of inorganic materials. Also, the concentration proportions of the components, i.e., polymer and inorganic components are easy to handle and thus, used frequently. However, the inorganic ingredients are liable to aggregate in the membranes, resulting in an inapplicable membrane formation [296,297,298].

### 4.4. Hollow Fiber Spinning (HFS) Technique

Different types of membranes, like flat sheet composite, polymeric hollow fibers and inorganic tubular membranes, are gaining high commercial interest, owing to their potential application in RO, micro-/nano-/ultra-filtration (MF/NF/UF), dialysis, GS and PV. Of these synthetic membranes, hollow fiber polymeric membranes, first invented by Mahon in 1966 [299], have shown tantamount impact and utmost importance, since these possess several advantageous attributes. In fact, high demand of HFMs, as compared to the other membranes, is associated with higher packing density and higher effective membrane area per unit volume of the separation device (i.e., membrane module), resulting in greater process intensification. Furthermore, the HFMs provide significant self-mechanical support and ease of handling during module fabrication and process operation. In this context, Sukitpaneenit and Chung reported the use of PVDF-nanosilica dual-layer HFMs, synthesized through HFS method, for the pervaporative recovery of ethanol from the binary aqueous solution with high permeation flux and organic SF [300].

The majority of innovations and modifications towards HFMs synthesis have been carried out through trial and error methods, aided by past experience, empirical data and qualitative scientific understanding. In fact, the design of such membrane technology has emerged to provide sustainable solutions of global demand for clean energy, water and health management. In order to fabricate novel HFMs, possessing high pervaporative separation potential, researchers have to investigate the intrinsic physicochemical properties of the membranes, manipulate phase inversion processes and control dope rheological responses during membrane formation.

**Figure 3:**
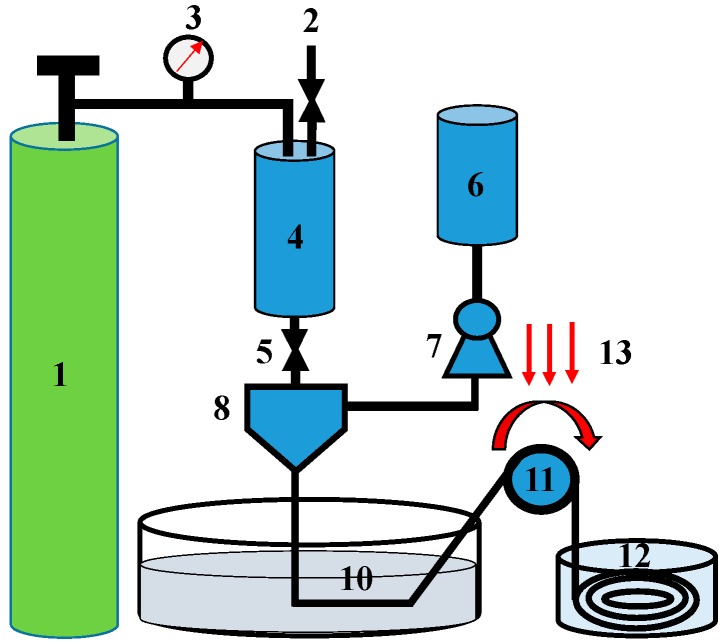
Typical hollow fiber spinning system: 1. spinning dope tank, 2. regulating pressure valve, 3. pressure gauge, 4. dope vessel, 5. dope valve, 6. bore liquid vessel, 7. bore liquid pump, 8. spinneret, 9. air gap, 10. Coagulation bath, 11. windup drum, 12. fiber collecting reservoir and 13. wash water.

Synthesis of HFMs, comprising of both desirable morphology and separation performance, is highly challenging. Macrovoids and irregular shapes, often observed in hollow fibers, can deteriorate the mechanical properties of HFMs, when operated under high pressure/vibration for prolonged time. A typical HFS system is shown in Figure 3. Indeed, the changes in polymer molecular structure to improve separation performance are limited by the well-known trade-off between permeability and selectivity, i.e., an increase in permeability usually is accompanied by a decrease in selectivity and vice-versa.

The HFS process comprises a large number of process parameters throughout the entire chain of dope formulation, coagulation chemistry, spinneret design and spinning conditions such as air gap, temperature and take-up speed. During spinning, the membrane is formed through phase inversion when the nascent fiber contacts with the coagulant.

Since the polymer dope is extruded simultaneously with the bore fluid in the lumen side of the nascent fiber, coagulation occurs right away at its internal surface after the nascent fiber is emerged from the spinneret. Meanwhile, partial coagulation starts at the outer surface when the nascent fiber goes through the air gap region by the presence of humid air. The whole phase inversion process is completed once the fiber is fully precipitated in the external coagulation bath. The thickness and morphology of the selective layer can be manipulated by varying compositions of the spinning dope, bore fluid and external coagulant as well as take up speed. The complexity of HFS increases as the spinning method advances from single-layer to dual-layer co-extrusion. The dual-layer hollow fibers possess the advantages of cost reduction as well as freedom in customization of materials and morphology for the selective and supporting layers [301,302]. Liu et al. studied the effects of surface modifications on preparation and PV dehydration performance of CS-PS composite hollow-fiber membranes [303]. The fabrication of novel thin-film composite tri-bore hollow fiber (TFC TbHF) membranes for PV dehydration of IPA has been reported [304].

### 4.5. Fabrication via Interfacial Polymerization (IP) Technique

IP is one of the most recognized methods for synthesizing thin composite membranes, mostly used in RO [305] and NF [306,307]. In fact, IP technique was extensively used for fabricating RO membranes since 1960 [308,309,310]. However, the use of this method for synthesizing PV membranes is limited only up to the fabrication of hydrophilic membranes, suitable for dehydration [311,312,313,314,315]. In this context, Parthasarathy et.al. [311] fabricated thin film composite (TFC) HFMs via redox-/photo-IP method for the possible application in simple PV experiment to measure the performance potential of such TFC membranes. Again, Shawky et al. [316] used m-phenylenediamine and trimesoyl chloride, dissolved in water and hexane, respectively, for synthesizing another TFC membrane by IP. This thin film polymer film was mounted on a porous support layer through an in situ polycondensation process [309], followed by the formation of thin film nanocomposite (TFN) membrane, via proper dispersion of inorganic fillers, in the thin polymeric layers, during IP. In fact, several works have been devoted to the incorporation of CNTs [306,317], zeolite [318], silica [319] and silver [320] NPs, into the PAm matrices of the TFNs, for fabricating PV nanocomposites membranes. In fact, the use of IP is highly solicited as ultra-thin membranes can be fabricated by this technique with a rapid rate of polymerization at ambient conditions [321]. This polymerization technique involves rapid reaction between two monomers, dissolved in two immiscible solvents, to yield a continuous polymeric layer at the interface of such two immiscible liquid layers [322]. The selective layer formed on the top of the substrate, during IP, is very thin and thus, possess relatively higher membrane flux. Furthermore, proper selection of suitable monomers for IP can generate thin selective layers having improved chemical resistance, thermal stability and long-term durability [323].

IP has emerged as an important synthetic method for synthesizing PA [324,325,326] and polyimide (PI) [313,314] based TFC membranes, through polycondensation reaction between two monomers on the surface of a microporous support (Table 2). During synthesis of PA membranes, through IP, the amine- and acid chloride-based monomers, dissolved in the organic and aqueous solutions, respectively, are taken and the thin film membrane is obtained from the interface of the two immiscible solvents. The separation potential of such TFC membranes are found to depend largely on the type of monomers used [325,326]. On the other hand, the IP/thermal imidization method has become a very effective method for fabrication of PI based NCMs, with an ultra-thin skin layer, suitable for the dehydration of aqueous organics. In this context, Liu et al. [313] synthesized a TFC, based on PTFE/PA, via IP, using surface-modified PTFE films as substrates. Such a stable composite membrane exhibited high performance potential, i.e., high permeation flux of 1720 g m^−2^ h^−1^ and reasonable SF of 177, for the pervaporative dehydration of IPA-water mixture of 70 wt % of IPA in water. Again, Zuo et al. [314] synthesized HFMs, consisting of a TFC PA layer and a porous Torlons PA-imide substrate through IP, also reported to exhibit high pervaporative performance. In this context, PS and PES are frequently used as the porous substrates for IP [324,325,326]. Kim et al. used PS-polyimide based TFC membranes, prepared by IP of polyimide on a PS support, for pervaporative dehydration of ethanol aqueous solutions [312].

### 4.6. Modifications of Composite Membranes via Physical and Chemical Treatment

The biggest challenge in fabricating the NCMs is to increase the compatibility between the NMs and the polymer matrix that eventually helps the NMs to distribute uniformly into the polymer matrix. Physicochemical modification is one of the best methods that not only restricts the agglomeration tendency of the NMs, but also enhances its dispersibility into the polymer phase. Among different inorganic NFs, silica incorporated composite membranes have generally been found to exhibit significantly high organophilic properties. In fact, silica finds extensive applications owing to the prevalent better thermomechanical and hydrophobic properties. Again, polydimethylsiloxane (PDMS) based membranes have been investigated by several workers for separation of organic-water mixtures. In this regard, for the betterment of compatibility between silica and PDMS, silica surface was hydrophobized with trimethylsilanol. Shirazi et al. showed that the trimethylsilanol hydrophobized silica (TMS-H-Silica) interacts better with the PDMS matrix, resulting in higher rigidity and better organoselectivity of PDMS membrane. Hence, the permeation flux of PDMS-silica NCMs has been found to be moderate, while the respective selectivity is significantly higher than those of the other composite PDMS membranes, since flux and selectivity follow the opposite trend [332]. Again, fumed nano-silica, produced by the flame hydrolysis of chlorosilanes, is commonly used for the reinforcement of polymeric materials/elastomers, such as silicone rubber. Fumed nano-silica always exists in the form of aggregates whose particle size is smaller than those of the conventional silica particles, and the aggregates tend to agglomerate. In fact, the introduction of silica NPs into CS membrane, having a thickness of 28 μm, created extra voids within the polymeric network, resulting in the enhancement of selective permeation rate through the membrane during pervaporative dehydration of the ethanol-water binary mixture [153]. Again, crosslinking on CS reduces the extent of swelling in water and, thus, selectivity is enhanced for the dehydration of ethanol-water mixtures. In succession, a new type of filled NCM was prepared by Liu et al. [153], fabricated through the incorporation of hydrophobic silica into the CS skin layer (Table 3). Moreover, the addition of silica NPs to CS provided extra free volumes in polymer networks for enhanced water permeation. In fact, the membrane, possessing five parts per hundreds of functionalized silica and CS, exhibited SF and permeation flux of 919 and of 410 g m^−2^ h^−1^, respectively, during the dehydration of 90 wt % aqueous ethanol at 70 °C. Furthermore, the surface modification of silica with organosilane reduces the number of superficial silanol groups and enhances organophilicity via attachment of organic moieties. A couple of reactions generally take place: (i) hydrolysis of the silanol groups of the silica and the triethoxysilyl groups of the silane via loss of ethanol and (ii) condensation and crosslinking of the silane molecules. The modified silica NPs have exhibited stronger affinity and enhanced compatibility with poly(phenylene oxide) (PPO), in comparison with the unmodified silica NPs. This generates more restricted permeation through the PPO dense membrane matrix, reduces the diffusion of VOCs, like methanol and MTBE and, consequently, restricts the permeation flux in PV. It has been observed that the permeation flux of the filled PPO membranes, with silane modified silica, is lower than that of the silica filled PPO membranes, especially at high MeOH concentration in feed. As the overall solubilities of the filled PPO membranes are similar, the low permeation flux, observed for the filled PPO membranes with silane modified silica, has been attributed to the betterment of silane modified silica NPs to be dispersed in PPO matrix, introducing more tortuous passage through such membranes than the unmodified silica filled PPO membranes. Consequently, the diffusion of both MeOH and MTBE in the filled PPO membranes, with silane modified silica, has been found to be smaller than that in the unfilled PPO or silica filled PPO membranes. The effect of *γ*-aminopropyltriethoxysilane (APTEOS) content on the permeation flux and SF, in PV of 85 wt % ethanol solution, through the hybrid membranes was studied by Zhang et al. [333]. The SF was observed to increase sharply for such hybrid membranes, containing up to 5 wt % of APTEOS, but followed an opposite trend to decrease on further increase in APTEOS. In fact, SF at 60 °C, was relatively higher when APTEOS content in the hybrid membrane was less than 4.5 wt %. Indeed, SF was found to decrease with an increasing temperature when the amount of APTEOS was more than 4.5 wt % due to the attainment of better mobility of PVA chains with increasing feed temperature, favoring ethanol diffusion and poor water selectivity. Again, PVA-1,2-bis(triethoxysilyl)ethane (PVA-BTEE), an organic-inorganic hybrid membrane, prepared via sol-gel method by Zhang et al. [296], showed good physicochemical properties and permeation performances during pervaporative dehydration of ethanol. The hybrid membrane containing 3 wt % BTEE imparted the highest permeation flux of 0.244 kg m^−2^ h^−1^, while that containing 6 wt % BTEE resulted in the highest SF, which also confirmed the reverse variation of permeation flux and SF. Silicalite-1, a hydrophobic inorganic material, has rarely been used as a filler to fabricate MMMs. Silicalite-1 has a high Si-to-Al ratio that makes it hydrophobic. It has an asymmetrical aperture with a 3D channel system. In this context, Adoor et al. [123] fabricated silicalite-1 incorporated MMMs, of SA and PVA, for pervaporative dehydration of IPA. Indeed, IPA is miscible with water in all proportions and forms azeotrope at 12.5 wt % of water that is difficult to separate by simple distillation owing to the potential health hazards and expensive. It was observed that 5 to 10 wt % silicalite-1 loaded SA and PVA based MMMs showed reasonable dehydration of IPA. Addition of hydrophobic silicalite-1 particles improved the separation potential of the MMMs over those of pristine SA and PVA membranes with a reduction in swelling value. Incorporation of a higher amount (>10 wt %) of silicalite-1 into SA and PVA matrices resulted in brittle and unstable PV membranes for such separation. In fact, restricted permeation through the tortuous paths, created due to the presence of silicalite-1 particles, have resulted in greater transport of water molecules than IPA, since IPA has higher molecular dimension than water. In fact, fluxes of MMMs were lower than pristine SA and PVA membranes due to induced hydrophobicity of SA and PVA matrices as a result of silicalite-1 incorporation within the matrix.

Among various inorganic NPs, zeolite has emerged as one of the best options for the betterment of both selectivity and permeability due to the prevalent unique molecular sieving property and selective adsorption [164,334]. Recently, zeolite filled membranes have exhibited reasonable flux and separation performances than the traditionally used polymeric membranes [335,336]. However, the fabrication of zeolite membranes is expensive and usually brittle. In order to combine the advantageous properties of such two materials, fabrication of MMMs were proposed by the incorporation of inorganic adsorbents, like zeolite particles, within the polymer matrix [337]. This endeavor introduced promising results in rubbery zeolite systems for the pervaporative removal of organic solvents, such as ethanol and methanol, due to the prevalence of better physicochemical properties, as required in PV membranes [338]. Again, chemical reactions generating water, as a product, can be assisted by selective permeation through membranes. The continuous removal of water from the reacting system helps increase in the conversion and a reduction in reaction time. Zeolite-filled membranes perform better in the PV-aided reactions because of facilitated water transport of the membranes as found by Gao et al. [272]. Also, Qiao et al. [274] have fabricated zeolite 5A and 13X filled P84 membranes and investigated the effects of annealing temperature during fabrication, the interaction between zeolite and polymer, zeolite 13X loading on the membrane performance potential and PV separation performance for dehydration of IPA. A higher annealing temperature of 250 °C helps improve the interaction between zeolite and the polymer network through an increase in the flexibility of the polymeric chains and enhances the charge transfer complexes (CTCs) formation. Both permeability and selectivity of P84 membranes have significantly been improved after incorporation of zeolite 5A and 13X NPs. Additionally, zeolite 13X shows better compatibility with P84 polymer than zeolite 5A. In recent years, MMMs, incorporated with zeolites or other inorganic fillers, have widely been studied for pervaporative dehydration of organics.

Another promising NF, used in the membrane matrix, is ZIF material, a subfamily of MOFs, composed of tetrahedral metal ions, such as Zn(II)/Co(II), and a variety of imidazolate linkers to form spatial frameworks. Phan et al. [351] developed the MOFs that possesses tunable structures and favorable affinity with the polymer matrix, as compared to most of the other traditional inorganic particles comprising of rigid frameworks. This promising NF has been incorporated into several polymer matrices, such as polymethylphenylsiloxane, PDMS, CS, PVA, polybenzimidazole (PBI), polyether-block amide, PS, polyimide and polymerizable room temperature IL, for substantial enhancement of physicochemical properties as required for increasing the membrane removal efficiency, and hence, have drawn considerable attention owing to the controllable pore sizes, as well as high porosities. The pore sizes of zeolites are necessarily smaller than the kinetic diameters of most the solvents. In fact, such pore sizes of ZIF particles are found to vary within 0.7–13.1 Å and the corresponding surface area is approximately two times greater than those of inorganic zeolites. Phan and his coworkers [351] successfully synthesized various types of ZIF particles for H_2_ storage and CO_2_ adsorption applications. In another study, Amirilargani and Sadatnia [275] prepared PVA-ZIF-8 MMMs for pervaporative dehydration of IPA, in which the as-prepared ZIF-8 NPs of the maximum 10 wt %, with sizes smaller than 60 nm, were dispersed directly in the matrix of PVA. PDMS-CA NCMs were successfully fabricated by adding modified fumed silica, as filler, by Peng et al. [340], in which the silane coupling agent modified the surface property of the silica particles to enhance the compatibility between the particles and PDMS matrix.

The incorporation of ILs into the membrane system could potentially offer more flexibility in modification of the membranes. The tunable physical and chemical properties of the ILs, depending on the nature of the cation and anion present in their structure, make them appropriate as ‘designer solvents’ [352]. The thin IL membranes based on inorganic supports with controlled pore sizes has been synthesized [353]. It was found that the activation energy of the membranes for permeation was increased with smaller pore sizes, which signified the presence of specific interactions between the IL and the membrane pore surface in controlling the IL properties. A new class of ILs containing magnetic metals, known as Magnetic ILs (MILs), on PVDF support has been studied for CO_2_ separation/concentration that exhibited different behavior in the presence of an external magnetic field [352]. Since the permeants may interact differently under a different magnetic field, these MILs become an attractive alternative in the separation processes [354].

Since the introduction of several nanocarbons (NCs), like CNTs, graphene and various other carbon nanostructures, researchers have been motivated to develop various physico-chemically modified advanced materials. In general, nanostructural carbons tend to agglomerate in nanocomposites, resulting in the deterioration of the properties of individual components. Thus, several methods have been employed for imparting the better dispersion of such materials in a polymer matrix, including the chemical modification of NCs. Chemical modification results in relatively enhanced dispersion in solvents and, thus, betterment of physicochemical and thermomechanical properties of nanocomposites [355]. The relative variation in the properties of nanotubes, the minimum energy conformation of a graphite layer rolled into a cylinder of finite size, depend on the arrangement of the graphene sheets, diameter and length of the tubes and functionalization of nanostructure. The MWCNTs include a coaxial assembly of several single-walled CNTs (SWCNTs) (“Russian doll” structure), separated by a distance of ~0.34 nm, which is slightly higher than the interlayer distance in single-crystal graphite [356], whereas the double-walled CNTs (DWCNTs) consist of merely the two graphene layers. The most exciting feature of DWCNTs is that the outer wall solely involves, in contrast to SWCNTs, functionalization, whereas the inner tube remains intact resulting in the retention of mechanical properties.

**Figure 4:**
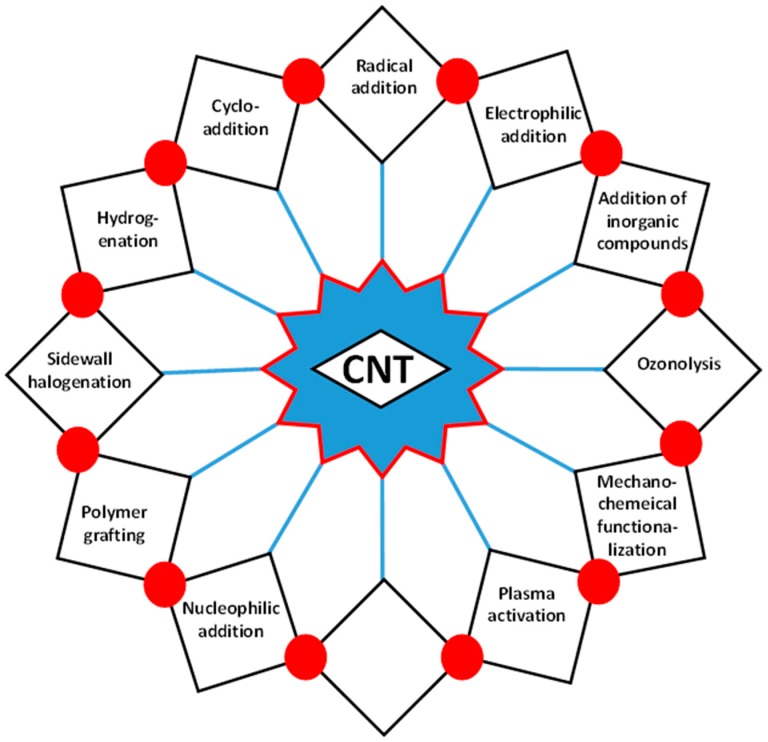
Various types of functionalization of carbon nanotubes.

Several types of chemical modifications have been introduced for the covalent functionalization on CNTs by disruption of the conjugation (Figure 4). For example, hydrogenation by Birch reduction results in the modification of unsaturated backbone of the CNTs [357] as reflected by the corrugation and disorderly alignment of CNT walls. Few current investigations have also been investigated by several workers that help us to understand the dominant role of the functionalized SWCNTs as reinforcing materials in various polymeric systems. After the attainment of reasonable functionalization on the surface of MWCNT by various chemically reactive groups, grafting of polymer chains on the functionalized nanotube surface is allowed to proceed. The polymer-grafted nanotubes impart substantially improved dispersion in organic solvents as compared to pristine MWCNT [358]. The nanocomposites exhibit improved thermomechanical stability as a result of chemical reaction between the functional groups of filler and the polymer matrix. A significant number of research has been conducted using modified CNTs for PV [170,171,172,173,259,359] and those NCMs have exhibited significant enhancement in separation performances.

Polyphosphazene nanotube (PZSNT) is a novel type of nanotube, which can be prepared by the reaction between hexachlorocyclotriphosphazene (HCCP) and 4,4′-sulfonyldiphenol (BPS). As PZSNT content increased from 0 to 10 wt %, both permeation flux and SF were found to increase. However, the increase in PZSNT diameter leads to the increase in permeation flux and SF.

In addition to such techniques, direct grafting of different nanoparticles into the polymer matrix is also highly attractive towards fabrication of composite membranes. In fact, Mauter et al. [360] developed silver nanoparticles (AgNPs) encapsulated positively charged polyethyleneimine (PEI) and plasma modified polysulfone based ultrafiltration membrane with and without 1-ethyl-3-(3-dimethylaminopropyl) carbodiimide hydrochloride. Also, Liang et al. [361] fabricated silica nanoparticles grafted polyvinylidene fluoride (PVDF) membrane, suitable for ultrafiltration.

## 5. Specific Applications of PV Membranes

Besides straightforward dehydration, separation and purification of organics, NCMs based PV has recently been employed in many other areas, including desalination, desulphurization, chemical reactions, etc. These membranes exhibit substantial improvement in terms of selectivity, permeability and long-term stability and shows high potential for real time applications.

### 5.1. Membrane Reactor

A membrane reactor is a device that combines shifting of a chemical equilibrium towards the forward direction with simultaneous separation through the involvement of a membrane during a reaction. In such a case, elimination of at least one end product is essential to drive the equilibrium into the product side for spontaneous enhancement of the final product.

#### 5.1.1. Roles of Membrane in Membrane Reactor

The role of a membrane in membrane reactors can be of the following three types: extractor (to remove the product(s) for enhancing the rate of the reaction via shifting the equilibrium in forward direction), distributor (to restrict the unwanted side reactions by the precise addition of reactant(s)) and active contactor (to control the diffusion rate of reactants towards the catalyst chamber for betterment of the reaction). In the first two cases, membranes are devoid of catalytic properties, whereas in the active contactor mode, the membrane also plays the role of a catalyst through the prevalent diffusion barrier ability. Thus, the use of active contactor mode is extensive and can further be classified into two categories: forced flow contactor- and opposing reactant-mode, depending upon the activity of the membrane. In fact, the forced flow contactor mode is mainly applied for the total oxidation of VOCs. The distributor mode, on the other hand, finds immense application for limiting consecutive and parallel deep oxidation reactions for partial oxidation, oxidative dehydrogenation of hydrocarbons and oxidative coupling of methane. In fact, the extra cost, introduced due to the involvement of a membrane into the reactor, can be counterbalanced by the attainment of several advantages, like smoothness of operation, low-maintenance instrumentation, energy/cost-effectivity, use of the minimum amount of chemicals and high cost to performance ratio of the membrane [362]. The membrane should be highly component selective, i.e., only the component to be separated will be able to permeate through the membrane [363]. In this regards, preferential permeation of lyophilic and lyophobic components is assisted by the relative variations of lyophilic and lyophobic functional groups and physicochemical properties of the membranes [364,365,366]. The design for introducing suitable physicochemical properties of the membrane is, however, controlled in such way that the membrane can retain the compactness/network even in contact with solvent molecules. The membrane should possess enough thermomechanical-/chemical-stability to work under the working temperature and pressure of unit operation [367,368]. Indeed, a good balance of mechanical properties, like tensile strength (TS) and elongation at break (EAB), is highly solicited for producing a stable PV membrane to work under harsh reaction conditions [369]. In this context, it is reported that both TS and EAB are severely affected by the methods and several input variables, including different relative mole ratios of all ingredients and the respective nature, vulcanization-/crosslinking-/curing-agent, time and temperature, of membrane synthesis [70]. Indeed, the exact balance between TS and EAB can be obtained by implementing response surface methodology (RSM), a frequently used statistical technique, via performing minimum number of experiments/runs [370]. In fact, the use of RSM to obtain the amounts of ingredients, like the crosslinker, filler and accelerator, for synthesizing accelerated sulfur vulcanized and carbon nano particle filled natural rubber membrane has been reported by Karmakar et al. [371], in which a substantial variation of both TS and EAB has been observed with the little change in such amounts. Also, there should be an optimum balance between the flux and SF of the membrane.

Though both inorganic and organic membranes can be used in membrane reactors, yet the use of inorganic membranes has been found to be more advantageous, owing to the superior thermal properties and pH resistance ability. Inorganic membranes can withstand high thermal stress and thus, restrict the structural deformation at much higher temperature than the organic membranes. Additionally, inorganic membranes are capable of enduring organic solvents, chlorine and other corrosive chemicals, better than organic membranes, allowing the use of more effective, and yet, corrosive chemicals and hazardous cleaning procedures.

**Figure 5:**
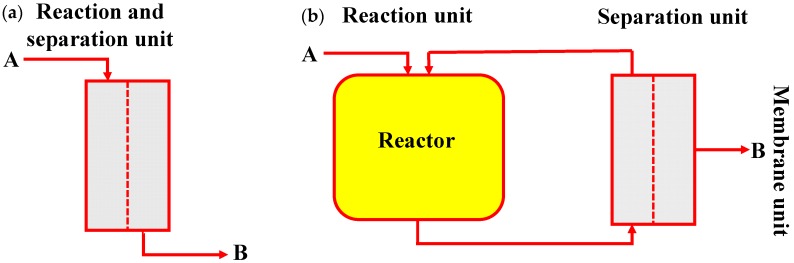
Membrane reactors: (**a**) reaction and separation are coupled in one unit (catalytic membrane reactor) and (**b**) reaction unit and membrane unit are separated (membrane recycle reactor).

Polymeric membranes, on the other hand, can only survive in moderate conditions. Thus, membrane reactors, possessing polymeric membranes, can be operated for enzyme-catalyzed bio-reactions and low temperature reactions, including saccharification of celluloses and hydrolysis of proteins. However, several catalytic processes, used in different industries, include high temperature and harsh chemical conditions. These situations undoubtedly point out the rational applicability of inorganic membranes, like commercially available glass, ceramic and metal membranes, in the field of membrane reactor or membrane catalysis. Some auspicious applications of inorganic membranes in membrane reactor include dehydrogenation, hydrogenation and oxidation reactions, like oxidative coupling of methane, synthesis of butadiene, styrene and ethene from butane, ethyl benzene and ethane, respectively, and water-gas shift (WGS) reaction. In such membrane reactors, two basic mechanisms can be depicted: (i) reaction and separation are combined in one unit (Figure 5a) and (ii) reaction and separation are not combined and the reactants are recycled along a membrane system (Figure 5b). The use of former instrumentation is limited for the combination of inorganic, i.e., ceramics and metals, and polymeric membranes along with the catalyst. However, the second arrangement is applicable to any membranes.

The presence of selective component in the membrane could also enhance the overall performances. Absorption properties and mass transfer of coal combustion flue gases in IL (1-ethyl-3-methylimidazolium ethylsulfate) using a polypropylene HFM contactor [372] showed that the use ILs with lower vapor pressure as absorption liquids may contribute significantly to the performance of a zero solvent emission process [372,373]. The membrane MTC (k_m_) was calculated using a microscopic model based on laminar flow and was found as high as 3.78 × 10^−6^ m s^−1^, which is about five times higher than that obtained in the macroscopic model [374]. Norkobilov et al. studied the manufacturing process of ethyl tert-butyl ether (ETBE), which involves the separation of ETBE from its mixtures with C4 hydrocarbons and unreacted ethanol. During this process, the unreacted ethanol and ETBE forms azeotropic mixtures, which are difficult to separate by conventional distillation. A process simulation software Aspen Plus was used to carry out the simulation tasks and the process flowsheet analysis has been compared [375].

#### 5.1.2. Use of Catalyst in Membrane Reactors

**Figure 6:**
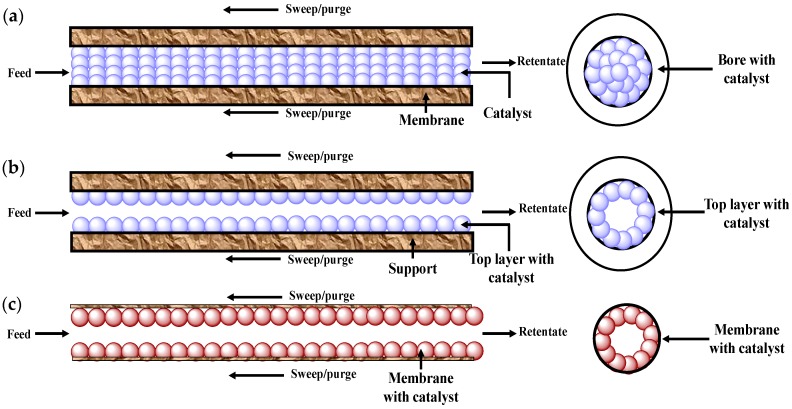
Membrane reactors in a tubular configuration (**a**) bore of the tube fitted with catalyst, (**b**) top layer filled with catalyst and (**c**) membrane wall filled with catalyst.

Most of the chemical reactions follow a multi-step reaction mechanism containing one or more step(s) as equilibrium. Catalysts are therefore employed to speed up the overall rate of the reaction. In fact, catalyst must be combined with the membrane system through different arrangements, as shown in Figure 6. Of the three different arrangements, the first one is the simplest, where the catalyst is buried inside the bore of the tube (Figure 6a). This instrumentation is particularly useful for the reactions, where changing in catalyst, during the course of reaction, is essential for catalyst poisoning. The advantages of this system are the simplicity in preparation/operation and reloading the new catalyst in case of catalyst poisoning. In other two arrangements, catalyst is immobilized onto the membrane, either in the top layer (Figure 6b) or in the membrane wall (Figure 6c). However, instead of the prevalent huge advantages and application prospects, the commercialization of membrane reactors in large-scale industries are emerging slowly due to some practical limitations, like low SF, leakage at higher temperature, poisoning of catalyst and mass transfer limitations. Another major drawback of membrane based separation is the inverse variations of selectivity and permeability. In this context, the use of NCMs, possessing both enriched selectivity and permeability, can successfully be employed to solve such problems.

#### 5.1.3. Membrane Chemical Reactors

##### Oxidative Coupling of Methane (OCM)

With depletion in liquid petroleum, natural gas, containing primarily methane (>95 wt %), is reasonably expected to be one of the main resources for the production of chemicals and liquid fuels. The two most frequently used methods for converting methane into different chemicals and liquid fuels may proceed through direct and indirect routes. In the direct route, OCM occurs to produce different C2 products, like ethane and ethylene, whereas in the indirect route, production of syngas, i.e., H_2_ and CO mixture, is produced via steam reformation or partial oxidation of CH_4_ followed by further conversion into higher hydrocarbons by the Fischer-Tropsch reaction. Two types of membrane reactors have been under the utmost consideration for OCM since the recent past: (i) porous ceramic membrane reactors and (ii) dense ionic or mixed-conducting oxide membrane reactors. Porous membranes, such as alumina, zirconia and vycor glass, possess substantial stability during chemical reactions, yet exhibit poor O_2_ selectivity. Hollow-fiber ceramic membranes, possessing an asymmetric structure, i.e., a thin dense and separating layer integrated with a porous substrate of the same material, have recently been under consideration. As compared to the disk-shaped membranes, these hollow-fiber membranes possess considerably larger membrane area per unit volume for O_2_ permeation. Two different approaches have frequently been studied for improving the selectivity and yield of OCM. In the first methodology, the membrane is used as the O_2_ separator, containing air as the reactant, and distributor [376,377], whereas in the other approach, the surface of the O_2_ semipermeable membrane is made catalytically active and selective towards OCM, such that O_2_ can permeate through the membrane and react, in the form of atomic oxygen, with the membrane on the other surface. Few studies involving both perovskite and fluorite type membranes have been devoted based on the second approach for OCM. Except the works reported by Nozaki and Fujimoto [378] and Guo et al. [379], the membrane surface was kept unmodified, in all other reported works, with other catalysts. Among all the reported perovskite type ceramic membrane reactors, the highest C2 yield of 16.5 wt % was obtained by Lu et al. [380] by using a tubular BaGe_0.8_Gd_0.2_O_3_ membrane.

To date, the development of suitable polymer membrane reactor for OCM application is still a difficult task because of the high operating temperature, membrane fouling and the low mechanical strength. There are very few polymer-made membranes are commercially available that can sustain the harsh reaction conditions. Polymer membranes have been reported for ethane-ethylene separation consisting of silver polymer electrolyte membranes and silicate-1 membrane [381,382]. Polymeric silica gel deposited on γ-Al_2_O_3_ support has been synthesized to achieve the pore size ~10 Å [383]. The hybrid membrane exhibited a very high SF of 160 when applied for the separation of C_3_H_8_-H_2_ mixture. However, the permeability and thermal stability was found very poor. Several membranes including Matrimid^®^ polyimide flat-sheet membrane have been used for the separation of CO_2_ from OCM process application [384,385,386,387,388,389]. However, as this membrane showed inferior selectivity, a considerable amount of hydrocarbons usually escape from the stream leaving the OCM reactor. Usually, these membranes are utilized primarily to separate large portion of the CO_2_ content (~40%). Despite several disadvantages, PV membrane reactors are becoming highly attractive owing to the simplicity of overall process, ease of separation and low energy requirement [390].

##### Water Gas Shift (WGS) Reaction

Since the past few decades, use of H_2_ as a carrier in the clean energy process has been under prime consideration. Meanwhile, some new technologies have promoted improvements in the H_2_ production cycle. As light hydrocarbons provide better and simple ways to produce H_2_, many studies based on the use of catalytic membrane reactors in integrated plants for H_2_ production with low CO content have been reported [391]. Currently, H_2_ produced by reforming and/or partial oxidation of light hydrocarbons, like natural gas, is found to contain CO, CO_2_, H_2_O and traces of CH_4_. Thus, in an integrated membrane plant for H_2_ production, it is important to upgrade the streams coming from the reformer, via the WGS reaction, to reduce the CO content and simultaneous generation of more H_2_. As water gas reaction is exothermic [391,392], use of a membrane reactor for the WGS reaction allows higher conversion at relatively higher temperature. As a consequence, the catalyst amount, required for a given conversion, can significantly be reduced. For instance, the catalyst volume for the traditional reactor equilibrium to reach the 99% conversion at 280 °C and 200 kPa reduces to 50% of a traditional reactor at the same operating conditions [393]. H_2_-separation membranes have been developed using various inorganic materials, such as palladium and its alloys, silica and alumina. One of the attractive alternatives for the high permselective membrane is a silica layer formed on mesoporous γ-alumina film, supported on a porous stainless steel. Brunetti and coworkers [394] investigated WGS reaction in a membrane reactor using a porous stainless steel supported silica membrane and a CuO/CeO_2_ based commercial catalyst in a temperature range of 220–290 °C up to 600 kPa. An excellent CO conversion of 95% was noted at 280 °C and 400 kPa.

The production of H_2_ by employing the direct water splitting requires an efficient H_2_ and O_2_ separation technique from its water vapor mixture. The availability and viability of polymeric membrane materials are limited due to the limited thermal stability of the organic polymers [395]. The development of polymer electrolysis membrane (PEM) cells, using acidic ionomer, could potentially supply the world’s hydrogen demand [396,397]. The advanced polymeric materials exhibit a low-cost alternative to Pd at the cost of inferior gas permselectivities [398,399]. Investigations have been carried out that mainly focus on the enhancement in permeabilities and permselectivities through alteration of the polymer membrane structures. Jamshidi et al. synthesized a Pd membrane reactor by the electroless plating technique (ELP) “organic-inorganic” method for H_2_ production via WGS reaction, where the Pd NPs were embedded with the polyethylene glycol (PEG) [400]. Depending on operating conditions, the membrane exhibited an infinite selectivity for H_2_/N_2_ and a H_2_ flux of 0.004–0.016 mol m^−2^ s^−1^. The membrane reactor performed 98.5% CO conversion with 16% H_2_ recovery at 450 °C. The membrane was found to be quite stable during WGS catalytic membrane reactors (CMR) tests.

##### Membrane Reactors in the Petrochemical Industries

**Figure 7:**
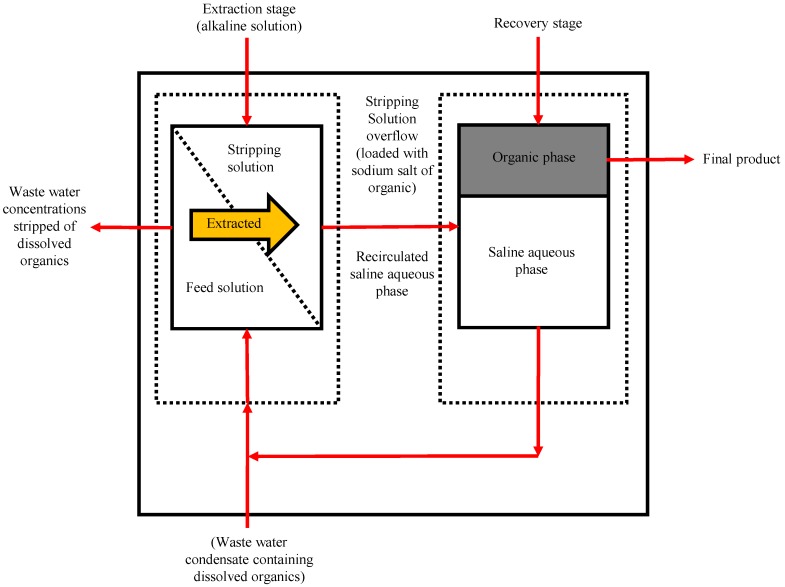
Operating principle of membrane aromatic recovery system (MARS) for recovery of phenolic compounds.

In petrochemical industries, olefins, like ethylene and propylene, are the most frequently used raw ingredients for producing different polyolefins, such as polyethylene, polypropylene, styrene, ethyl benzene, ethylene dichloride, AN and isopropanol. However, one of the most important steps of such manufacture is the large-scale continuous separation of olefin from the corresponding paraffin. In addition, dehydrogenation, oxidative coupling and steam reformation of methane and water gas shift reaction are also important in petrochemical industries. The membrane based separation technique has been established as the most effective, low-cost and green technique. Petrochemical waste streams may contain phenolic compounds or aromatic amines. They are highly toxic and inhibitory to biological treatment at high concentrations. Membrane aromatic recovery system (MARS) is a relatively new process for recovery of aromatic acids and bases (Figure 7). Wastewater in petrochemical industry is currently treated by activated sludge process with pretreatment of oil-water separation. Tightening effluent regulations and an increasing need for reuse of the treated water have generated added interest for the treatment of petrochemical wastewater with advanced membrane bio-reactor (MBR) process.

##### Esterification Reaction

Recent increasing demands for organic esters in biotechnology and in chemical industry, esterification reaction has received significant attention. The enzyme-catalyzed esterification, especially the lipase, is of great importance. The energy demand, cost and lower selectivity limit the reaction success in commercial scale production. In a conventional esterification reaction, presence of excess water plays a negative role on overall production rate and enzyme activity. Constant removal of water at low temperature by PV could help to achieve higher productivity [401]. Inoue et al. employed different types of zeolite membranes to study the water removal from various organic aqueous solutions, such as equimolar mixture of water, ethanol, acetic acid and ethyl acetate, by PV process [402]. These membranes were observed to be highly hydrophilic and resistant to acid solution. It was observed that higher selectivity could be attainable at milder reaction condition [403]. The stoichiometric ester condensation reactions have been performed at lower temperature (<50 °C) by these membranes. The ester yields were found to be more than 20% of that of the equilibrium values [402].

Several researches have been carried out with NCMs for the dehydration of esters. The incorporation of NMs effectively removes the water from the system, generated during the reaction that eventually, helps enhanced conversion of the reactants into the desired product [131]. Torabi et al. synthesized cross-linked PVA-silica NCMs for the dehydration of the reaction mixture of methyl acetate through the coupled PV-reaction [404]. The optimum reaction conversion was observed to be 94%, which was achieved with 20 wt % silica in the NCMs over a period of 240 min. In another study, an enhanced performance in the esterification process of acetic acid with methanol was observed with the incorporation of treated MWCNT into the cross-linked PVA membrane, using citric acid as the cross-linking agent [361]. The final amount of product was increased from 52.30% to 99.25% using the membrane with 2 wt % TCN in comparison with pure membrane at optimized condition and 4 h of operation. The NCM fabricated with poly(2,6-dimethyl-1,4-phenylene oxide) (PPO) and fullerene C_60_ (3 wt %) as a selective layer of composite membranes has been studied in PV coupling esterification reaction [405].

### 5.2. Pervaporative Desalination

Shortage of water resources is one of the biggest problems in the world. In fact, more than one-third of the world’s total population suffer from the scarcity of safe drinking water [406]. Again, pollution and exploitation of groundwater decreases the quantity of surface water and/or quality of the available natural water resources in many regions. In addition, higher living standards, especially in first-world countries, results in higher per capita water consumption enhancing the water scarcity. Desalination of saltwater, including seawater and brackish water, possessing salinity values of ~3.5 and 0.05–3%, respectively, is an important technology for solving such water crisis [407]. RO, the most commonly used desalination technique for producing fresh water, also faces some severe disadvantages, including high operational pressure, high energy cost and easy fouling. Moreover, the potential of RO system for desalination is only limited up to 50%. Again, the concentrated sea water, a by-product of RO, may also induce secondary pollution towards the eco-system. In order to cope with such limitations, PV, an efficient membrane separation technology, has been adopted for saltwater desalination. In fact, pervaporative desalination of the seawater is considered to be the potential alternative methods for solving the water scarcity owing to several advantages, like high energy conservation at the expense of low cost, high efficiency (~100% of salt rejection) [406] and better handling ability of water with high salinity [408,409,410,411]. Compared with the membrane distillation, pervaporative desalination using hydrophilic materials can effectively reduce membrane fouling and maintain membrane separation performance.

PV is equipped with non-porous, dense polymeric membranes and the separation mechanism is based on the differential rate of diffusion of constituents through these membranes. PV of an aqueous salt solution can be considered as the separation of a pseudo-liquid mixture containing free water molecules and bulkier hydrated ions formed in solution upon dissociation of the salt in water [412]. One of the main limitations for PV desalination is the lack of the high performance membranes with both high permeate flux and good salt rejection [412,413]. In this context, Xie et al. reported the development of a new type of hybrid composite membrane based on PVA-MA-silica for pervaporative desalination [414]. Similarly, a novel thin film nanofiber PV composite (TFNPVC) membrane for pervaporative desalination was first synthesized by Liang et al. [415], via electro spraying a smooth and ultra-thin PVA skin layer. In addition, Cheng et al. fabricated a robust GO thin-film nanofibrous composite membrane with integrated structure, exhibited a remarkably high water flux and salt rejection of 69.1 L m ^−2^ h^−1^ and 99.9%, respectively, from the feed water of 35 g L^−1^ NaCl at 70 °C. The NCM also demonstrated excellent stability over a long testing period of 24 h [416].

In fact, the chemical properties of membrane materials, used in PV, play the key role in designing a high-performance PV set-up. Currently, PV membranes for desalination have been fabricated by different polymeric materials/elastomers (i.e., crosslinked PVA/PDMS), inorganic materials (i.e., NaA zeolite) and polymer-inorganic hybrid materials (i.e., PVA-maleic acid (MA)-silica) [415]. Although all such membranes have shown significantly high rejection performance, for monovalent ions, the water flux of all these membranes is generally quite low. In this context, GO, an ultra-thin 2D smart material is under supreme consideration due to its multi-functional surface chemistry [417], having a variety of functional groups, like epoxide, carbonyl and hydroxyl. GO has been used as a starting material for preparation of thin films, paper-like materials or membranes suitable for salt rejection [418]. Many efforts have been made to fabricate highly permeable GO containing membranes to facilitate the water transport along the nanochannels between graphene sheets [419,420]. Liang et al. [367] reported that the as-prepared GO-PAN composite PV membranes exhibited sufficiently high water flux, up to 65.1 L m^−2^ h^−1^, with the high rejection for desalination (~99.8% at 90 °C). It is noteworthy that the composite membranes retain high performance in treating high salinity water even with a salt concentration up to 100,000 ppm. A new preparation route of silica-PVA membrane is reported by Chaudhari et al. [421], carried out in acidified and hydrated ethanol via a sol-gel reaction to form hybrid membrane structure, which was found to exhibit superior water permeability of 96 L m^−2^ h^−1^ than the other reported membranes in PV desalination of saline water of 2000 ppm NaCl. Feng et al. [422] developed GO-PI MMMs for seawater desalination by the incorporation of GO into PI polymer matrix through a phase inversion method. Due to the combination of both advantages of GO and PI, the GO-PI MMMs exhibited water permeation flux as high as 36.1 kg m^−2^ h^−1^ and a high ion rejection ~99.9% to envisage the excellent water permeability and salt rejection for seawater desalination at 90 °C. The desalination performances of GO-PI MMMs kept unchanged for 120 h at 75 °C. PV under various operating conditions was carried out by Xie et al. [423] to evaluate the separation performance of aqueous salt solution through the hybrid PVA-MA-silica membrane. The PV desalination performance of hybrid PVA-MA-silica membrane is observed to be independent of the operating conditions due to the non-volatile nature of NaCl and a high water flux of 11.7 kg m^−2^ h^−1^ was achieved at a feed temperature of 65 °C and a vacuum of 6 torr. The salt rejection remained high, up to 99.9%, under all operating conditions.

### 5.3. Pervaporative Desulfurization

The sulfur derivatives, used in automobile fuel, are the main sources of various sulfur oxides (SO*_x_*), in atmosphere [424,425], which cause severe health damage and serious environmental pollution [426]. In fact, such sulfur poisoning leads to the formation acid rain, which creates adverse effects on all the living organisms as well as the archaeological establishments. Again, the presence of sulfur in gasoline deteriorates the activity of vehicle catalytic converters, via poisoning the active sites of motor vehicles, and enhances the emission of poisoning SO*_x_* [427]. Gasoline desulfurization has therefore become an essential task worldwide, particularly in the developed countries, in order to eliminate or at least reduce the sulfur content in automobile fuels. In fact, development of cost-/performance-efficient desulfurization technologies, for removing sulfur from gasoline, is gaining high insight all over the world. The most important sulfur-containing components, present in fluid catalytic cracking (FCC) gasoline, are thiophenes, mercaptans, sulfides, disulfides and its derivatives.

Of the two main desulfurization techniques, i.e., hydrodesulfurization (HDS) and non-HDS [428], HDS is the most commonly used desulfurization technique at present [429]. However, the HDS is associated with the high cost of H_2_ along with the simultaneous reduction in the octane number of gasoline. Furthermore, the removal of thiophenes from gasoline by the conventional HDS is the most difficult, due to the harsh operating conditions, such as high temperature, pressure and H_2_ atmosphere. Therefore, some alternative technologies, like adsorption, biological method, extraction and PV, have recently been developed for gasoline desulfurization [430].

**Figure 8:**
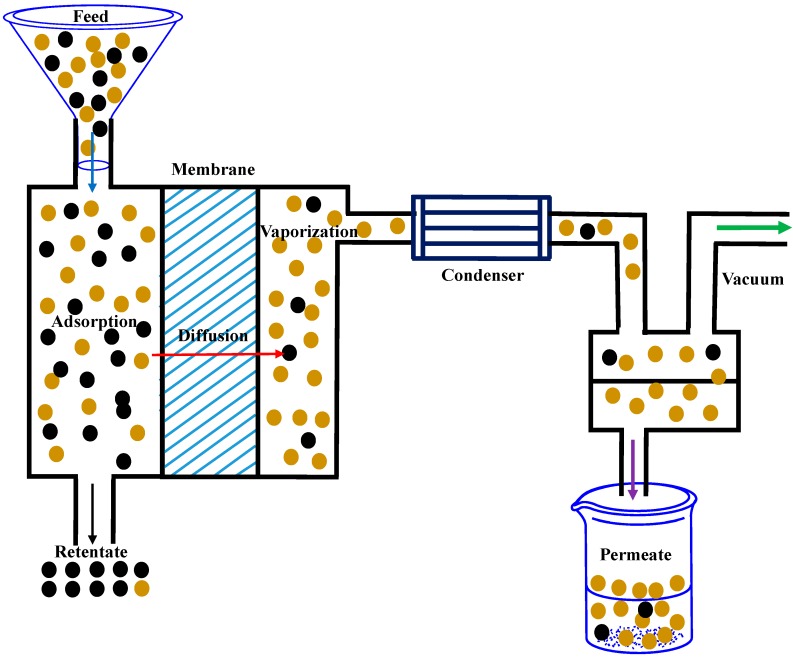
Schematic of basic gasoline desulphurization by PV.

Pervaporative desulfurization involves the extraction of aromatics from aliphatic hydrocarbons, via solvent diffusion transport, through a non-porous membrane. In fact, partial vaporization, during pervaporative desulfurization, occurs due to the application of vacuum, while the membrane acts as a sulfur-selective barrier between the two phases: liquid phase retentate and vapor phase permeate (Figure 8). The non-porous polymeric membrane, serving as the separating barrier, effectively diffuses sulfur containing molecules and some other hydrocarbon molecules, via dissolution, when these components pass through the membrane. As a result, the sulfur containing molecules can be dragged through the membrane and concentrated at the permeate side. Another approach is to use a membrane that enriches the sulfur content in the retentate side by the opposite effect. However, as the PV alone cannot drastically reduce the sulfur content in the mixture to make it below the desired level, a multi-stage process design, comprising of the combination of PV and adsorption, namely predesulfurization and fine desulfurization are generally used.

Pervaporative desulfurization is gaining increasing attention due to its distinct advantages, such as lower capital and operating expenses, higher selectivity as well as easier scale up [430,431]. The S-Brane™ technology, developed by W.R. Grace & Co., Columbia, MD, USA, is a renowned PV-based industrially viable technology, which has utilized for selective removal of sulfur-containing hydrocarbon molecules from FCC and other naphtha streams with a production efficiency of 300 barrels per day. Pervaporative desulfurization, applied alone or coupled with the conventional HDS process, effectively reduces the capital expenditure and operating cost without scarifying the octane number. In this context, Kong et al. applied crosslinked PEG membranes for sulfur removal from FCC gasoline [432]. PDMS membranes were studied and applied for pervaporative desulfurization of gasoline [433].

PDMS possesses an SP of 15.5 kJ^1/2^ cm^−3/2^, closely resembling thiophene and hence, is perfectly suitable for the preferential transport from gasoline. In fact, recently developed PDMS-based membranes have been found to possess significantly high β of ~4.2–4.9 and flux of 1500–5370 g m^−2^ h^−1^ for the desulfurization of thiophene-n-octane gasoline, as reported by Cao et al. [434]. In order to improve the stability and performance of PDMS membranes, various inorganic particles, especially in the nano range, are incorporated into the polymeric matrix. Organic-inorganic nanocomposite materials can preserve chemical reactivity and flexibility of the organo-functional groups, while strengthening the mechanical and thermal stabilities. Li et al. [435] investigated the effect of SiO_2_-NPs, present into the PDMS membrane matrix, for the pervaporative desulfurization of thiophene-n-octane binary mixture as model gasoline. Various membranes were prepared by the incorporation of a varying amount of SiO_2_, and the membrane performance, towards thiophene removal, was observed to be manifoldly enhanced with the increase in SiO_2_ of the composite membrane. The fabricated NCMs exhibited enhanced permeability and permselectivity in the pervaporative desulfurization. It was observed that under the condition of 500 ppm sulfur in feed (40 L h^−1^) at 30 °C, the β of 4.83–5.82 with a normalized permeation rate of 6.61–10.76 × 10^−5^ kg m m^−2^ h^−1^ was obtained. The enrichment of performance potential of PDMS membranes due to the incorporation of NPs encouraged Yang et al. [436] to fabricate PDMS-graphene nanosheets (PDMS-GNS) hybrid membranes for the pervaporative desulfurization of n-octane-thiophene mixture. These hybrid NCMs not only exhibited excellent thermal and mechanical stabilities, but also showed excellent potential for thiophene permeation. In fact, the NCM, comprising of 0.2 wt % mass ratio of GNS-PDMS, was found to exhibit the permeation flux of 6.22 kg m^−2^ h^−1^, which was ~66% higher than the mere PDMS membrane.

## 6. Perceptions and Conclusions

PV captures a special position as an impressive separation technique in chemical industry. Currently, more than hundreds of PV units are operating throughout the world, primarily to purify the chemicals. The development of enhanced PV technology has visualized a clean and invulnerable future that encourages the interest of energy conservation, accompanied by generation of minimum waste and zero emission around the ecosphere. The PV process is a pioneering and fast emerging separation technology that could be employed to a great extent in environmental protection, clean resources, food, chemical and pharmaceutical areas. Among all available technologies, PV is considered as the most energy-efficient and green technology for separation of azeotropic/close boiling mixtures, recovery of traces of impurities from aqueous solutions as well as treating the heat sensitive biomaterials. Although distillation is the most commonly used separation method to separate liquid mixtures, this method is not suitable to obtain high purity solvents from azeotropic mixtures. In fact, membrane based separation technologies have drawn utmost attention due to high selectivity, low energy consumption, moderate cost-to-performance ratio and compact modular design. However, in spite of the prevalent excellent separation potential, the availability of pure inorganic membranes is inadequate for cost-friendly commercial applications. Conversely, organic polymers constitute versatile barrier membranes owing to better film-forming ability, low cost and susceptible towards physicochemical modification. Nevertheless, the amount of material permFeates through such homogeneous dense membranes are usually very low and remains a critical issue obstructing the large scale industrial application of PV. Moreover, the technology is also suffering a ‘trade off’ relationship between flux and selectivity, which restricts its commercial acceptability.

In fact, the primary function of fillers is to act as physical/chemical binders, within the polymeric chains of composite membranes, for optimizing the essential mechanical properties, like TS, EAB, modulus etc. Indeed, incorporation of fillers into the polymer matrix engenders covalent and/or van der Waals force of interactions with the polymer matrix that is the basic cause of reinforcement. However, the variation in particle size of fillers has been found to possess dominant effect of the reinforcement property. In fact, fillers, having particle sizes within 100 nm, i.e., nanoparticles, have been found to possess the strongest reinforcement strength. Therefore, recently, nanotechnology is being considered as one of the most prospective regions for resolving the technical and commercial challenges, coupled with the separation and purification technologies. The development of well-defined nanostructured materials with unique properties renewed the traditional opinion of separation methods, streaming new separation approaches that surpass the existing accomplishments. The utilization of these NMs in membrane separation techniques not only answers the ‘trade off’ issue associated with PV technologies, but also opens the path towards real time applications. The newly fabricated NCMs are found to be superior in terms of permeability, selectivity and long-term stability. These novel NCMs will continue to develop improved cost efficient systems and module design, help to construct an optimized alliance and integration with existent technologies, and to reduce the overall capital investment.

## Figures and Tables

**Table 1:** Fabrication of nanocomposite polymeric membranes by solution blending method.

Organic Polymers	Inorganic NPs/Mesh Size	Amount of Organic Polymer Used (wt %)	Amount of Inorganic Nanocomposite Used (wt %)	Reference
PSf	ZrO_2_/~1.00 μm	18	0–90	[285]
Poly(amic acid)	TEOS/7–20 nm	61–79	39–21	[290]
EVA	12Me-MMT	-	-	[291]
EAA	MMT	5 g/200 mL	2	[292]
PVC	Na-MMT	10 g	0.2 g	[293]
PVC	OMMT	10 g	0.2 g	[293]
PVC	MMT	-	1–25 phr	[294]
PVC	Na-MMT	-	1–25 phr	[294]
PVC	OMMT	-	1–25 phr	[294]
EVA	12Me-MMT	-	0–6	[295]
CA	Alumina	10	50	[286]

**Table 2:** Use of NCMs, synthesized through interfacial polymerization, for the PV.

Organic Polymer Used	NP Used	Thickness (μm)	Utility	TF (g m^−2^ h^−1^)/Temperature (K)/Feed (wt %)	SF/Permeability	Ref.
PTFE ^a^	PA ^b^	–	PV ^c^ (IPA ^d^/H_2_O)	1720 ± 150/343/70:30 (IPA/H_2_O)/–	177 (IPA/H_2_O)/–	[313]
PVA ^e^	APTEOS hybrid ^f^	18	PV (IPA/H_2_O)	26.5/303/90:10 (IPA/H_2_O)/–	1580 (H_2_O/IPA)/–	[327]
PVA	Zeolite	–	PV (IPA/H_2_O)	3.2/303/90:10 (IPA/H_2_O)/–	216 (H_2_O/IPA)/–	[328]
CS ^g^	Zeolite	40	PV (THF ^h^/H_2_O)	170/303/95:5 (THF/H_2_O)/–	2140 (THF/H_2_O)/–	[329]
PVA	1.5 wt % FeO	45	PV (IPA/H_2_O)	95/303/90:10 (IPA/H_2_O)/–	122 (H_2_O/IPA)/–	[330]
PVA	3.0 wt % FeO	45	PV (IPA/H_2_O)	82/303/90:10 (IPA/H_2_O)/–	143 (H_2_O/IPA)/–	[330]
PVA	4.5 wt % FeO	45	PV (IPA/H_2_O)	79/303/90:10 (IPA/H_2_O)/–	470 (H_2_O/IPA)/–	[330]
PVA	1.5 wt % FeO	45	PV (DO ^j^/H2O)	98/303/90:10 (DO/H_2_O)/–	82 (H_2_O/DO)/–	[330]
PVA	3.0 wt % FeO	45	PV (DO/H2O)	91/303/90:10 (DO/H_2_O)/–	104 (H_2_O/DO)/–	[330]
PVA	4.5 wt % FeO	45	PV (DO/H2O)	84/303/90:10 (DO/H_2_O)/–	144 (H_2_O/DO)/–	[330]
PVA	1.5 wt % FeO	45	PV (THF/H_2_O)	180/303/90:10 (THF/H_2_O)/–	342 (H_2_O/THF)/–	[330]
PVA	3.0 wt % FeO	45	PV (THF/H_2_O)	139/303/90:10 (THF/H_2_O)/–	421 (H_2_O/THF)/–	[330]
PVA	4.5 wt % FeO	45	PV (THF/H_2_O)	95/303/90:10 (THF/H_2_O)/–	519 (H_2_O/THF)/–	[330]
Ceramic	Zeolite NaA ^k^	8	PV (THF/H_2_O)	430/318/93:7 (THF/H_2_O)/–	1240 (H_2_O/THF)/–	[331]
Ceramic	Zeolite NaA ^k^	8	PV (AC ^l^/H_2_O)	130/313/97:3 (AC/H_2_O)/–	50 (H_2_O/AC)/–	[331]
PVA	0.5 wt % CNT(CS) ^m^	80	PV (benzene/cyclohexane)	53.0 ± 0.5/323/50/50 (wt/wt) (benzene/cyclohexane)/–	23.1 ± 0.4 (benzene/cyclohexane)/–	[259]
PVA	1.0 wt % CNT(CS)	80	PV (benzene/cyclohexane)	60.8 ± 0.6/323/50/50 (wt/wt) (benzene/cyclohexane)/–	30.4 ± 0.8 (benzene/cyclohexane)/–	[259]
PVA	1.5 wt % CNT(CS)	80	PV (benzene/cyclohexane)	67.3 ± 1.0/323/50/50 (wt/wt) (benzene/cyclohexane)/–	37.6 ± 0.5 (benzene/cyclohexane)/–	[259]
PVA	2.0 wt % CNT(CS)	80	PV (benzene/cyclohexane)	65.9 ± 1.2/323/50/50 (wt/wt) (benzene/cyclohexane)/–	53.4 ± 0.4 (benzene/cyclohexane)/–	[259]
PVA	2.5 wt % CNT(CS)	80	PV (benzene/cyclohexane)	58.9 ± 0.9/323/50/50 (wt/wt) (benzene/cyclohexane)/–	46.4 ± 0.7 (benzene/cyclohexane)/–	[259]

^a^ poly(tetrafluoroethylene), ^b^ aromatic PAm polymerized by interfacial reaction between aqueous m-phenyl diamine and organic trimesoyl chloride solutions, ^c^ PV, ^d^ isopropyl alcohol, ^e^ polyvinyl alcohol, ^f^ *γ*-aminopropyl-triethoxysilane, ^g^ chitosan, ^h^ tetrahydrofuran, ^j^ dioxan, ^k^ sodium form of zeolite A, ^l^ acetone, and ^m^ carbon nanotube (chitosan).

**Table 3:** Literature study of inorganic NCMs towards PV performance prepared via solution casting and solvent evaporation method (SCSE).

Polymeric Network	Inorganic Nanocomposite	Flux (g m^−2^ h^−1^)/SF(–)(x/y)/Temperature (K)	Feed Composition (wt %/wt %)	Reference
PDMS ^a^	TMS-H-silica ^b^	135/33 (IPA ^c^/H_2_O)/298	IPA/H_2_O (4/96)	[332]
PDMS	TMS-H-silica	405/32 (IPA/H_2_O)/323	IPA/H_2_O (4/96)	[332]
PDMS	TMS-H-silica	117/28 (EtOH/H_2_O)/303	EtOH/H_2_O (4/96)	[332]
PDMS	TMS-H-silica	329/26 (EtOH/H_2_O)/323	EtOH/H_2_O (4/96)	[332]
CS ^e^	3 wt % Silica	450/639 (EtOH/H_2_O)/343	EtOH/H_2_O (90/10)	[153]
CS	5 wt % Silica	410/919 (EtOH/H_2_O)/343	EtOH/H_2_O (90/10)	[153]
CS	10 wt % Silica	450/735 (EtOH/H_2_O)/343	EtOH/H_2_O (90/10)	[153]
CS	20 wt % Silica	320/919 (EtOH/H_2_O)/343	EtOH/H_2_O (90/10)	[153]
CS	30 wt % Silica	410/1102 (EtOH/H_2_O)/343	EtOH/H_2_O (90/10)	[153]
PVA ^f^	APTEOS ^g^	35.5/537/(H_2_O/EtOH)/323	EtOH/H_2_O (85/15)	[333]
PVA	BTEE ^h^	227/70 (H_2_O/EtOH)/323	EtOH/H_2_O (85/15)	[339]
P84 *co*-polyimide ^i^	Zeolite-5A	40/4200 (H_2_O/IPA ^c^)/323	IPA/H_2_O (90/10)	[123]
PVA ^f^	KA zeolites ^j^	179/410 (H_2_O/IPA)/323	IPA/H_2_O (90/10)	[272]
PVA	NaA zeolites ^k^	140/1170 (H_2_O/IPA)/323	IPA/H_2_O (90/10)	[272]
PVA	CaA zeolites ^l^	157/1170 (H_2_O/IPA)/323	IPA/H_2_O (90/10)	[272]
PVA	NaX zeolites	170/516 (H_2_O/IPA)/323	IPA/H_2_O (90/10)	[272]
PVA	KA zeolites ^j^	179/410 (H_2_O/IPA)/323	IPA/H_2_O (80/20)	[272]
PVA	NaA zeolites ^k^	183/328 (H_2_O/IPA)/323	IPA/H_2_O (80/20)	[272]
PVA	CaA zeolites ^l^	190/233 (H_2_O/IPA)/323	IPA/H_2_O (80/20)	[272]
PVA	NaX zeolites	216/233 (H_2_O/IPA)/323	IPA/H_2_O (80/20)	[272]
P84 *co*-polyimide ^i^	Zeolite-13X	110/2700 (H_2_O/IPA)/333	IPA/H_2_O (90/10)	[274]
PBI ^m^	ZIF-8 ^n^	103/1686 (H_2_O/IPA)/333	IPA/H_2_O (85/15)	[146]
PBI	ZIF-8	81/341.7 (H_2_O/n-BuOH)/333	n-BuOH/H_2_O (85/15)	[146]
PBI	ZIF-8	992/10 (H_2_O/EtOH)/333	EtOH/H_2_O (85/15)	[146]
PVA ^f^	5 wt % ZIF-8	868/132 (H_2_O/IPA)/303	IPA/H_2_O (90/10)	[275]
PVA	7.5 wt % ZIF-8	952/91 (H_2_O/IPA)/293	IPA/H_2_O (90/10)	[275]
PDMS ^a^	Fumed silica (A200)	200/19 (EtOH/H_2_O)/313	EtOH/H_2_O (5/95)	[340]
PDMS	Fumed silica	–/7 (EtOH/H_2_O)/313	EtOH/H_2_O (5/95)	[340]
PDMS	Carbon black	–/7 (EtOH/H_2_O)/308	EtOH/H_2_O (5/95)	[340]
PDMS	Zeolite Y	750/5 (EtOH/H_2_O)/308	EtOH/H_2_O (5/95)	[340]
PDMS	Silicalite-1 ^o^	51/17 (EtOH/H_2_O)/295.5	EtOH/H_2_O (5/95)	[340]
Matrimid ^p^	MgO	4500/1800 (H_2_O/IPA ^c^)/373	IPA/H_2_O (90/10)	[341]
PVA	PMA ^q^	36/29991 (IPA/H_2_O)/323	IPA/H_2_O (90/10)	[273]
PVA	Silicalite-1 ^o^	69/2241 (IPA/H_2_O)/323	IPA/H_2_O (90/10)	[273]
PVA	Sulfated zirconia	183/86 (H_2_O/EtOH)/323	EtOH/H_2_O (80/20)	[342]
PVA	H-ZSM5 ^r^	182/46 (H_2_O/EtOH)/323	EtOH/H_2_O (85/15)	[343]
Matrimid ^p^	Cyclodextrin	50/>5000 (H_2_O/IPA ^c^)/323	IPA/H_2_O (86/14)	[344]
Matrimid	Zeolite-4A	21/>5000 (H_2_O/IPA)/303	IPA/H_2_O (90/10)	[345]
Matrimid	Cu_3_(BTC)_2_^s^	400/245 (H_2_O/IPA)/323	IPA/H_2_O (90/10)	[346]
Polyimide ^t^	Zeolite-13X	150/272 (H_2_O/IPA)/323	IPA/H_2_O (90/10)	[276]
NaCMC ^u^/PVA ^f^	Zeolite-13X	121/5118 (H_2_O/EtOH)/308	EtOH/H_2_O (90/10)	[277]
PDMS ^a^	SiO_2_	807/13 (H_2_O/EtOH)/333	EtOH/H_2_O (5/95)	[347]
PDMS	ZIF-8 ^n^	1229/10 (H_2_O/EtOH)/333	EtOH/H_2_O (5/95)	[347]
PDMS	SiO_2_	1693/9 (H_2_O/n-BuOH)/333	n-BuOH/H_2_O (5/95)	[347]
PDMS	ZIF-8 ^n^	1743/30 (H_2_O/n-BuOH)/333	n-BuOH/H_2_O (5/95)	[347]
PDMS	PZSNTs ^v^	–/10 (EtOH/H_2_O)/313	EtOH/H_2_O (10/90)	[222]
PDMS	[Cu^II^_2_(bza)_4_(pyz)]_n_	23/2 (EtOH/H_2_O)/308	EtOH/H_2_O (5/95)	[348]
PDMS	Silicalite-1 ^o^	–/16 (EtOH/H_2_O)/308	EtOH/H_2_O (6/94)	[349]
CS ^e^	IDD ^w^	184/125 (IPA/H_2_O)//298	IPA/H_2_O (70/30)	[350]

^a^ Polydimethylsiloxane, ^b^ trimethylsilanol hydrophobized silica, ^c^ isopropyl alcohol, ^e^ chitosan, ^f^ polyvinyl alcohol, ^g^ *γ*-aminopropyl-triethoxysilane, ^h^ 1,2-bis(triethoxysilyl)ethane, ^i^ copolyimide of 3,3′,4,4′-benzophenone tetracarboxylic dianhydride and 5(6)-amino-1-(40-aminophenyl-1,3-trimethylindane, ^j^ potassium exchanged zeolite A, ^k^ sodium form of zeolite A, ^l^ calcium form of zeolite A, ^m^ polybenzimidazole, ^n^ zeolitic imidazolate framework, ^o^ type of zeolite, ^p^ copolyimide of 3,3′,4,4′-benzophenone tetracarboxylic dianhydride and 80% methylphenylene-diamine +20% methylene diamine, ^q^ phosphomolybdic acid, ^r^ zeolite socony mobil–5, ^s^ 1,3,5-benzene tricarboxylate, ^t^ polymer of 2,2-bis(4-(4-aminophenoxy)phenyl)propamine and bicyclo[2.2.2]oct-7-ene-2,3,5,6-tetracarboxylic dianhydride, ^u^ sodium carboxymethylcellulose, ^v^ polyphosphazene nanotubes, ^w^ 4-isocyanato-40-(3,30-dimethyl-2,4-dioxo azetidino)diphenylmethane.

## References

[1] Singha N.R., Karmakar M., Mahapatra M., Mondal H., Dutta A., Roy C., Chattopadhyay P.K. (2017). Systematic synthesis of pectin-*g*-(sodium acrylate-*co*-*N*-isopropylacrylamide) interpenetrating polymer network for mere/synergistic superadsorption of dyes/M(II): Comprehensive determination of physicochemical changes in loaded hydrogels. Polym. Chem..

[2] Singha N.R., Das P., Ray S.K. (2013). Recovery of pyridine from water by pervaporation using filled and crosslinked EPDM membranes. J. Ind. Eng. Chem..

[3] Baker R.W. (2012). Membrane Technology and Applications.

[4] Ho W.S.W., Sirkar K.K. (1992). Membrane Handbook.

[5] Bodzek M., Bohdziewicz J., Konieczny K. (1997). Techniki Membranowe W Ochronie Srodowiska.

[6] Mulder M.H.V. (1991). Basic Principles of Membrane Technology.

[7] Feng X., Huang R.Y.M. (1997). Liquid separation by membrane pervaporation: A review. Ind. Eng. Chem. Res..

[8] Slater C.S. (1991). A review of: “Pervaporation membrane separation processes”. Sep. Purif. Methods.

[9] Smitha B., Suhanya D., Sridhar S., Ramakrishna M. (2004). Separation of organic–organic mixtures by pervaporation—A review. J. Membr. Sci..

[10] Jiang L.Y., Wang Y., Chung T.S., Qiao X.Y., Lai J.Y. (2009). Polyimides membranes for pervaporation and biofuels separation. Prog. Polym. Sci..

[11] Das P., Ray S.K., Kuila S.B., Samanta H.S., Singha N.R. (2011). Systematic choice of crosslinker and filler for pervaporation membrane: A case study with dehydration of isopropyl alcohol-water mixtures by polyvinyl alcohol membranes. Sep. Purif. Technol..

[12] Mujiburohman M., Feng X. (2007). Permselectivity, solubility and diffusivity of propyl propionate/water mixtures in poly(ether block amide)membranes. J. Membr. Sci..

[13] Singha N.R., Kuila S.B., Das P., Ray S.K. (2009). Separation of toluene–methanol mixtures by pervaporation using crosslink IPN membranes. Chem. Eng. Process. Process Intensif..

[14] Singha N.R., Ray S.K. (2012). Removal of pyridine from water by pervaporation using crosslinked and filled natural rubber membranes. J. Appl. Polym. Sci..

[15] Singha N.R., Ray S., Ray S.K., Koner B.B. (2011). Removal of pyridine from water by pervaporation using filled SBR membranes. J. Appl. Polym. Sci..

[16] Pereira C.C., Claudio P.R., Ronaldo N., Cristiano P.B. (2006). Pervaporative recovery of volatile aroma compounds from fruit juices. J. Membr. Sci..

[17] Mahapatra M., Karmakar M., Mondal B., Singha N.R. (2016). Role of ZDC/S ratio for pervaporative separation of organic liquids through modified EPDM membranes: Rational mechanistic study of vulcanization. RSC Adv..

[18] Kahlenberg L. (1906). On the nature of the process of osmosis and osmotic pressure with observation concerning dialysis. J. Phys. Chem..

[19] Kober P.A. (1917). Pervaporation, perstillation and percrystallization. J. Am. Chem. Soc..

[20] Villaluenga J.P.G., Tabe-Mohammadi A. (2000). A review on the separation of benzene/cyclohexane mixtures by pervaporation processes. J. Membr. Sci..

[21] Vane L.M. (2005). A review of pervaporation for product recovery from biomass fermentation processes. J. Chem. Technol. Biotechnol..

[22] Aptel P., Challard N., Cuny J., Neel J. (1976). Application of the pervaporation process to separate azeotropic mixtures. J. Membr. Sci..

[23] Cheng X., Pan F., Wang M., Li W., Song Y., Liu G., Yang H., Gao B., Wu H., Jiang Z. (2017). Hybrid membranes for pervaporation separations. J. Membr. Sci..

[24] Ong Y.K., Shi G.M., Le N.L., Tang Y.P., Zuo J., Nunes S.P., Chung T. (2016). Recent membrane development for pervaporation processes. Prog. Polym. Sci..

[25] Hwang S., Kammermyer K. (1975). Membranes in Separation.

[26] Loeb S., Sourirajan S. (1964). High Flow Porous Membranes for Separating Water from Saline Solutions. U.S. Patent.

[27] Binning R.C., James F.E. (1958). Permeation. A new commercial separation tool. Perot. Eng..

[28] Binning R.C., Jennings J.F., Martin E.C. (1962). Process for Removing Water from Organic Chemicals. U.S. Patent.

[29] Tusel G.F., Brüschke H.E.A. (1985). Use of pervaporation systems in the chemical industry. Desalination.

[30] Nunes S.P., Peinemann K.V. (2006). Membrane Technology: In the Chemical Industry.

[31] Jonquieres A., Clement R., Lochon P., Neel J., Dresch M., Chreticn B. (2002). Industrial state-of-the-art of pervaporation and vapour permeation in the western countries. J. Membr. Sci..

[32] Dong Y.Q., Zhang L., Shen J.N., Song M.Y., Chen H.L. (2006). Preparation of poly(vinyl alcohol)-sodium alginate hollow fiber composite membranes and pervaporation dehydration characterization of aqueous alcohol mixtures. Desalination.

[33] Lipnizki F., Field R.W., Ten P.-K. (1999). Pervaporation-based hybrid process: A review of process design, applications and economics. J. Membr. Sci..

[34] Baker R.W. (2010). Research needs in the membrane separation industry: Looking back, looking forward. J. Membr. Sci..

[35] Liu C., Kulprathipanja S., Hillock A.M.W., Husain S., Koros W.J. (2008). Recent progress in mixed-matrix membranes. Advanced Membrane Technology and Applications.

[36] Michaels A.S., Baddour R.F., Bixler H.J., Choo C.Y. (1962). Conditioned polyethylene as a permselective membrane separation of isomeric xylenes. Ind. Eng. Chem. Process. Des. Dev..

[37] Schrodt V.N., Sweeny R.F., Rose A. (1963). Division of industrial and engineering chemistry Los Angeles, March. Future Industrial Prospects of Membrane Processes.

[38] Long R.B. (1965). Liquid permeation through plastic films. Ind. Eng. Chem. Fundam..

[39] Baker R.W., Wijmans J.G., Huang Y. (2010). Permeability, permeance and selectivity: A preferred way of reporting pervaporation performance data. J. Membr. Sci..

[40] Wijmans J.G. (2003). Process performance = Membrane properties + Operating conditions. J. Membr. Sci..

[41] Sampranpiboon P., Jiraratananon R., Uttapap D., Feng X., Huang R.Y.M. (2000). Pervaporation separation of ethyl butyrate and isopropanol with polyether block amide (PEBA) membranes. J. Membr. Sci..

[42] Sourirajan S., Shiyao B., Matsuura T. An approach to membrane separation by pervaporation. Proceedings of the Second International Conference on Pervaporation Processes in Chemical Industry.

[43] Sourirajan S., Shiyao B., Matsuura T. (1985). Reverse Osmosis and UF/Process Principles.

[44] Wijmans J.G., Baker R.W. (1995). The solution-diffusion model: A review. J. Membr. Sci..

[45] Glasstone S., Laidler K.J., Eyring H. (1941). The Theory of Rate Processes.

[46] Peppas N.A., Reinhart C.T. (1983). Solute diffusion in swollen membranes. Part I. A new theory. J. Membr. Sci..

[47] Ji W., Hwang S.-T. (1994). Modeling of multicomponent pervaporation for removal of volatile organic compounds from water. J. Membr. Sci..

[48] Blume I., Wijmans J.G., Baker R.W. (1990). Separation of dissolved organics from water by pervaporation. J. Membr. Sci..

[49] González B.G., Uribe I.O. (2001). Mathematical modeling of the pervaporative separation of methanol−methylterbutyl ether mixtures. Ind. Eng. Chem. Res..

[50] Shao P., Huang R.Y.M. (2007). Polymeric membrane pervaporation. J. Membr. Sci..

[51] Casado C., Urtiaga A., Gorri D., Ortiz I. (2005). Pervaporative dehydration of organic mixtures using a commercial silica membrane: Determination of kinetic parameters. Sep. Purif. Technol..

[52] Barrer R.M., Barrie J.A., Rogers M.G. (1963). Heterogenous membranes: Diffusion in filled rubber. J. Polym. Sci. A.

[53] Chung T.S., Chan S.S., Wang R., Lu Z., He C. (2003). Characterization of permeability and sorption in Matimid/C_60_ mixed matrix membranes. J. Membr. Sci..

[54] Vijay Y.K., Kulshrestha V., Awasthi K., Acharya N.K., Jain A., Singh M., Dolia S.N., Khan S.A., Avasthi D.K. (2006). Characterization of nanocomposite polymeric membrane. J. Polym. Res..

[55] Merkel T.C., Freeman B.D., Spontak R.J., He Z., Pinnau I., Meakin P., Hill A.J. (2003). Sorption, transport and structural evidence for enhanced free volume in poly(4-methyl-2-pentyne)/fumed silica NCMs. Chem. Mater..

[56] Gomes D., Nunes S.P., Peinemann K.V. (2005). Membranes for gas separation based on poly (1-trimetylsilyl-1-propyne)-silica nanocomposites. J. Membr. Sci..

[57] Saga S., Matsumoto H., Saito K., Minagawa M., Tanioka A. (2008). Polyelectrolyte membranes based on hydrocarbon polymer containing fullerene. J. Power Sources.

[58] Labille J., Masion A., Ziarelli F., Rose J., Brant J., Villieras F., Pelletier M., Borschneck D., Wiesner M.R., Bottero J.Y. (2009). Hydration and dispersion of C-60 in aqueous systems: The nature of water-fullerene interactions. Langmuir.

[59] Suzuki T., Yamada Y., Sakai J. (2006). Gas transport properties of ODPA-TAPOB hyperbranched polyimide-silica hybrid membranes. High Perform. Polym..

[60] Bruggeman D.A.G. (1935). Berechnung verschiedener physikalischer Konstanten von heterogenen Substanzen. Ann. Phys..

[61] Bouma R.H.B., Checchetti A., Chidichimo G., Drioli E. (1997). Permeation through a heterogeneous membrane: The effect of the dispersed phase. J. Membr. Sci..

[62] Vu D.Q., Koros W.J., Miller S.J. (2003). Mixed matrix membranes using carbon molecular sieves II. Modeling permeation behavior. J. Membr. Sci..

[63] Shariati A., Omidkhah M., Pedram M.Z. (2012). New permeation models for nanocomposite polymeric membranes filled with nonporous particles. Chem. Eng. Res. Des..

[64] Yeom C.K., Lee K.-H. (1997). A study on desorption resistance in pervaporation of single component through dense membranes. J. Appl. Polym. Sci..

[65] Rautenbach R., Hommerich U. (1998). Experimental study of dynamic mass-transfer effects in pervaporation. AIChE J..

[66] Ray S., Ray S.K. (2006). Effect of copolymer type and composition on separation characteristics of pervaporation membranes-A case study with separation of acetone-water mixtures. J. Membr. Sci..

[67] Cong H., Radosz M., Towler B.F., Shen Y. (2007). Polymer-inorganic NCMs for gas separation. Sep. Purif. Technol..

[68] Ray S., Ray S.K. (2006). Separation of organic mixtures by pervaporation using crosslinked and filled rubber membranes. J. Membr. Sci..

[69] Chung T.S., Jiang L.Y., Li Y., Kulprathipanja S. (2007). Mixed matrix membranes (MMMs) comprising organic polymers with dispersed inorganic fillers for gas separation. Prog. Polym. Sci..

[70] Kim J.H., Lee Y.M. (2001). Gas permeation properties of poly(amide-6-*b*-ethylene oxide)-silica hybrid membranes. J. Membr. Sci..

[71] Vu D.Q., Koros W.J., Miller S.J. (2003). Mixed matrix membranes using carbon molecular sieves: I. Preparation and experimental results. J. Membr. Sci..

[72] Huang Z., Shi Y., Wen R., Guo Y., Su J., Matsuura T. (2006). Multilayer poly(vinyl alcohol)-zeolite 4A composite membranes for ethanol dehydration by means of pervaporation. Sep. Purif. Technol..

[73] Ciobanu G., Gabriela C., Octavian C. (2008). Structure of mixed matrix membranes made with SAPO-5 zeolite in polyurethane matrix. Microporous Mesoporous Mater..

[74] Albo J., Hagiwara H., Yanagishita H., Ito K., Tsuru T. (2014). Structural characterization of thin-film PAm reverse osmosis membranes. Ind. Eng. Chem. Res..

[75] Albo J., Wang J., Tsuru T. (2014). Gas transport properties of interfacially polymerized PAm composite membranes under different pre-treatments and temperatures. J. Membr. Sci..

[76] Georgakilas V., Bourlinos A., Gournis D., Tsoufis T., Trapalis C., Mateo-Alonso A., Prato M. (2008). Multipurpose organically modified carbon nanotubes: From functionalization to nanotube composites. J. Am. Chem. Soc..

[77] Choi J.H., Jegal J., Kim W.N. (2007). Modification of performances of various membranes using MWNTs as a modifier. Macromol. Symp..

[78] Sahoo N.G., Rana S., Cho J.W., Li L., Chan S.H. (2010). Polymer nanocomposites based on functionalized carbon nanotubes. Prog. Polym. Sci..

[79] Skoulidas A.I., Ackerman D.M., Johnson J.K., Sholl D.S. (2002). Rapid transport of gases in carbon nanotubes. Phys. Rev. Lett..

[80] Majumder M., Chopra N., Andrews R., Hinds B.J. (2005). Enhanced flow in carbon nanotubes. Nature.

[81] Holt J.K., Park H.G., Wang Y., Stadermann M., Artyukhin A.B., Grigoropoulos C.P., Noy A., Bakajin O. (2006). Fast mass transport through sub-2-nanometer carbon nanotubes. Science.

[82] Kim S., Jinschek J.R., Chen H., Sholl D.S., Marand E. (2007). Scalable fabrication of carbon nanotube/polymer NCMs for high flux gas transport. Nano Lett..

[83] Cong H., Zhang J., Radosz M., Shen Y. (2007). Carbon nanotube composite membranes of brominated poly(2,6-diphenyl-1,4-phenylene oxide) for gas separation. J. Membr. Sci..

[84] Choi J.H., Jegal J., Kim W.N., Choi H.S. (2009). Incorporation of multiwalled carbon nanotubes into poly(vinyl alcohol) membranes for use in the pervaporation of water/ethanol mixtures. J. Appl. Polym. Sci..

[85] Murali R.S., Sridhar S., Sankarshana T., Ravikumar Y.V.L. (2010). Gas permeation behavior of Pebax-1657 NCM incorporated with multiwalled carbon nanotubes. Ind. Eng. Chem. Res..

[86] Shirazi Y., Tofighy M.A., Mohammadi T. (2011). Synthesis and characterization of carbon nanotubes/poly vinyl alcohol NCMs for dehydration of isopropanol. J. Membr. Sci..

[87] Shao L., Samseth J., Hagg M.B. (2009). Crosslinking and stabilization of NP filled PMP NCMs for gas separations. J. Membr. Sci..

[88] Tancharernrat T., Rempel G.L., Prasassarakich P. (2014). Preparation of styrene butadiene copolymer–silica nanocomposites via differential microemulsion polymerization and NR/SBR–SiO_2_ membranes for pervaporation of water–ethanol mixtures. Chem. Eng..

[89] Yang D., Li J., Jiang Z., Lu L., Chen X. (2009). Chitosan/TiO_2_ nanocomposite pervaporation membranes for ethanol dehydration. Chem. Eng. Sci..

[90] Chapman P.D., Oliveira T., Livingston A.G., Li K. (2008). Membranes for the dehydration of solvents by pervaporation. J. Membr. Sci..

[91] Urtiaga A.M., Gorri E.D., Gómez P., Casado C., Ibáñez R., Ortiz I. (2007). Pervaporation technology for the dehydration of solvents and raw materials in the process industry. Drying Technol..

[92] Kang M., Choi Y., Moon S. (2002). Water-swollen cation-exchange membranes prepared using poly(vinyl alcohol) (PVA)/poly(styrene sulfonic acid-co-maleic acid) (PSSA-MA). J. Membr. Sci..

[93] Huang R.Y.M., Yeom C.K. (1991). Pervaporation separation of aqueous mixtures using crosslinked polyvinyl alcohol membranes. III. Permeation of acetic acid-water mixtures. J. Membr. Sci..

[94] Chiang W.Y., Hu C.M. (1991). Separation of liquid mixtures by using polymer membranes. I. Water–alcohol separation by pervaporation through PVA-*g*-MMA/MA membrane. J. Appl. Polym. Sci..

[95] Kang Y.S., Lee S.W., Kim V.Y., Shim J.S. (1990). Pervaporation of water-ethanol mixtures through crosslinked and surface modified poly(vinyl alcohol) membranes. J. Membr. Sci..

[96] Wu L.G., Zhu C.-L., Liu M. (1994). Study of a new pervaporation membrane Part I. Preparation and characteristics of the new membrane. J. Membr. Sci..

[97] Zhang S., Zou Y., Wei T., Mua C., Liu X., Tong Z. (2017). Pervaporation dehydration of binary and ternary mixtures of n-butyl acetate, n-butanol and water using PVA-CS blended membranes. Sep. Purif. Technol..

[98] Vauclair C., Tarjus H., Schaetzel P. (1997). Permselective properties of PVA-PAA blended membrane used for dehydration of fusel oil by pervaporation. J. Membr. Sci..

[99] Burshe M.C., Sawant S.B., Joshi J.B., Pangarkar V.G. (1997). Sorption and permeation of binary water-alcohol systems through PVA membranes crosslinked with multifunctional crosslinking agents. Sep. Purif. Technol..

[100] Wang X.-P. (2000). Modified alginate composite membranes for the dehydration of acetic acid. J. Membr. Sci..

[101] Guan H.-M., Chung T.-S., Huang Z., Chng M.L., Kulprathipanja S. (2006). Poly(vinyl alcohol) multilayer mixed matrix membranes for the dehydration of ethanol–water mixture. J. Membr. Sci..

[102] Peters T.A., Poeth C.H.S., Benes N.E., Buijs H.C.W.M., Vercauteren F.F., Keurentjes J.T.F. (2006). Ceramic-supported thin PVA pervaporation membranes combining high flux and high selectivity; contradicting the flux-selectivity paradigm. J. Membr. Sci..

[103] Yi C., Wang Z., Li M., Wang J., Wang S. (2006). Facilitated transport of CO_2_ through polyvinylamine/polyethlene glycol blend membranes. Desalination.

[104] Liu J., Bernstein R. (2017). High-flux thin-film composite polyelectrolyte hydrogel membranes for ethanol dehydration by pervaporation. J. Membr. Sci..

[105] Huang R.Y.M., Moreira H., Nufarforizo R., Huang Y.M. (1998). Pervaporation separation of acetic acid-water mixtures using modified membranes. I. Blended polyacrylic acid (PAA)-nylon 6 membranes. J. Appl. Polym. Sci..

[106] Aptel P., Cuny J., Jozefonvicz J., Morel G., Neel J. (1974). Liquid transport through membranes prepared by grafting of polar monomers onto poly(tetrafluoroethylene) films. III. Steady-state distribution in membrane during pervaporation. J. Appl. Polym. Sci..

[107] Hirotsu T., Isayama M. (1989). Water-ethanol separation by pervaporation through plasma-graft-polymerized membranes of 2-hydroxyethyl methacrylate with acrylic acid or methacrylic acid. J. Membr. Sci..

[108] Ray S.K., Sawant S.B., Joshi J.B., Pangarkar V.G. (1998). Dehydration of acetic acid by pervaporation. J. Membr. Sci..

[109] Ray S., Ray S.K. (2005). Dehydration of acetic acid, alcohols, and acetone by pervaporation using acrylonitrile-maleic anhydride copolymer membrane. Sep. Sci. Technol..

[110] Bhat S.D., Aminabhavi T.M. (2007). Pervaporation separation using sodium alginate and its modified membranes-A review. Sep. Purif. Rev..

[111] Moulik S., Nazia S., Vani B., Sridhar S. (2016). Pervaporation separation of acetic acid/water mixtures through sodium alginate/polyaniline polyion complex membrane. Sep. Sci. Technol..

[112] Lee Y.M., Nam S.Y., Ha S.Y. (1999). Pervaporation of water/isopropanol mixtures through polyaniline membranes doped with poly(acrylic acid). J. Membr. Sci..

[113] Xu S., Liu L., Wang Y. (2017). Network cross-linking of polyimide membranes for pervaporation dehydration. Sep. Purif. Technol..

[114] Liao Y.L., Hu C.-C., Lai J.-Y., Liu Y.-L. (2017). Crosslinked polybenzoxazine based membrane exhibiting in-situ self-promoted separation performance for pervaporation dehydration on isopropanol aqueous solutions. J. Membr. Sci..

[115] Kurşuna F., Işıklan N. (2016). Development of thermo-responsive poly(vinyl alcohol)-*g*-poly(*N*-isopropylacrylamide) copolymeric membranes for separation of isopropyl alcohol/water mixtures via pervaporation. J. Ind. Eng. Chem..

[116] Devi D.A., Smitha B., Sridhar S., Aminabhavi T.M. (2006). Pervaporation separation of dimethylformamide/water mixtures through poly(vinyl alcohol)/poly(acrylic acid) blend membranes. Sep. Purif. Technol..

[117] Ray S., Ray S.K. (2006). Pervaporative dehydration of dimethyl formamide (DMF) by crosslinked copolymer membranes. Ind. Eng. Chem. Res..

[118] Roy S., Thongsukmak A., Tang J., Sirkar K.K. (2011). Concentration of aqueous hydrogen peroxide solution by pervaporation. J. Membr. Sci..

[119] Lee Y.M., Oh B.-K. (1995). Dehydration of water-pyridine mixture through poly(acrylonitrile-*co*-acryclic acid) membrane by pervaporation. J. Membr. Sci..

[120] Wei X.-Z., Liu X.-F., Zhu B.K., Xu Y.-Y. (2009). Membranes of crosslinked hyperbranch polymers and their pervaporation properties. Desalination.

[121] Wang Y., Chung T.S., Neo B.W., Gruender M. (2011). Processing and engineering of pervaporation dehydration of ethylene glycol via dual-layer polybenzimidazole (PBI)/polyetherimide (PEI) membranes. J. Membr. Sci..

[122] Kujawski J.K., Kujawski W.M., Sondej H., Jarzynka K., Kujawska A., Bryjak M., Rynkowska E., Knozowska K., Kujawa J. (2017). Dewatering of 2,2,3,3-tetrafluoropropan-1-ol by hydrophilic pervaporation with poly(vinyl alcohol) based Pervap^TM^ membranes. Sep. Purif. Technol..

[123] Adoor S.G., Prathab B., Manjeshwar L.S., Aminabhavi T.M. (2007). Mixed matrix membranes of sodium alginate and poly(vinyl alcohol) for pervaporation dehydration of isopropanol at different temperatures. Polymer.

[124] Magalad V.T., Gokavi G.S., Raju K.V.S.N., Aminabhavi T.M. (2010). Mixed matrix blend membranes of poly(vinyl alcohol)–poly(vinyl pyrrolidone) loaded with phosphomolybdic acid used in pervaporation dehydration of ethanol. J. Membr. Sci..

[125] Gao L., Alberto M., Gorgojo P., Szekely G., Budd P.M. (2017). High-flux PIM-1/PVDF thin film composite membranes for 1-butanol/water pervaporation. J. Membr. Sci..

[126] Li S., Qiu S., Yu B., Tang G., Xing W., Hu Y. (2016). POSS-functionalized polyphosphazene nanotube: Preparation and effective reinforcement on UV-curable epoxy acrylate nanocomposite coatings. RSC Adv..

[127] Liu W., Li B., Cao R., Jiang Z., Yu S., Liu G., Wu H. (2011). Enhanced pervaporation performance of poly (dimethyl siloxane) membrane by incorporating titania microspheres with high silver ion loading. J. Membr. Sci..

[128] Magalad V.T., Gokavi G.S., Nadagouda M.N., Aminabhavi T.M. (2011). Pervaporation separation of water-ethanol mixtures using organic-inorganic NCMs. J. Phys. Chem. C.

[129] Casado-Coterillo C., Andrés F., Téllez C., Coronas J., Irabien Á. (2014). Synthesis and characterization of ETS-10/Chitosan NCMs for pervaporation. Sep. Sci. Technol..

[130] García-Cruz L., Casado-Coterillo C., Iniesta J., Montiel V., Irabien A. (2015). Preparation and characterization of novel chitosan-based mixed matrix membranes resistant in alkaline media. J. Appl. Polym. Sci..

[131] Adoor S.G., Manjeshwar L.S., Bhat S.D., Aminabhavi T.M. (2008). Aluminum-rich zeolite beta incorporated sodium alginate mixed matrix membranes for pervaporation dehydration and esterification of ethanol and acetic acid. J. Membr. Sci..

[132] Yang Y., Zhang H., Wang P., Zeng Q., Li J. (2007). The influence of nano-sized TiO_2_ fillers on the morphologies and properties of PSF UF membrane. J. Membr. Sci..

[133] Gong L., Zhang L., Wang N., Li J., Ji S., Guo H., Zhang G. (2014). In situ ultraviolet-light-induced TiO_2_ nanohybrid superhydrophilic membrane for pervaporation dehydration. Sep. Purif. Technol..

[134] Lokesh B.G., Rao K.S.V.K., Reddy K.M., Rao K.C., Rao P.S. (2008). Novel NCMs of sodium alginate filled with polyaniline-coated titanium dioxide for dehydration of 1,4-dioxane/water mixtures. Desalination.

[135] Aminabhavi T.M., Patil M.B., Bhat S.D., Halgeri A.B., Vijayalakshmi R.P., Kumar P. (2009). Activated charcoal-loaded composite membranes of sodium alginate in pervaporation separation of water-organic azeotropes. J. Appl. Polym. Sci..

[136] Singha N.R., Parya T.K., Ray S.K. (2009). Dehydration of 1,4-dioxane by perva-poration using filled and crosslinked polyvinyl alcohol membrane. J. Membr. Sci..

[137] Veerapur R.S., Patil M.B., Gudasi K.B., Aminabhavi T.M. (2008). Poly(vinylalcohol)-zeolite T mixed matrix composite membranes for pervaporation separation of water + 1,4-dioxane mixtures. Sep. Purif. Technol..

[138] Tripathi B.P., Kumar M., Saxena A., Shahi V.K. (2010). Bifunctionalized organic–inorganic charged NCM for pervaporation dehydration of ethanol. J. Colloid Interf. Sci..

[139] Shan L., Gong L., Fan H., Ji S., Zhang G. (2017). Spray-assisted biomineralization of a superhydrophilic water uptake layer for enhanced pervaporation dehydration. J. Membr. Sci..

[140] Narkkun T., Jenwiriyakula W., Amnuaypanich S. (2017). Dehydration performance of double-network poly(vinyl alcohol) NCMs (PVAs-DN). J. Membr. Sci..

[141] Jia Z. (2016). Metal-organic frameworks based mixed matrix membranes for pervaporation. Microporous Mesoporous Mater..

[142] Chen B.L., Yang Z.X., Zhu Y.Q., Xia Y.D. (2014). Zeoliticimidazolate framework materials: Recent progress in synthesis and applications. J. Mater. Chem. A.

[143] Li W.B., Yang Z.H., Zhang G.L., Fan Z., Meng Q., Shen C., Gao C.J. (2014). Stiff metal–organic framework–polyacrylonitrile hollow fiber composite membranes with high gas permeability. J. Mater. Chem. A.

[144] Yang L.J., Tang B.B., Wu P.Y. (2015). Metal–organic framework-graphene oxide composites: A facile method to highly improve the proton conductivity of PEMs operated under low humidity. J. Mater. Chem. A.

[145] Kang C.H., Lin Y.F., Huang Y.S., Tung K.L., Chang K.S., Chen J.T., Hung W.S., Lee K.R., Lai J.Y. (2013). Synthesis of ZIF-7/chitosan mixed-matrix mem-branes with improved separation performance of water/ethanol mixtures. J. Membr. Sci..

[146] Shi G.M., Yang T.X., Chung T.S. (2012). Polybenzimidazole (PBI)/zeoliticimidazolate frameworks (ZIF-8) mixed matrix membranes for pervaporation dehydration of alcohols. J. Membr. Sci..

[147] Ordonez M.J.C., Balkus K.J., Ferraris J.P., Musselman I.H. (2010). Molecular sieving realized with ZIF-8/Matrimid mixed-matrix membranes. J. Membr. Sci..

[148] Hua D., Ong Y.K., Wang Y., Yang T., Chung T.S. (2014). ZIF-90/P84 mixed matrix membranes for pervaporation dehydration of isopropanol. J. Membr. Sci..

[149] Zhu Y., Gupta K.M., Liu Q., Jiang J., Huang A. (2016). Synthesis and seawater desalination of molecular sieving zeolitic imidazolate framework membranes. Desalination.

[150] Gascón J., Freek K., Beatriz Z., Víctor S., Clara C., Joaquín C. (2012). Practical approach to zeolitic membranes and coatings: State of the art, opportunities, barriers and future perspectives. Chem. Mater..

[151] Yao J., Wang H. (2014). Zeolitic imidazolate framework composite membranes and thin films: Synthesis and applications. Chem. Soc. Rev..

[152] Amnuaypanich S., Patthana J., Phinyocheep P. (2009). Mixed matrix membranes prepared from natural rubber/poly(vinyl alcohol) semi-interpenetrating polymer network (NR/PVA semi-IPN) incorporating with zeolite 4A for the pervaporation dehydration of water-ethanol mixtures. Chem. Eng. Sci..

[153] Liu Y.L., Hsu C.Y., Su Y.H., Lai J.Y. (2005). Chitosan-silica complex membranes from sulfonic acid functionalized silica NPs for pervaporation dehydration of ethanol-water solutions. Biomacromolecules.

[154] Sunitha K., Rani K.Y., Moulik S., Satyanarayana S.V., Sridhar S. (2013). Separation of NMP/water mixtures by nanocomposite PEBA membrane: Part I. Membrane synthesis, characterization and pervaporation performance. Desalination.

[155] Boom J.P., Bargeman D., Strathmann H. (1994). Zeolite filled membranes for gas separation and pervaporation. Zeolite and related microporous materials: State of art. Stud. Surf. Sci. Catal..

[156] Dudek G., Gnus M., Turczyn R., Strzelewicz A., Krasowska M. (2014). Pervaporation with chitosan membranes containing iron oxide NPs. Sep. Purif. Technol..

[157] Olukman M., Şanlı O. (2015). A novel in situ synthesized magnetite containing acrylonitrile and 2-hydroxyethyl methacrylate grafted poly(vinyl alcohol) NCMs for pervaporation separation of acetone/water mixtures. Chem. Eng. Process. Process Intensif..

[158] Shi G.M., Chen H., Jean Y.C., Chung T.S. (2013). Sorption, swelling, and free volume of polybenzimidazole (PBI) and PBI/zeolitic imidazolate framework (ZIF-8) nano-composite membranes for PV. Polymer.

[159] Liu G., Jiang Z., Cao K., Nair S., Cheng X., Zhao J., Gomaa H., Wu H., Pan F. (2017). PV performance comparison of hybrid membranes filled with two-dimensional ZIF-L nanosheets and zero-dimensional ZIF-8 NPs. J. Membr. Sci..

[160] Adoor S.G., Rajineekanth V., Nadagouda M.N., Rao K.C., Dionysiou D.D., Aminabhavi T.M. (2013). Exploration of NCMs composed of phosphotungstic acid in sodium alginate for separation of aqueous–organic mixtures by PV. Sep. Purif. Technol..

[161] Liu Q., Noble R.D., Falconer J.L., Funke H.H. (1996). Organics/water separation by PV with a zeolite membrane. J. Membr. Sci..

[162] Bowen T.C., Noble R.D., Falconer J.L. (2004). Fundamentals and applications of PV through zeolite membranes. J. Membr. Sci..

[163] Xu D., Loo L.S., Wang K.A. (2010). PV performance of novelchitosan-POSS hybrid membranes: Effects of POSS and operatingconditions. J. Polym. Sci. Polym. Phys..

[164] Le N.L., Tang Y.P., Chung T.S. (2013). The development of high-performance6FDA NDA/DABA/POSS/Ultem^®^ dual-layer hollow fibers for ethanol dehydration via PV. J. Membr. Sci..

[165] Penkova A.V., Acquah S.F.A., Dmitrenko M.E., Sokolova M.P., Mikhailova M.E., Polyakov E.S., Ermakov S.S., Markelov D.A., Roizard D. (2016). Improvement of PV PVA membranes by the controlled incorporation of fullerenol NPs. Mater. Des..

[166] Friebe S., Geppert B., Steinbach F., Caro J. (2017). Novel MOF UiO-66 layer: A Highly oriented membrane with good selectivity and hydrogen permeance. ACS Appl. Mater. Interf..

[167] Sun H., Tang B., Wu P. (2017). Development of hybrid ultrafiltration membranes with improved water separation properties using modified super-hydrophilic metal-organic framework nanoparticles. ACS Appl. Mater. Interf..

[168] Armstrong M.R., Arredondo K.Y.Y., Liu C., Stevens J.E., Mayhob A., Shan B., Senthilnathan S., Balzer C.J., Mu B. (2015). UiO-66 MOF and poly(vinyl cinnamate) nanofiber composite membranes synthesized by a facile three-stage process. Ind. Eng. Chem. Res..

[169] Zhao Q., Qian J.W., Zhu C.X., An Q.F., Xu T.Q., Zheng Q., Song Y. (2009). A novel method for fabricating polyelectrolyte complex/inorganic nanohybrid membranes with high isopropanol dehydration performance. J. Membr. Sci..

[170] Ong Y.T., Ahmad A.L., Zein S.H.S., Sudesh K., Tan S.H. (2011). Poly(3-hydroxybutyrate)-functionalised multi-walled carbon nanotubes/chitosan green NCMs and their application in pervaporation. Sep. Purif. Technol..

[171] Yeang Q.Y., Zein S.H.S., Sulong A.B., Tan S.H. (2013). Comparison of the pervaporation performance of various types of carbon nanotube-based nanocomposites in the dehydration of acetone. Sep. Purif. Technol..

[172] Panahian S., Raisi A., Aroujalian A. (2015). Multilayer mixed matrix membranes containing modified-MWCNTs for dehydration of alcohol by pervaporation process. Desalination.

[173] Qiu S., Wu L., Shi G., Zhang L., Chen H., Gao C. (2010). Preparation and pervaporation property of chitosan membrane with functionalized multiwalled carbon nanotubes. Ind. Eng. Chem. Res..

[174] Hua S.Y., Zhang Y., Lawless D., Feng X. (2012). Composite membranes comprising of polyvinylamine-poly(vinyl alcohol) incorporated with carbon nanotubes for dehydration of ethylene glycol by pervaporation. J. Membr. Sci..

[175] Suk J.W., Piner R.D., An J., Ruoff R.S. (2010). Mechanical properties of monolayer graphene oxide. ACS Nano.

[176] Saxena S., Tyson T.A., Negusse E. (2010). Investigation of the local structure of graphene oxide. J. Phys. Chem. Lett..

[177] Goh P.S., Ismail A.F. (2015). Graphene-based nanomaterial: The state-of-the-art material for cutting edge desalination technology. Desalination.

[178] Mahmoud K.A., Mansoor B., Mansour A., Khraisheh M. (2015). Functional graphene nanosheets: The next generation membranes for water desalination. Desalination.

[179] Dave H.K., Nath K. (2016). Graphene oxide incorporated novel polyvinyl alcohol composite membrane for pervaporative recovery of acetic acid from vinegar waste water. J. Water Process Eng..

[180] Salehian P., Chung T.S. (2017). Thermally treated ammonia functionalized graphene oxide/polyimide membranes for pervaporation dehydration of isopropanol. J. Membr. Sci..

[181] Zhao J., Zhu Y., He G., Xing R., Pan F., Jiang Z., Zhang P., Cao X., Wang B. (2016). Incorporating zwitterionic graphene oxides into sodium alginate membrane for efficient water/alcohol separation. ACS Appl. Mater. Interf..

[182] Tang Y.P., Chan J.X., Chung T.S., Weber M., Staudt C., Maletzko C. (2015). Simultaneously covalent and ionic bridging towards antifouling of GO-imbedded nanocomposite hollow fiber membranes. J. Mater. Chem. A.

[183] Hua D., Rai R.K., Zhang Y., Chung T.S. (2017). Aldehyde functionalized graphene oxide frameworks as robust membrane materials for pervaporative alcohol dehydration. Chem. Eng. Sci..

[184] Watson J.M., Payne P.A. (1990). A study of organic compound pervaporation through silicone rubber. J. Membr. Sci..

[185] Blume J., Schwering F.J.F., Mulder M.H.V., Smolders C.A., Backish R. (1989). Sorption and Permeation properties of poly(dimethyl siloxane) films. Proceedings of the Fourth International Conference on Pervaporation Processes in the Chemical Industry.

[186] Netke S.A., Sawant S.B., Joshi J.B., Pangarkar V.G. (1994). Sorption and permeation of aqueous picolines in elastomeric membranes. J. Membr. Sci..

[187] Te Hennepe H.J.C., Bargeman D., Mulder M.H.V., Smolders C.A. (1987). Zeolite -filled silicone rubber membranes. Part-I, Membrane preparation and pervaporation results. J. Membr. Sci..

[188] Jia M.D., Peinemann K.V., Behling R.D. (1992). Preparation and characterization of thin film zeolite-PDMS composite membrane. J. Membr. Sci..

[189] Netke S.A., Sawant S.B., Joshi J.B., Pangarkar V.G. (1995). Sorption and permeation of acetic acid through zeolite filled membranes. J. Membr. Sci..

[190] Rom A., Friedl A. (2016). Investigation of pervaporation performance of POMS membrane during separation of butanol from water and the effect of added acetone and ethanol. Sep. Purif. Technol..

[191] Masuda T., Tang B., Ingashimura T. (1986). Ethanol-water separation by pervaporation through substituted polyacetylene membranes. Polym. J..

[192] Salter C.S., Hickey P.J., Juricic F.P. (1990). Pervaporation of aqueous ethanol mixtures through poly (dimethyl siloxane) membrane. Sep. Sci. Technol..

[193] Volkov V.V., Khotimskii V.S., Plate N., Backish R. (1989). Organophilic polymers for pervaporation. Proceedings of the Fourth International Conference on Pervaporation Processes in the Chemical Industry.

[194] Kashiwagi T., Okabe K., Okita K. (1988). Separation of ethanol from ethanol/water mixtures by plasma-polymerized membranes from silicone compounds. J. Membr. Sci..

[195] Kabra M.M., Netke S.A., Sawant S.B., Joshi J.B., Pangarkar V.G. (1995). Pervaporative separation of carboxylic acid-water mixtures. Sep. Purif. Technol..

[196] Lau W.W.Y., Finlayson J., Dickson J.M., Jiang J., Brook M.A. (1997). Pervaporation performance of oligosilylstyrene-polydimethylsiloxane membrane for separation of organics from water. J. Membr. Sci..

[197] Ikegami T., Yanagishita H., Kitamoto D., Negishi H., Haraya K., Sano T. (2002). Concentration of fermented ethanol by pervaporation using silicalite membranes coated with silicone rubber. Desalination.

[198] Boddeeker K.W., Bengtson G., Pingel H. (1990). Pervaporation of isomeric butanols. J. Membr. Sci..

[199] Matsumoto Y., Kondo M., Fuzita Y., Backish R. (1992). Transport mechanism in PEBA membrane. Proceedings of the Sixth International Conference on Pervaporation Processes in the Chemical Industry.

[200] Peterson E.S., Stone M.L., Baver W.F., Gianotto A.K., Aimer P., Aptel P. (1992). The removal of organic chemicals from waste stream using polyphosphazene membranes. Proceedings of the Euromembrane.

[201] Nakagawa T., Hoshi M., Higuchi A., Backish R. (1991). Separation of aqueous organic solvents through poly(acrylic acid ester-*co*-acrylic acid) membranes by pervaporation. Proceedings of the 5th International Conference on Pervaporation Processes in the Chemical Industry.

[202] Pithan F., Staudt-Bickel C., Lichtenthaler R.N. (2002). Synthesis of highly fluorinated copolyimide membranes for the removal of high boiling organics from process water and wastewater by pervaporation. Desalination.

[203] Uragami T., Yamada H., Miyata T. (2001). Removal of dilute volatile organic compounds in water through graft copolymer membranes consisting of poly(alkylmethacrylate) and poly(dimethylsiloxane) by pervaporation and their membrane morphology. J. Membr. Sci..

[204] Uragami T., Fukuyama E., Miyata T. (2016). Selective removal of dilute benzene from water by poly(methyl methacrylate)-graft-poly(dimethyl siloxane) membranes containing hydrophobic ionic liquid by pervaporation. J. Membr. Sci..

[205] Jou J.D., Yoshida W., Cohen Y. (1999). A novel ceramic-supported polymer membrane for pervaporation of dilute volatile organic compounds. J. Membr. Sci..

[206] Jian K., Pintauro P.N., Ponangi R. (1996). Separation of dilute organic/water mixtures with asymmetric poly(vinylidene fluoride) membranes. J. Membr. Sci..

[207] Chen W.-J., Aranda P., Martin C.R. (1995). Pervaporation separation of ethanol/water mixtures by polystyrenesulfonate/alumina composite membranes. J. Membr. Sci..

[208] Santoro S., Galiano F., Jansen J.C., Figoli A. (2017). Strategy for scale-up of SBS pervaporation membranes for ethanol recovery from diluted aqueous solutions. Sep. Purif. Technol..

[209] Hosseini M., Ameri E. (2017). Pervaporation characteristics of a PDMS/PMHS membrane for removal of dimethyl sulfoxide from aqueous solutions. Vacuum.

[210] Hu K., Nie L., Liu J., Zheng J. (2013). Separation of methanol from methanol/water mixtures with pervaporation hybrid membranes. J. Appl. Polym. Sci..

[211] Toth A.J., Mizsey P. (2015). Methanol removal from aqueous mixture with organophilic pervaporation: Experiments and modelling. Chem. Eng. Res. Des..

[212] Yi S., Wan Y. (2017). Volatile organic compounds (VOCs) recovery from aqueous solutions via pervaporation with vinyltriethoxysilane-grafted-silicalite-1/polydimethylsiloxane mixed matrix membrane. Chem. Eng. J..

[213] Uragami T., Matsuoka Y., Miyata T. (2016). Permeation and separation characteristics in removal of dilute volatile organic compounds from aqueous solutions through copolymer membranes consisted of poly(styrene) and poly(dimethylsiloxane) containing a hydrophobic ionic liquid by pervaporation. J. Membr. Sci..

[214] Davey C.J., Leak D., Patterson D.A. (2016). Hybrid and mixed matrix membranes for separations from fermentations. Membranes.

[215] Vane L.M., Namboodiri V.V., Meier R.G. (2010). Factors affecting alcohol-water pervaporation performance of hydrophobic zeolite-silicone rubber mixed matrix membranes. J. Membr. Sci..

[216] Vane L.M., Namboodiri V.V., Bowen T.C. (2008). Hydrophobic zeolite-silicone rubber mixed matrix membranes for ethanol-water separation: Effect of zeolite and silicone component selection on pervaporation performance. J. Membr. Sci..

[217] Zhan X., Lu J., Tan T.T., Li J.D. (2012). Mixed matrix membranes with HF acidetched ZSM-5 for ethanol/water separation: Preparation and pervaporation performance. Appl. Surf. Sci..

[218] Evcin A., Tutkun O. (2009). Pervaporation separation of ethanol-water mixtures by zeolite-filled polymeric membranes. Ceramics-Silikaty.

[219] Gu J., Shi X., Bai Y.X., Zhang H.M., Zhang L., Huang H. (2009). Silicalite-filled polyether block-amides membranes for recovering ethanol from aqueous solution by pervaporation. Chem. Eng. Technol..

[220] Yi S.L., Su Y., Wan Y.H. (2010). Preparation and characterization of vinyl triethoxysilane (VTES) modified silicalite-1/PDMS hybrid pervaporation membrane and its application in ethanol separation from dilute aqueous solution. J. Membr. Sci..

[221] Liu X.L., Li Y.S., Liu Y., Zhu G.Q., Liu J., Yang W.S. (2011). Capillary sup-ported ultrathin homogeneous silicalite-poly(dimethyl siloxane)NCM for bio-butanol recovery. J. Membr. Sci..

[222] Huang Y., Zhang P., Fu J., Zhou Y., Huang X., Tang X. (2009). Pervaporation of ethanol aqueous solution by polydimethylsiloxane/polyphosphazene nanotube NCMs. J. Membr. Sci..

[223] Beltran A.B., Nisola G.M., Choi S.S., Kim Y., Chung W.J. (2013). Surface-functionalized silica NPs as fillers in polydimethylsiloxane membrane for the pervaporative recovery of 1-butanol from aqueous solution. J. Chem. Technol. Biotechnol..

[224] Zhou H., Shi R., Jin W. (2014). Novel organic–inorganic pervaporation membrane with a superhydrophobic surface for the separation of ethanol from an aqueous solution. Sep. Purif. Technol..

[225] Le N.L., Wang Y., Chung T.S. (2011). Pebax/POSS mixed matrix membranesfor ethanol recovery from aqueous solutions via pervaporation. J. Membr. Sci..

[226] Claes S., Vandezande P., Mullens S., De Sitter K., Peeters R., VanBael M.K. (2012). Preparation and benchmarking of thin film supported PTMSP-silica pervaporation membranes. J. Membr. Sci..

[227] Liu S.N., Liu G.P., Zhao X.H., Jin W.Q. (2013). Hydrophobic-ZIF-71 filled PEBA mixed matrix membranes for recovery of biobutanol via pervaporation. J. Membr. Sci..

[228] Yin H., Lau C.Y., Rozowski M., Howard C., Xu Y., Lai T., Dose M.E., Lively R.P., Lind M.L. (2017). Free-standing ZIF-71/PDMS NCMs for the recovery of ethanol and 1-butanol from water through pervaporation. J. Membr. Sci..

[229] Zhang C.F., Yang L., Bai Y.X., Gu J., Sun Y.P. (2012). ZSM-5 filled polyurethane urea membranes for pervaporation separation isopropyl acetate from aqueous solution. Sep. Purif. Technol..

[230] Liu X.L., Jin H., Li Y.S., Bux H., Hu Z.Y., Ban Y.J., Yang W. (2013). Metal-organic framework ZIF-8 NCM for efficient recovery of furfural via pervaporation and vapor permeation. J. Membr. Sci..

[231] Wessling M., Werner U., Huang S.T. (1991). Pervaporation of aromatic C_8_–isomers. J. Membr. Sci..

[232] McCandless F.P. (1973). Separation of aromatics and napthalenes by penneation through modified vinyledene fluoride films. Ind. Eng. Chem. Process Des. Dev..

[233] Cabasso I. (1983). Organic liquid mixtures separation by permselective polymer membranes. I. Selection and characteristics of dense isotropic membranes employed in the pervaporation process. Ind. Eng. Chem. Prod. Res. Dev..

[234] Ray S., Ray S.K. (2006). Separation of organic mixtures by pervaporation using crosslinked rubber membranes. J. Membr. Sci..

[235] Bruschke H.E.A., Schneider W.H., Scholz W.H., Steinhauser H., Backish R. (1992). Removal of methanol from organic mixtures. Proceedings of the 6th International Conference on Pervaporation Process in the Chemical Industry.

[236] Ray S.K., Sawant S.B., Joshi J.B., Pangarkar V.G. (1999). Methanol selective membranes for separation of methanol-ethylene glycol mixtures by pervaporation. J. Membr. Sci..

[237] Ray S.K., Sawant S.B., Pangarkar V.G. (1999). Separation of Methyl-tert-butyl alcohol (MTBE)-Methanol by pervaporation. J. Appl. Polym. Sci..

[238] Dutta B.K., Sikdar S.K. (1991). Separation of azeotropic organic liquid mixtures by pervaporation. AICHE J..

[239] Ray S., Ray S.K. (2006). Synthesis of highly methanol selective membranes for separation of methyl tertiary butyl ether (MTBE)–methanol mixtures by pervaporation. J. Membr. Sci..

[240] Gozzelino G., Malucelli G. (2004). Permeation of methanol/methyl-t-butyl ether mixtures through poly(ethylene-*co*-vinyl acetate) films. Colloids Surf. A Physicochem. Eng. Asp..

[241] Shi B., Wu Y., Liu J. (2004). Vapor permeation separation of MeOH/MTBE through polyimide/sulfonated poly(ether-sulfone) hollow-fiber membranes. Desalination.

[242] Yoshikawa M., Yoshioka T., Fujime J., Murakami A. (2000). Pervaporation separation of MeOH/MTBE through agarose membranes. J. Membr. Sci..

[243] Shah V.M., Bartels C.R., Pasternak M., Reale J. (1989). Opportunities for membranes in the production of octane enhancers. AIChE Symp. Ser..

[244] Khayet M., Nasef M.M., Mengual J.I. (2005). Radiation grafted poly(ethylene terephthalate)-graft-polystyrene pervaporation membranes for organic/organic separation. J. Membr. Sci..

[245] Volkov A.V., Volkov V.V., Khotimskii V.S. (2009). Membranes Based on poly[(1-Trimethylsilyl)-1-Propyne] for Liquid-Liquid Separation. Polym. Sci. Ser. A.

[246] Kameda M., Ohi K., Yoshimi Y., Kanamori T. (1999). Pervaporative separation of organic mixtures using dinitrophenyl group-containing cellulose acetate membrane. J. Membr. Sci..

[247] Liang L., Dickson J.M., Jiang J., Brook M.A. (2004). Effect of low flow rate on pervaporation of 1,2-dichloroethane with novel polydimethylsiloxane composite membranes. J. Membr. Sci..

[248] Cunha V.S., Paredes M.L.L., Borges C.P., Habert A.C., Nobrega R. (2002). Removal of aromatics from multicomponent organic mixtures by pervaporation using polyurethane membranes: Experimental and modeling. J. Membr. Sci..

[249] Cao B., Hinode H., Kajiuchi T. (1999). Permeation and separation of styrene/ethylbenzene mixtures through cross-linked poly(hexamethylene sebacate) membranes. J. Membr. Sci..

[250] Zhou M., Persin M., Sarrazin J. (1996). Methanol removal from organic mixtures by pervaporation using polypyrrole membranes. J. Membr. Sci..

[251] Villegas M., Romero A.I., Parentis M.L., Vidaurre E.F.C., Gottifredi J.C. (2016). Acrylic acid plasma polymerizedpoly(3-hydroxybutyrate) membranes formethanol/MTBE separation by pervaporation. Chem. Eng. Res. Des..

[252] Murthy Z.V.P., Shah M.K. (2017). Separation of isopropyl alcohol–toluene mixtures by pervaporation using poly(vinyl alcohol) membrane. Arab. J. Chem..

[253] Ribeiro C.P., Freeman B.D., Kalik D.S., Kalakkunnath S. (2012). Aromatic polyimide and polybenzoxazole membranes for the fractionation of aromatic/aliphatic hydrocarbons by pervaporation. J. Membr. Sci..

[254] Bowen T.C., Meier R.G., Vane L.M. (2007). Stability of MFI zeolite-filled PDMS membranes during pervaporative ethanol recovery from aqueous mixtures containing acetic acid. J. Membr. Sci..

[255] Patil M.B., Aminabhavi T.M. (2008). Pervaporation separation of toluene/alcohol mixtures using silicalite zeolite embedded chitosan mixed matrix membranes. Sep. Purif. Technol..

[256] Shao P., Kumar A. (2009). Separation of 1-butanol/2,3-Butanediol using ZSM-5 zeolite-filled polydimethylsiloxane membranes. J. Membr. Sci..

[257] Tamaddondar M., Pahlavanzadeh H., Hosseini S.S., Ruan G., Tan N.R. (2014). Self-assembled polyelectrolyte surfactant NCMs for pervaporation separation of MeOH/MTBE. J. Membr. Sci..

[258] Aouinti L., Roizard D., Belbachir M. (2015). PVC–activated carbon based matrices: A promising combination for pervaporation membranes useful for aromatic–alkane separations. Sep. Purif. Technol..

[259] Peng F., Pan F., Sun H., Lu L., Jiang Z. (2007). Novel nanocomposite pervaporation membranes composed of poly(vinyl alcohol) and chitosan-wrapped carbon nanotube. J. Membr. Sci..

[260] Shen J.N., Chu Y.X., Ruan H.M., Wu L.G., Gao C.J., Bruggen B.V. (2014). Pervaporation of benzene/cyclohexane mixtures through mixed matrix membranes of chitosan and Ag^+^/carbon nanotubes. J. Membr. Sci..

[261] Penkova A.V., Polotskaya G.A., Gavrilova V.A., Toikka A.M., Liu J.C., Trchova M., Slouf M., Pientka Z. (2010). PAm membranes modified by carbon nanotubes: Application for pervaporation. Sep. Sci. Technol..

[262] Kim H., Kim Y., Kim J., Lee S., Kang Y., Chin C. (2001). Spectroscopic characterization of cellulose acetate polymer membranes containing Cu(1,3-butadiene)OTf as a facilitated olefin transport carrier. Chem. Mater..

[263] Shen J., Wu L., Chen H., Gao C. (2005). Separation cyclohexene/cyclohexane mixtures with facilitated transport membrane of poly(vinyl alcohol)-Co^2+^. Sep. Purif. Technol..

[264] Wu L., Wang T., Jiang Z. (2013). Formation of AgCl nanoparticle in reverse micro-emulsion using polymerizable surfactant and the resulting copolymer hybrid membranes. J. Membr. Sci..

[265] Tor A., Arslan G., Muslu H., Celiktas A., Cengeloglu Y., Ersoz M. (2009). Facilitated transport of Cr(III) through polymer inclusion membrane with di(2-ethyl-hexyl) phosphoric acid (DEHPA). J. Membr. Sci..

[266] Zhang Y., Wang N., Zhao C., Wang L., Ji S., Li J.-R. (2016). Co(HCOO)_2_-based hybrid membranes for the pervaporation separation of aromatic/aliphatic hydrocarbon mixtures. J. Membr. Sci..

[267] Kim J., Kang S., Mun S., Kang Y. (2009). Facile synthesis of copper nanoparticles by ionic liquids and its application to facilitated olefin transport membranes. Ind. Eng. Chem. Res..

[268] Lu L., Sun H., Peng F., Jiang Z. (2006). Novel graphite-filled PVA/CS hybrid membrane for pervaporation of benzene/cyclohexane mixtures. J. Membr. Sci..

[269] Li Z., Zhang B., Qu L., Ren J., Li Y. (2011). A novel atmospheric dielectric barrier discharge (DBD) plasma graft-filling technique to fabricate the composite membranes for pervaporation of aromatic/aliphatic hydrocarbons. J. Membr. Sci..

[270] Wang N., Ji S., Li J., Zhang R., Zhang G. (2014). Poly(vinyl alcohol)–graphene oxide nanohybrid “pore-filling” membrane for pervaporation of toluene/n-heptane mixtures. J. Membr. Sci..

[271] Dai S.-Q., Jiang T.-Y., Wang T., Wu L.-G., Yu X.-Y., Lin J.-Z., Shi S.-X.-X. (2016). Enhanced performance of polyimide hybrid membranes for benzene separation by incorporating three-dimensional silver–graphene oxide. J. Colloid Interf. Sci..

[272] Gao Z., Yue Y., Li W. (1996). Application of zeolite-filled pervaporation membrane. Zeolites.

[273] Mali M.G., Magalad V.T., Gokavi G.S., Aminabhavi T.M., Raju K.V.S.N. (2011). Pervaporation separation of isopropanol-water mixtures using mixed matrix blend membranes of poly(vinylalcohol)/poly(vinylpyrrolidone) loaded with phosphomolybdic acid. J. Appl. Polym. Sci..

[274] Li C.L., Huang S.H., Hung W.S., Kao S.T., Wang D.M., Jean Y.C., Lee K.R., Lai J.Y. (2008). Study on the influence of the free volume of hybrid membrane on PV performance by positron annihilation spectroscopy. J. Membr. Sci..

[275] Prasad C.V., Yeriswamy B., Sudhakar H., Sudhakara P., Subha M.C.S., Rao J.I.K.C. (2012). Preparation and characterization of NP-filled, mixed-matrix membranes for the PV dehydration of isopropyl alcohol. J. Appl. Polym. Sci..

[276] Weng T., Tseng H., Wey M. (2009). Preparation and characterization of multi-walled carbon nanotube/PBNPI nanocomposite membrane for H_2_/CH_4_ separation. Int J. Hydrog. Energy.

[277] Bae T., Kim I., Tak T. (2006). Preparation and characterization of fouling-resistant TiO_2_ self-assembled nanocomposite membranes. J. Membr. Sci..

[278] Vatanpour V., Madaeni S.S., Khataee A.R., Salehi E., Zinadini S., Monfared H.A. (2012). TiO_2_ embedded mixed matrix PES nanocomposite membranes: Influence of different sizes and types of nanoparticles on antifouling and performance. Desalination.

[279] Solè I., Pey C.M., Maestro A., González C., Porras M., Solans C., Gutiérrez J.M. (2010). Nano-emulsions prepared by the phase inversion composition method: Preparation variables and scale up. J. Colloid Interf. Sci..

[280] Li P., Chen H.Z., Chung T.S. (2013). The effects of substrate characteristics and pre wetting agents on PAN-PDMS composite hollow fiber membranes for CO_2_/N_2_ and O_2_/N_2_ separation. J. Membr. Sci..

[281] Yave W., Car A., Funari S.S., Nunes S.P., Peinemann K.V. (2010). CO_2_-Philic polymer membrane with extremely high separation performance. Macromolecules.

[282] Ding J., Zhang M., Jiang Z., Li Y., Ma J., Zhao J. (2012). Enhancing the permselectivity of pervaporation membrane by constructing the active layer through alternative self-assembly and spin-coating. J. Membr. Sci..

[283] Zhao C., Jiang Z., Zhao J., Cao K., Zhang Q., Pan F. (2014). High pervaporation Dehydration Performance of the Composite Membrane with an Ultrathin Alginate/Poly(acrylic acid)−Fe_3_O_4_ Active Layer. Ind. Eng. Chem. Res..

[284] Wang X., Chen X., Yoon K., Fang D., Hsiao B.S., Chu B. (2005). High flux filtration medium based on nanofibrous substrate with hydrophilic nanocomposite coating. Environ. Sci. Technol..

[285] Steele A., Bayer I., Loth E. (2009). Inherently superoleophobic nanocomposite coatings by spray atomization. Nano Lett..

[286] Genne I., Kuypers S., Leysen R. (1996). Effect of the addition of ZrO_2_ to polysulfone based UF membranes. J. Membr. Sci..

[287] Wara N.M., Francis L.F., Velamakanni B.V. (1995). Addition of alumina to cellulose acetate membranes. J. Membr. Sci..

[288] Zou H., Wu S., Shen J. (2008). Polymer/silica nanocomposites: Preparation, characterization, properties, and applications. Chem. Rev..

[289] Li Q., He R., Jens O.J., Bjerrum N.J. (2003). Approaches and recent development of polymer electrolyte membranes for fuel cell operating above 100 °C. Chem. Mater..

[290] Mascia L., Zhang Z. (1996). Carbon fibre composites based on polyimide/siIica ceramers: Aspects of structure-properties relationship. Compos. Part A Appl. Sci. Manuf..

[291] Pramanik M., Srivastava S.K., Samantaray B.K., Bhowmick A.K. (2003). Rubber–clay nanocomposite by solution blending. J. Appl. Polym. Sci..

[292] Filippi S., Mameli E., Marazzato C., Magagnini P. (2007). Comparison of solution-blending and melt-intercalation for the preparation of poly(ethylene-*co*-acrylic acid)/organoclay nanocomposites. Eur. Polym. J..

[293] Wang D., Wilkie C.A. (2002). Preparation of PVC-clay nanocomposites by solution blending. J. Vinyl Addit. Technol..

[294] Madaleno L., Schjødt-Thomsen J., Pinto J.C. (2010). Morphology, thermal and mechanical properties of PVC/MMT nanocomposites prepared by solution blending and solution blending + melt compounding. Compos. Sci. Technol..

[295] Pramanik M., Srivastava S.K., Samantaray B.K., Bhowmick A.K. (2003). EVA/clay nanocomposite by solution blending: Effect of aluminosilicate layers on mechanical and thermal properties. Macromol. Res..

[296] Liu L., Pu C., Viswanathan R., Fan Q., Liu R., Smotkin E.S. (1998). Carbon supported and unsupported Pt–Ru anodes for liquid feed direct methanol fuel cells. Electrochim. Acta.

[297] Hayden E. (1997). The promotion of CO electro-oxidation on platinumbismuth as a model for surface mediated oxygen transfer. Catal. Today.

[298] Page T., Johnson R., Hormes J., Noding S., Rambabu B. (2000). A study of methanol electro-oxidation reactions in carbon membrane electrodes and structural properties of Pt alloy electro-catalysts by EXAFS. J. Electroanal. Chem..

[299] Mahon H.I. (1966). Permeability Separatory Apparatus, Permeability Separatory Membrane Element, Method of Making the Same and Process Utilizing the Same. U.S. Patent.

[300] Sukitpaneenit P., Chung T. (2012). PVDF/nanosilica dual-layer hollow fibers with enhanced selectivity and flux as novel membranes for ethanol recovery. Ind. Eng. Chem. Res..

[301] Peng N., Widjojo N., Sukitpaneenit P., Teoh M.M., Lipscomb G.G., Chung T.S., Lai J.Y. (2012). Evolution of polymeric hollow fibers as sustainable technologies: Past, present, and future. Prog. Polym. Sci..

[302] Ong Y.K., Chung T.S. (2013). Pushing the limits of high performance dual-layer hollow fiber fabricated via I2PS process in dehydration of ethanol. AICHE J..

[303] Liu Y.-L., Yu C.-H., Ma L.-C., Lin G.-C., Tsai H.-A., Lai J.-Y. (2008). The effects of surface modifications on preparation and pervaporation dehydration performance of chitosan/polysulfone composite hollow-fiber membranes. J. Membr. Sci..

[304] Hua D., Ong Y.K., Wang P., Chung T.-S. (2014). Thin-film composite tri-bore hollow fiber (TFCTbHF) membranes for isopropanol dehydration by pervaporation. J. Membr. Sci..

[305] Zhao L., Ho W.S.W. (2014). Novel reverse osmosis membranes incorporated with a hydrophilic additive for seawater desalination. J. Membr. Sci..

[306] Roy S., Ntim S.A., Mitra S., Sirkar K.K. (2011). Facile fabrication of superior nanofiltration membranes from interfacially polymerized CNT-polymer composites. J. Membr. Sci..

[307] Wang H., Zhang Q., Zhang S. (2011). Positively charged nanofiltration membrane formed by interfacial polymerization of 3,3′,5,5′-biphenyl tetraacyl chloride and piperazine on a poly (acrylonitrile) (PAN) support. J. Membr. Sci..

[308] Morgan P.W. (1965). Condensation Polymers: By Interfacial and Solution Methods.

[309] Cadotte J.E., Petersen R.J., Larson R.E., Erickson E.E. (1980). A new thin-film composite seawater reverse osmosis membrane. Desalination.

[310] Cadotte J.E., Lloyd D.R. (1985). Evolution of Composite Reverse Osmosis Membranes. Materials Science of Synthetic Membranes.

[311] Parthasarathy A., Brumlik C.J., Martin C.R., Collins G.E. (1994). Interfacial polymerization of thin polymer films onto the surface of a microporous hollow-fiber membrane. J. Membr. Sci..

[312] Kim J.H., Lee K.H., Kim S.Y. (2000). Pervaporation separation of water from ethanol through polyimide composite membranes. J. Membr. Sci..

[313] Liu Y.L., Yu C.H., Lai J.Y. (2008). Poly (tetrafluoroethylene)/PAm thin-film composite membranes via interfacial polymerization for pervaporation dehydration on an isopropanol aqueous solution. J. Membr. Sci..

[314] Zuo J., Wang Y., Sun S.P., Chung T.S. (2012). Molecular design of thin film composite (TFC) hollow fiber membranes for isopropanol dehydration via pervaporation. J. Membr. Sci..

[315] Albo J., Wang J., Tsuru T. (2014). Application of interfacially polymerized PAm composite membranes to isopropanol dehydration: Effect of membrane pre-treatment and temperature. J. Membr. Sci..

[316] Shawky H.A., Chae S., Lin S., Wiesner M.R. (2011). Synthesis and characterization of a carbon nanotube/polymer NCM for water treatment. Desalination.

[317] Kim E.S., Hwang G., El-Din M.G., Liu Y. (2012). Development of nanosilver and multi-walled carbon nanotubes thin-film NCM for enhanced water treatment. J. Membr. Sci..

[318] Fathizadeh M., Aroujalian A., Raisi A. (2011). Effect of added NaX nano-zeolite into PAm as a top thin layer of membrane on water flux and salt rejection in a reverse osmosis process. J. Membr. Sci..

[319] Singh P.S., Aswal V.K. (2008). Characterization of physical structure of silica nanoparticles encapsulated in polymeric structure of PAm films. J. Colloid Interf. Sci..

[320] Lee S.Y., Kim H.J., Patel R., Im S.J., Kim J.H., Min B.R. (2007). Silver nanoparticles immobilized on thin film composite PAm membrane: Characterization, nanofiltration, antifouling properties. Polym. Adv. Technol..

[321] Huang S., Liu Y., Huang Y., Liao K., Hu C., Lee K., Lai J. (2014). Study on characterization and pervaporation performance of Interfacially polymerized PAm thin-film composite Membranes for dehydrating tetrahydrofuran. J. Membr. Sci..

[322] Morgan P.W., Kwolek S.L. (1959). Interfacial polycondensation. II. Fundamentals of polymer formation at liquid interfaces. J. Polym. Sci..

[323] La Y., Sooriyakumaran R., Miller D.C., Fujiwara M., Terui Y., Yamanaka K., McCloskey B.D., Freeman B.D., Allen R.D. (2010). Novel thin film composite membrane containing ionizable hydrophobes: PH dependent reverse osmosis behavior and improved chlorine resistance. J. Mater. Chem..

[324] Jeong B.H., Hoek E.M.V., Yan Y., Subramani A., Huang X., Hurwitz G., Ghosh A.K., Jawor A. (2007). Interfacial polymerization of thin film nanocomposites: A new concept for reverse osmosis membranes. J. Membr. Sci..

[325] Freger V. (2003). Nanoscale heterogeneity of PAm membranes formed by interfacial polymerization. Langmuir.

[326] Song Y., Sun P., Henry L.L., Sun B. (2005). Mechanisms of structure and performance controlled thin film composite membrane formation via interfacial polymerization process. J. Membr. Sci..

[327] Zhang Q.G., Liu Q.L., Chen Y., Chen J.H. (2007). Dehydration of isopropanol by novel poly(vinyl alcohol)-silicone hybrid membranes. Ind. Eng. Chem. Res..

[328] Naidu B.V.K., Sairam M., Raju K.V.S.N., Aminabhavi T.M. (2005). Pervaporation separation of water+ isopropanol mixtures using novel nanocomposite membranes of poly (vinyl alcohol) and polyaniline. J. Membr. Sci..

[329] Varghese J.G., Kittur A.A., Kariduraganavar M.Y. (2009). Dehydration of THF–water mixtures using zeolite-incorporated polymeric membranes. J. Appl. Polym. Sci..

[330] Sairam M., Naidua B.V.K., Nataraj S.K., Sreedhar B., Aminabhavi T.M. (2006). Poly(vinylalcohol)iron oxide nanocomposite membranes for pervaporation dehydration of isopropanol,1,4-dioxane and tetrahydrofuran. J. Membr. Sci..

[331] Urtiaga A., Gorri E.D., Casado C., Ortiz I. (2003). Pervaporative dehydration of industrial solvents using azeolite NaA commercial membrane. Sep. Purif. Technol..

[332] Shirazi Y., Ghadimi A., Mohammadi T. (2012). Recovery of alcohols from water using polydimethylsiloxane-silica NCMs: Characterization and pervaporation performance. J. Appl. Polym. Sci..

[333] Zhang Q.G., Liu Q.L., Jiang Z.Y., Chen Y. (2007). Anti-trade-off in dehydration of ethanol by novel PVA/APTEOS hybrid membranes. J. Membr. Sci..

[334] Gallego-Lizon T., Edwards E., Lobiundo G., Freitas dos Santos L. (2002). Dehydration of water/t-butanol mixtures by pervaporation: Comparative study of commercially available polymeric microporous silica and zeolite membranes. J. Membr. Sci..

[335] Shah D., Kissich K., Ghorpade A., Hannah R., Bhattacharyya D. (2000). Pervaporation of alcohol-water and dimethylformamide-water mixtures using hydrophilic zeolite NaA membranes: Mechanisms and experimental results. J. Membr. Sci..

[336] Kita H., Asamura H., Tanaka K., Okamoto K., Pinnau I., Freeman B.D. (1999). Preparation and pervaporation properties of X- and Y-type zeolite membranes. Membrane Formation and Modification.

[337] Kulprathipanja S., Neuzil R.W., Li N.N. (1988). Separation by Means of Mixed Matrix Membranes. U.S. Patent.

[338] Te Hennepe H.J.C., Smolders C.A., Bargeman D., Mulder M.H.V. (1991). Exclusion and tortuosity effects for alcohol/water separation by zeolite filled PDMS membranes. Sep. Sci. Technol..

[339] Zhang Q.G., Liu Q.L., Zhu A.M., Xiong Y., Zhang X.H. (2008). Characterization and permeation performance of novel organic-inorganic hybrid membranes of poly(vinyl alcohol)/1,2-bis(triethoxysilyl)ethane. J. Phys. Chem. B.

[340] Peng P., Shi B.L., Lan Y.Q. (2011). Preparation of PDMS-silica NCMs with silane coupling for recovering ethanol by pervaporation. Sep. Sci. Technol..

[341] Jiang L.Y., Chung T.S., Rajagopalan R. (2007). Matrimids/MgO mixed matrix membranes for pervaporation. AIChE J..

[342] Kim K.J., Park S.H., So W.W., Moon S.J. (2001). Pervaporation separation of aqueous organic mixtures through sulfated zirconia-poly(vinyl alcohol) membrane. J. Appl. Polym. Sci..

[343] Suhas D.P., Aminabhavi T.M., Raghu A.V. (2013). Mixed matrix membranes of H-ZSM5-loaded poly(vinyl alcohol) used in pervaporation dehydration of alcohols: Influence of silica/alumina ratio. Polym. Eng. Sci..

[344] Jiang L.Y., Chung T.S. (2010). Homogeneous polyimide/cyclodextrin composite membranes for pervaporation dehydration of isopropanol. J. Membr. Sci..

[345] Khosravi T., Mosleh S., Bakhtiari O., Mohammadi T. (2012). Mixed matrix membranes of Matrimid 5218 loaded with zeolite 4A for pervaporation separation of water-isopropanol mixtures. Chem. Eng. Res. Des..

[346] Sorribas S., Kudasheva A., Almendro E. (2015). Pervaporation and membrane reactor performance of polyimide based mixed matrix membranes containing MOF HKUST-1. Chem. Eng. Sci..

[347] Yan H., Li J., Fan H., Ji S., Zhang G., Zhang Z. (2015). Sonication-enhanced in situ assembly of organic/inorganic hybrid membranes: Evolution of nanoparticle distribution and pervaporation performance. J. Membr. Sci..

[348] Takamizawa S., Kachi-Terajima C., Kohbara M., Akatsuka T., Jin T. (2007). Alcohol-vapor inclusion in single-crystal adsorbents [MII2 (bza)4(pyz)]n (M=Rh, Cu): Structural study and application to separation membranes. Chemistry.

[349] Moermans B., Beuckelaer W.D., Vankelecom I.F.J., Ravishankar R., Martens J.A., Jacobs P.A. (2000). Incorporation of nano-sized zeolites in membranes. Chem. Commun..

[350] Han Y.J., Wang K.H., Lai J.Y., Liu Y.L. (2014). Hydrophilic chitosan-modified polybenzoimidazole membranes for pervaporation dehydration of isopropanol aqueous solutions. J. Membr. Sci..

[351] Phan A., Doonan C.J., Uribe-Romo F.J., Knobler C.B., O’Keeffe M., Yaghi O.M. (2009). Synthesis, structure, and carbondioxide capture properties of zeolitic imidazolate frameworks. Acc. Chem. Res..

[352] Albo J., Santos E., Neves L.A., Simeonov S.P., Afonso C.A.M., Crespo J.G., Irabien A. (2012). Separation performance of CO_2_ through supported magnetic ionic liquid membranes (SMILMs). Sep. Purif. Technol..

[353] Albo J., Tsuru T. (2014). Thin ionic liquid membranes based on inorganic supports with different pore sizes. Ind. Eng. Chem. Res..

[354] Jiang Y., Gou C., Liu H. (2006). Magnetically rotational reactor for absorbing benzene emissions by ionic liquids. China Particuol..

[355] Wang C., Guo Z.X., Fu S., Wu W., Zhu D. (2004). Polymers containing fullerene or carbon nanotube structures. Prog. Polym. Sci..

[356] Ajayan P.M. (1999). Nanotubes from carbon. Chem. Rev..

[357] Pekker S., Salvetat J.P., Jakab E., Bonard J.M., Forro L. (2001). Hydrogenation of carbon nanotubes and graphite in liquid ammonia. J. Phys. Chem. B.

[358] Lou X., Detrembleur C., Sciannamea V., Pagnoulle C., Jérôme R. (2004). Grafting of alkoxyamine end-capped (*co*)polymers onto multi-walled carbon nanotubes. Polymer.

[359] Mauter M.S., Wang Y., Okemgbo K.C., Osuji C.O., Giannelis E.P., Elimelech M. (2011). Antifouling ultrafiltration membranes via post-fabrication grafting of biocidal nanomaterials. ACS Appl. Mater. Interf..

[360] Liang S., Kang Y., Tiraferri A., Giannelis E.P., Huang X., Elimelech M. (2013). Highly hydrophilic polyvinylidene fluoride (PVDF) ultrafiltration membranes via postfabrication grafting of surface-tailored silica nanoparticles. ACS Appl. Mater. Interf..

[361] Shameli A., Ameri E. (2017). Synthesis of cross-linked PVA membranes embedded with multi-wall carbon nanotubes and their application to esterification of acetic acid with methanol. Chem. Eng. J..

[362] Ray S., Singha N.R., Ray S.K. (2009). Removal of tetrahydrofuran (THF) from water by pervaporation using homo and blend polymeric membranes. Chem. Eng. J..

[363] Singha N.R., Ray S.K. (2010). Separation of toluene-methanol mixtures by pervaporation using semi-IPN polymer membranes. Sep. Sci. Technol..

[364] Singha N.R., Kar S., Ray S.K. (2009). Synthesis of chemically modified polyvinyl alcohol membranes for dehydration of dioxane by pervaporation. Sep. Sci. Technol..

[365] Singha N.R., Kar S., Ray S.K. (2009). Synthesis of novel polymeric membrane for separation of MTBE-methanol by pervaporation. Sep. Sci. Technol..

[366] Kuila S.B., Ray S.K., Das P., Singha N.R. (2011). Synthesis of full interpenetrating network membranes of poly(acrylic acid-*co*-acrylamide) in the matrix of polyvinyl alcohol for dehydration of ethylene glycol by pervaporation. Chem. Eng. Process. Process Intensif..

[367] Samanta H.S., Ray S.K., Das P., Singha N.R. (2012). Separation of acid–water mixtures by pervaporation using nanoparticle filled mixed matrix copolymer membranes. J. Chem. Technol. Biotechnol..

[368] Ray S., Ray S.K. (2008). Dehydration of tetrahydrofuran (THF) by pervaporation using crosslinked copolymer membranes. Chem. Eng. Process. Process Intensif..

[369] Singha N.R., Kar S., Ray S., Ray S.K. (2009). Separation of isopropyl alcohol–water mixtures by pervaporation using crosslink IPN membranes. Chem. Eng. Process. Process Intensif..

[370] Karmakar M., Mahapatra M., Dutta A., Chattopadhyay P.K., Singha N.R. (2017). Fabrication of semisynthetic collagenic materials for mere/synergistic adsorption: A model approach of determining dye allocation by systematic characterization and optimization. Int. J. Biol. Macromol..

[371] Karmakar M., Mahapatra M., Singha N.R. (2017). Separation of tetrahydrofuran using RSM optimized accelerator-sulfur-filler of rubber membranes: Systematic optimization and comprehensive mechanistic study. Korean J. Chem. Eng..

[372] Albo J., Luis P., Irabien A. (2011). Absorption of coal combustion flue gases in ionic liquids using different membrane contactors. Desalination Water Treat..

[373] Albo J., Irabien A. (2012). Non-dispersive absorption of CO_2_ in parallel and cross-flow membrane modules using EMISE. J. Chem. Technol. Biotechnol..

[374] Albo J., Luis P., Irabien A. (2010). Carbon dioxide capture from flue gases using a cross-flow membrane contactor and the ionic liquid 1-Ethyl-3-methylimidazolium ethylsulfate. Ind. Eng. Chem. Res..

[375] Norkobilov A., Gorri D., Ortiz I. (2017). Process flowsheet analysis of pervaporation-based hybrid processes in the production of ethyl tert-butyl ether. J. Chem. Technol. Biotechnol..

[376] Tonkovich A.L.Y., Zilka J.L., Jimenez D.M., Roberts G.L., Cox J.L. (1996). Experimental investigations of inorganic membrane reactors: A distributed feed approach for partial oxidation reactions. Chem. Eng. Sci..

[377] Ramachandra A.M., Lu Y., Ma Y.H., Moser W.R., Dixon A.G. (1996). Oxidative coupling of methane in porous Vycor membrane reactors. J. Membr. Sci..

[378] Nozaki T., Fujimoto K. (1994). Oxide ion transport for selective oxidative coupling of methane with new membrane reactor. AICHE J..

[379] Guo X., Hidajat K., Ching C. (1997). Oxidative coupling of methane in a solid oxide membrane reactor. Ind. Eng. Chem. Res..

[380] Lu Y., Dixon A.G., Moser W.R., Ma Y., Balachandran U. (2000). Oxygen permeable dense membrane reactor for the oxidative coupling of methane. J. Membr. Sci..

[381] Kim J.H., Kang Y.S., Won J. (2004). Silver polymer electrolyte membranes for facilitated olefint transport: Carrier properties, transport mechanism and separation performance. Macromol. Res..

[382] Kapteijn F., Bakker W.J.W., Zheng G., Poppe J., Moulijn J.A. (1995). Permeation and separation of light hydrocarbons through a silicalite-1 membrane: Application of the generalized Maxwell-Stefan equations. Chem. Eng. J. BioChem. Eng. J..

[383] Uhlhorn R.J.R., Keizer K., Burggraaf A.J. (1992). Gas transport and separation with ceramic membranes. Part II. Synthesis and separation properties of microporous membranes. J. Membr. Sci..

[384] Ravanchi M.T., Kaghazchi T., Kargari A. (2009). Application of membrane separation processes in petrochemical industry: A review. Desalination.

[385] Krol J.J., Boerrigter M., Koops G.H. (2001). Polymeric hollow fiber gas separation embranes: Preparation and the suppression of plasticization in propane/propylene environments. J. Membr. Sci..

[386] Falbo F., Tasselli F., Brunetti A., Drioli E., Barbieri G. (2014). Polyimide hollow fiber membranes for CO_2_ separation from wet gas mixtures. Braz. J. Chem. Eng..

[387] Esche E., Müller D., Song S., Wozny G. (2015). Optimization during the process synthesis: Enabling the oxidative coupling of methane by minimizing the energy required for the carbon dioxide removal. J. Clean. Prod..

[388] Stünkel S. (2013). Kohlendioxid-Abtrennung in der Gasaufbereitung des Prozesses der Oxidativen Kupplung von Methan. Ph.D. Thesis.

[389] Song S., Esche E., Stünkel S., Brinkmann T., Wind J., Shishatskiy S., Wozny G. (2013). Energy, equipment and cost savings by using a membrane unit in an amine-based absorption process for CO_2_ removal. Chem. Ing. Technik.

[390] Van der Bruggen B. (2010). Pervaporation membrane reactors. Compr. Membr. Sci. Eng..

[391] Brunetti A., Barbieri G., Drioli E., Lee K., Sea B., Lee D. (2007). WGS reaction in a membrane reactor using a porous stainless steel supported silica membrane. Chem. Eng. Process. Process Intensif..

[392] Basile A., Drioli E., Santella F., Violante V., Capannelli G., Vitulli G. (1996). A study on catalytic membrane reactors for water gas shift reaction. Gas Sep. Purif..

[393] Barbieri G., Brunetti A., Granato T., Bernardo P., Drioli E. (2005). Engineering evaluations of a catalytic membrane reactor for the water gas shift reaction. Ind. Eng. Chem. Res..

[394] Brunetti A., Barbieri G., Drioli E., Granato T., Lee K. (2007). A porous stainless steel supported silica membrane for WGS reaction in a catalytic membrane reactor. Chem. Eng. Sci..

[395] Radcliffe A.J., Singh R.P., Berchtold K.A., Lima F.V. (2016). Modeling and optimization of high-performance polymer membrane reactor systems for water–gas shift reaction applications. Processes.

[396] Marcelo C.A., David L.F., Mergel J., Stolten D. (2013). A comprehensive review on PEM water electrolysis. Int. J. Hydrog. Energy.

[397] Barbir F. (2005). PEM electrolysis for production of hydrogen from renewable energy sources. Sol. Energy.

[398] Lin H., Van Wagner E., Freeman B.D., Toy L.G., Gupta R.P. (2006). Plasticization-enhanced hydrogen purification using polymeric membranes. Science.

[399] Shao L.L., Ting B., Shung T., Greenberg A.R. (2009). Polymeric membranes for the hydrogen economy: Contemporary approaches and prospects for the future. J. Membr. Sci..

[400] Jamshidi S., Noruzi A., Babaluo A.A., Haghighi M. (2016). Performance of Pd composite membrane prepared by organic–inorganic method in WGS membrane reactor. Sep. Sci. Technol..

[401] Basile A., Figoli A., Khayet M. (2015). Pervaporation, Vapour Permeation and Membrane Distillation: Principles and Applications.

[402] Inoue T., Nagase T., Hasegawa Y., Kiyozumi Y., Sato K., Nishioka M., Hamakawa S., Mizukami F. (2007). Stoichiometric Ester Condensation Reaction Processes by PervaporativeWater Removal via Acid-Tolerant Zeolite Membranes. Ind. Eng. Chem. Res..

[403] Ziobrowski Z., Kiss K., Rotkegel A., Nemest Othy N., Krupiczka R., Gubicza L. (2009). Pervaporation aided enzymatic production of glycerol monostearate in organic solvents. Desalination.

[404] Torabi B., Ameri E. (2016). Methyl acetate production by coupled esterification-reaction process using synthesized cross-linked PVA/silica NCMs. Chem. Eng. J..

[405] Penkova A., Polotskaya G., Toikka A. (2015). Pervaporation composite membranes for ethyl acetate production. Chem. Eng. Process. Process Intensif..

[406] Elimelech M., Phillip W.A. (2011). The future of seawater desalination: Energy, technology, and the environment. Science.

[407] Singh R.J. (2008). Worldwide water crisis. J. Membr. Sci..

[408] Korngold E., Korin E., Ladizhensky I. (1996). Water desalination by pervaporation with hollow fiber membranes. Desalination.

[409] Zwijnenberg H.J., Koops G.H., Wessling M. (2005). Solar driven membrane pervaporation for desalination processes. J. Membr. Sci..

[410] Korin E., Ladizhensky I., Korngold E. (1996). Hydrophilic hollow fiber membranes for water desalination by the pervaporation method. Chem. Eng. Process. Process Intensif..

[411] Cho C.H., Oh K.Y., Kim S.K., Yeo J.G., Sharma P. (2011). Pervaporative seawater desalination using NaA zeolite membrane: Mechanisms of high water flux and high salt rejection. J. Membr. Sci..

[412] Kuznetsov Y.P., Kruchinina E.V., Baklagina Y.G., Khripunov A.K., Tulupova O.A. (2007). Deep desalination of water by evaporation through polymeric membranes. Russ. J. Appl. Chem..

[413] Bolto B., Hoang M., Xie Z. (2010). Pervaporation- a further low energy desalination option?. Water AWA.

[414] Xie Z., Hoang M., Duong T., Ng D., Dao B., Gray S. (2011). Sol-gel derived poly(vinyl alcohol)/maleic acid/silica hybrid membrane for desalination by pervaporation. J. Membr. Sci..

[415] Liang B., Pan K., Li L., Giannelis E.P., Cao B. (2014). High performance hydrophilic pervaporation composite membranes for water desalination. Desalination.

[416] Cheng C., Shen L., Yu X., Yang Y., Li X., Wang X. (2017). Robust construction of graphene oxide barrier layer on nanofibrous substrate assisted by flexible poly(vinylalcohol) for efficient pervaporation desalination. J. Mater. Chem. A.

[417] Kim F., Cote L.J., Huang J.X. (2010). Graphene oxide: Surface activity and two-dimensional assembly. Adv. Mater..

[418] Akin I., Zor E., Bingol H., Ersoz M. (2014). Green synthesis of reduced graphene oxide/polyaniline composite and its application for salt rejection by polysulfone-based composite membranes. J. Phys. Chem. B.

[419] Tang Y.P., Paul D.R., Chung T.S. (2014). Free-standing graphene oxide thin films assembled by a pressurized UF method for dehydration of ethanol. J. Membr. Sci..

[420] Liang B., Zhan W., Qi G., Lin S., Nan Q., Liu Y., Cao B., Pan K. (2015). High performance graphene oxide/polyacrylonitrile composite pervaporation membranes for desalination applications. J. Mater. Chem. A.

[421] Chaudhri S.G., Rajai B.H., Singh P.S. (2015). Nanoscale homogeneity of silica-poly(vinyl alcohol) membranes by controlled cross-linking via sol-gel reaction in acidified and hydrated ethanol. RSC Adv..

[422] Feng B., Xu K., Huang A. (2017). Synthesis of graphene oxide/polyimide mixed matrix membranes for desalination. RSC Adv..

[423] Xie Z., Ng D., Hoang M., Duong T., Gray S. (2011). Separation of aqueous salt solution by pervaporation through hybrid organic-inorganic membrane: Effect of operating conditions. Desalination.

[424] Menz F.C., Seip H.M. (2004). Acid rain in Europe and the United States: An update. Environ. Sci. Policy.

[425] Lu Z., Streets D.G., Zhang Q., Wang S., Carmichael G.R., Cheng Y.F., Wei C., Chin M., Diehl T., Tan Q. (2010). Sulfur dioxide emissions in China and sulfur trends in East Asia since 2000. Atmos. Chem. Phys..

[426] Shariatinia Z., Jalali A.M., Taromi F.A. (2016). Molecular dynamics simulations on desulfurization of n-octane/thiophene mixture using silica filled polydimethylsiloxane NCMs. Model. Simul. Mater. Sci. Eng..

[427] Yamazaki K., Suzuki T., Takahashi N., Yokota K., Sugiura M. (2001). Effect of the addition of transition metals to Pt/Ba/Al_2_O_3_ catalyst on the NOx storage-reduction catalysis under oxidizing conditions in the presence of SO_2_. Appl. Catal. B Environ..

[428] Babich I.V., Moulijn J.A. (2003). Science and technology of novel processes for deep desulfurization of oil refinery streams: A review. Fuel.

[429] Brunet S., Mey D., Perot G., Bouchy C., Diehl F. (2005). On the hydrodesulfurization of FCC gasoline: A review. Appl. Catal. A Gen..

[430] Fihri A., Mahfouz R., Shahrani A., Taie I., Alabedi G. (2016). Pervaporative desulfurization of gasoline: A review. Chem. Eng. Process. Process Intensif..

[431] Lin L.G., Kong Y., Wang G., Qu H.M., Yang E.R., Shi D.Q. (2006). Selection and crosslinking modification of membrane material for FCC gasoline desulfurization. J. Membr. Sci..

[432] Kong Y., Lin L., Yang J., Shi D., Qu H., Xie K., Li L. (2007). FCC gasoline desulfurization by pervaporation: Effects of gasoline components. J. Membr. Sci..

[433] Rychlewska K., Konieczny K. (2014). Pervaporative desulfurization of gasoline—Separation of hydrocarbon/thiophene mixtures using polydimethylsiloxane (PDMS)-based membranes. Desalination Water Treat..

[434] Cao R., Zhang X., Wua H., Wang J., Liu X., Jiang Z. (2011). Enhanced pervaporative desulfurization by polydimethylsiloxane membranes embedded with silver/silica core–shell microspheres. J. Hazard. Mater..

[435] Li B., Yu S., Jiang Z., Liu W., Cao R., Wu H. (2012). Efficient desulfurization by polymer–inorganic NCMs fabricated in reverse microemulsion. J. Hazard. Mater..

[436] Yang D., Yang S., Jiang Z., Yu S., Zhang J., Pan F., Cao X., Wang B., Yang J. (2015). Polydimethylsiloxane–graphene nanosheets hybrid membranes with enhanced pervaporative desulfurization performance. J. Membr. Sci..

